# Design of 2‑Aminobenzothiazole Derivatives Targeting Trypanosomatid PTR1 by a Multidisciplinary Fragment Hybridization Approach

**DOI:** 10.1021/acs.jmedchem.5c01799

**Published:** 2025-09-30

**Authors:** Joanna Panecka-Hofman, Pasquale Linciano, Ina Pöhner, Edyta Dyguda-Kazimierowicz, Wiktoria Jedwabny, Giacomo Landi, Nuno Santarem, Gesa Witt, Bernhard Ellinger, Maria Kuzikov, Rosaria Luciani, Stefania Ferrari, Daniele Aiello, Stefano Mangani, Cecilia Pozzi, Anabela Cordeiro-da-Silva, Sheraz Gul, Maria Paola Costi, Rebecca C. Wade

**Affiliations:** † Molecular and Cellular Modeling Group, 40092 Heidelberg Institute for Theoretical Studies (HITS), D-69118 Heidelberg, Germany; ‡ Biophysics Division, Institute of Experimental Physics, Faculty of Physics, 49605 University of Warsaw, 02-093 Warsaw, Poland; § Department of Life Sciences, University of Modena and Reggio Emilia, 41121 Modena, Italy; ∥ Department of Drug Science, 19001 University of Pavia, 27100 Pavia, Italy; ⊥ School of Pharmacy, Faculty of Health Sciences, 205538 University of Eastern Finland, 70211 Kuopio, Finland; # Faculty of Chemistry, 49567 Wrocław University of Science and Technology, 50-370 Wrocław, Poland; ∇ Department of Biotechnology, Chemistry and Pharmacy, 9313 University of Siena, 53100 Siena, Italy; ○ Instituto de Investigação e Inovação em Saúde, 451168 Universidade do Porto and Institute for Molecular and Cell Biology, 4150-180 Porto, Portugal; ◆ 298192 Fraunhofer Institute for Translational Medicine and Pharmacology ITMP, Discovery Research ScreeningPort, D-22525 Hamburg, Germany; ¶ Consorzio Interuniversitario Risonanze Magnetiche di Metallo Proteine (CIRMMP), 50019 Sesto Fiorentino, Florence, Italy; & Center for Molecular Biology of Heidelberg University (ZMBH), DKFZ-ZMBH Alliance, and Interdisciplinary Center for Scientific Computing (IWR), Heidelberg University, D-69120 Heidelberg, Germany; ● Faculties of Engineering Sciences and Biosciences, Heidelberg University, D-69120 Heidelberg, Germany

## Abstract

Pteridine reductase 1 (PTR1) is a folate pathway enzyme essential for pathogenic trypanosomatids and a promising drug target for diseases such as sleeping sickness and leishmaniasis. Previous studies have shown that the 2-aminobenzothiazole moiety targets the PTR1 biopterin pocket, while 3,4-dichlorophenyl-containing compounds, such as **I** bind a different region of the *Trypanosoma brucei* PTR1 (*Tb*PTR1) pocket. This study combines both moieties via various linkers, creating two compound series screened in silico against *Tb*PTR1 and *Leishmania major* PTR1 (*Lm*PTR1). In the first series, five compounds were synthesized, and **1a** and **1b** emerged as potent *Tb*PTR1 inhibitors, with **1b** also being active against *Lm*PTR1 and moderately effective against *Leishmania infantum*. Furthermore, structure–activity relationship analysis, supported by quantum calculations and crystallography, revealed meta-halogenation to be more favorable than para, although single halogenation reduced antiparasite effects. Our fragment hybridization approach led to less toxic, more effective compounds than **I**.

## Graphical abstract

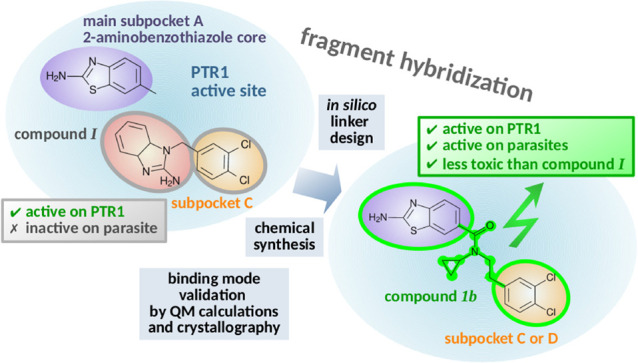

## Introduction

Trypanosomatid protozoans, such as *Trypanosoma brucei* and *Leishmania major*, cause devastating diseases,^1^ which lead to human suffering and high socioeconomic costs in many developing countries. Many existing treatments are insufficiently effective or accessible, and are adversely affected by common problems such as microbial resistance or severe side effects.^2^ Recently, there have been notable advances, including the introduction of eflornithine-nifurtimox therapy, the approval of the oral medication fexinidazole, and the successful phase II/III trials of the oral drug candidate acoziborole for the management of sleeping sickness.^3–5^ Nonetheless, the proactive development of alternative therapeutics remains of high importance.

One approach to designing new antitrypanosomatid drugs is to block the folate pathway of trypanosomatids.^6^ For the *Leishmania* species, this requires inhibiting both dihydrofolate reductase (DHFR) and the trypanosomatid-specific enzyme pteridine reductase 1 (PTR1),^7^ whereas for *T. brucei*, PTR1 (*Tb*PTR1, Figure 1a) was shown to be essential for parasite survival^8^ and therefore its inhibition alone should be sufficient. Anti-PTR1 drug design campaigns reported to date, including those of the NMTrypI (New Medicines for Trypanosomatidic Infections) consortium (https://fp7-nmtrypi.eu/),^9^ have resulted in many hits and leads.^10,11^ These include compounds based on the folate substrate and classical inhibitor scaffolds,^12–16^ diverse compounds from virtual screening of compound libraries,^14,17,18^ and compounds from exploring other scaffolds^19–22^ and from fragment-based drug design (FBDD) approaches.^23,24^

**Figure 1:**
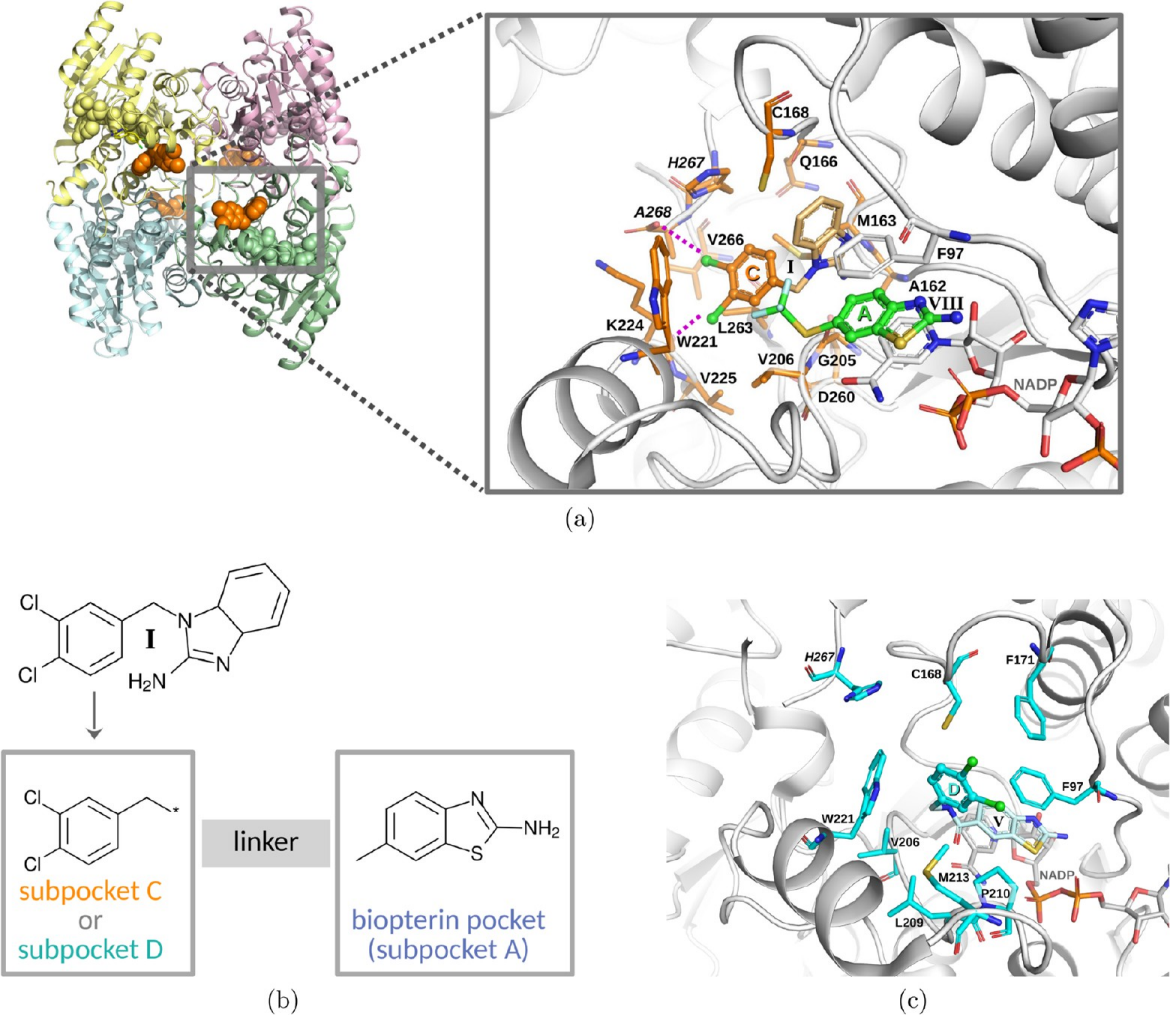
Structure-guided fragment hybridization drug design approach. (a) Crystal structure of the *Tb*PTR1-NADP^+^ homotetramer complex with compound **I** (left, PDB code 3GN2 ^23^) and the magnified view of the active site in the same structure with **I** and its 3,4-dichlorophenyl moiety (in ball-and-stick representation) targeting subpocket C^25^ (the compound and interacting protein residues shown as orange sticks), and 2-aminobenzothiazole derivative **VIII**, targeting subpocket A (biopterin pocket), from the aligned *Tb*PTR1 complex (green sticks, PDB code 6GCQ^22^). Halogen bonds of **I** are shown in magenta. (b) The compound design scheme, involving the decomposition of **I** and linking with the 2-aminobenzothiazole fragment. (c) The *Tb*PTR1 complex with 2-aminobenzothiazole derivative **V** (PDB code 6GEY^22^), with the 3,4-dichlorophenyl moiety (in ball-and-stick representation) in subpocket D^25^ (the compound and interacting protein residues are shown as cyan sticks). The *Tb*PTR1 subpocket C and D residues are also shown in Figures 3a and 4a, respectively, and subpocket A residues are shown in Figure S2 (SI).

In the present study, we focused on developing a series of compounds bearing a 3,4-dichlorophenyl moiety, a key component of potent *Tb*PTR1 inhibitors designed by Mpamhanga et al.,^23^ including, e.g., 1-(3,4-dichlorobenzyl)-1H-benzimidazol-2-amine (**I**, Figure 1a,b, Table 1, see also Figure S1 in SI). These inhibitors displayed up to nanomolar activity against *Tb*PTR1 and about 10-μM activity against the parasite. The compounds were found to bind to nonsubstrate subpockets C and D of *Tb*PTR1 (as defined in ref 25), where the 3,4-dichlorophenyl moiety is positioned in subpocket C (see Figure 1a). In a subsequent study, the 3,4-dichlorophenyl moiety was incorporated into the structures of several derivatives of 2-aminobenzothiazole,^22^ targeting subpocket A (Figure S2 in SI), e.g., in **V** (Figure 1c). The latter was the best *Tb*PTR1 inhibitor among the tested amide-linked 2-aminobenzothiazoles, but was inactive against *T. brucei*. For two of these compounds, **III** and **V** (for 2D structures see Table 1), crystal structures of the *Tb*PTR1 complexes were determined, confirming the positioning of the 3,4-dichlorophenyl moiety in the partly solvent-exposed subpocket D of *Tb*PTR1 (Figure 1c). Furthermore, the 2-aminobenzothiazole derivatives containing the 3,4-dichlorophenyl moiety^22^ displayed linkage-dependent *Tb*PTR1 activities (**II**, **III**, **IV**, and **V**; see Table 1). Linciano et al.^22^ also found that decorating the 3,4-dichlorophenyl-containing compounds with additional substituents affects the on-parasite activity (e.g., **VI**, **VII** vs **V**, see Table 1). Thus, the structural and biochemical data show that both subpockets C and D can be occupied by a 3,4-dichlorophenyl moiety in potent *Tb*PTR1 inhibitors and that their on-target and on-parasite activity may be modulated by altering their scaffolds.

**Table 1:**
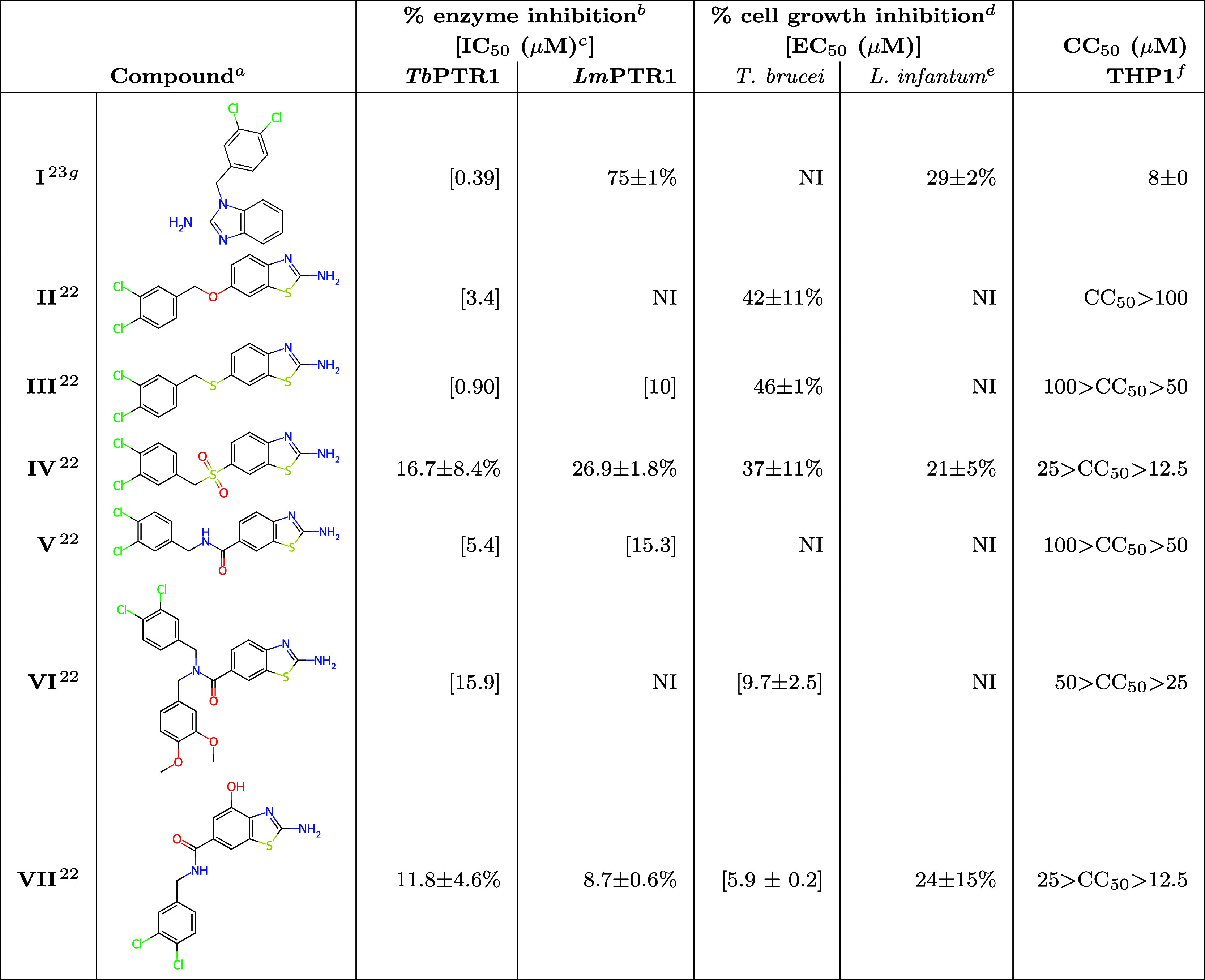
Enzyme and Parasite Inhibition and Toxicity Data for the Reference Compounds^a^

a Original codes for the previously published reference compounds: **I** is 9 from Mpamhanga et al.;^23^ **II**–**VII** correspond to 1d, 2d, 3d, 4d, 4r, 4t from Linciano et al.^22^ Chemical structures of additional reference compounds **VIII**–**X** are in Figure S1 (SI). If not otherwise noted, the experimental data for the reference compounds were obtained with the same methods as for the newly designed and synthesized compounds in Table 2.

b Additional notes: at 50 μM.

c Measurements done in triplicate; standard deviation is within ± 10% of the value, as reported previously.^14,18,21,22^

d At 10 μM.

e *L. infantum* intracellular amastigotes.

f Cytotoxic concentration for THP1 cells.

g All data for **I** were remeasured by the NMTrypI consortium for methodological consistency with the other data; NI, no inhibition.

The reasons for varying levels of on-target and on-parasite compound activity (Table 1) are not clear and might be related to active and inactive transport to parasite cells, intracellular metabolism (which can both result in insufficient intracellular concentrations), or off-target and polypharmacological effects. As shown by the data in Table 1, even small structural changes can lead to significant differences in the activity of 2-aminobenzothiazole derivatives, and the reasons for these differences were not determined previously.^22^ We can note, e.g., that extending the amide-based linker of **V** by substituting the amide nitrogen, which results in compound **VI**, significantly increases anti-*T. brucei* activity. This prompted us to investigate the structure–activity relationship (SAR) associated with targeting subpockets C and D in an FBDD approach to design new 2-aminobenzothiazole derivatives based on the scaffold of **V**.

For **I** and its derivatives, formation of halogen bonds was observed in crystallographic complexes with *Tb*PTR1 (PDB code: 3GN2,^23^ see Figure 1a). Considering the importance of halogen bonds in protein–ligand binding and drug design,^26,27^ we decided to explore the interactions of halogenated compounds with the PTR1 targets. Halogens have unique properties: despite their overall hydrophobicity, they are polarizable and able to form directional halogen bonds with electron donors and acceptors. These unique interactions may at least partly explain why halogen substitutions have in many cases been proven to significantly increase the potency of enzyme inhibitors,^26^ often through increasing residence time.^28^ Halogenated derivatives of **I** were previously tested by Spinks et al.,^24^ and we decided to extend this study for the selected scaffold designed in this work.

Therefore, in this paper, using the SAR of the previously designed 2-aminobenzothiazoles and derivatives of **I**,^22–24^ we adopted a fragment hybridization strategy^29–32^ and redesigned the compound **V** scaffold (Table 1) to target both subpocket A with the 2-aminobenzothiazole fragment and the nonsubstrate *Tb*PTR1 subpockets C and D with the 3,4-dichlorophenyl tail. We also considered *Lm*PTR1 as an additional target to extend and explain the SAR in the context of two-species PTR1 targeting. The adopted workflow is illustrated in Figure 2. With molecular docking, we evaluated the interactions of a series of compounds composed of the 2-aminobenzothiazole core and the 3,4-dichlorophenyl moiety connected by various linkers (Figure 1b). Five compounds were selected for synthesis and tested against PTR1 and parasites, and the SAR for these was analyzed. Furthermore, with the aid of quantum-mechanics (QM) level binding energy calculations, we assessed the effect on *Tb*PTR1 binding energies of different halogen substitutions to the phenyl ring of compounds sharing the same scaffold. The QM calculation results, together with the *Tb*PTR1 activity assays and crystallographic data for the selected synthesized compounds, provide insights into the SAR and an improved understanding of the binding modes of the halogenated compounds.

**Figure 2:**
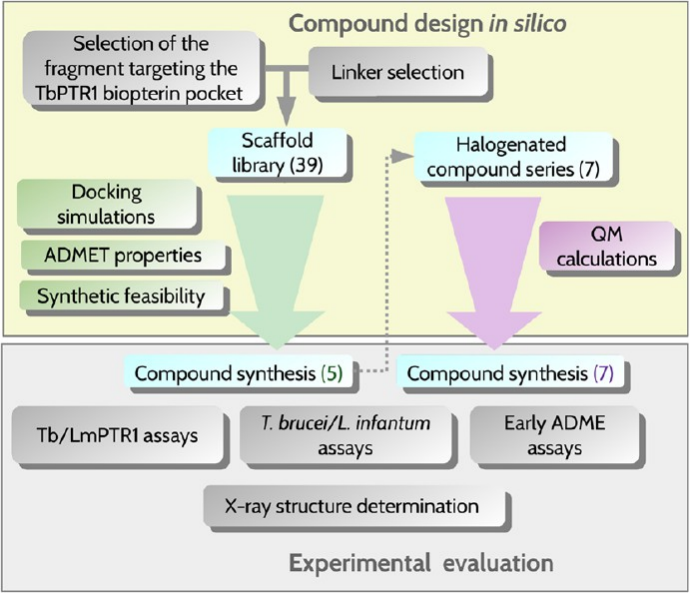
Multidisciplinary FBDD compound design and evaluation workflow. Numbers of compounds computationally evaluated and synthesized are given in parentheses.

## Results and Discussion

### PTR1 Subpockets in *T. brucei* and *L. major* Show Communalities and Differences

For *Tb*PTR1, we evaluated targeting the two subpockets, C and D, adjacent to the main biopterin binding site (Figures 3a and 4a). Subpocket C (Figure 3a) is predominantly hydrophobic, flanked by the indole ring of Trp221, and features polar spots created by the backbone carbonyl oxygen of Trp221, His267, and the Ala268 C-terminus. Subpocket D (Figure 4a), forming an entrance to the pocket, is surrounded by mostly hydrophobic residues. Notably, Trp221 flanks and divides the *Tb*PTR1 pocket into subpockets C and D and adopts a similar conformation in the available crystal structures (see Figure S3a, SI), though some movement of the side chain can be observed depending on the occupancy of subpocket C, as discussed later. All ligands crystallized to date with *Tb*PTR1 with tails occupying subpocket D have a phenyl moiety in this subpocket, and this moiety is mostly in a similar orientation and stabilized by interactions with Trp221 and Phe97 (Figure S3a, SI).

**Figure 3:**
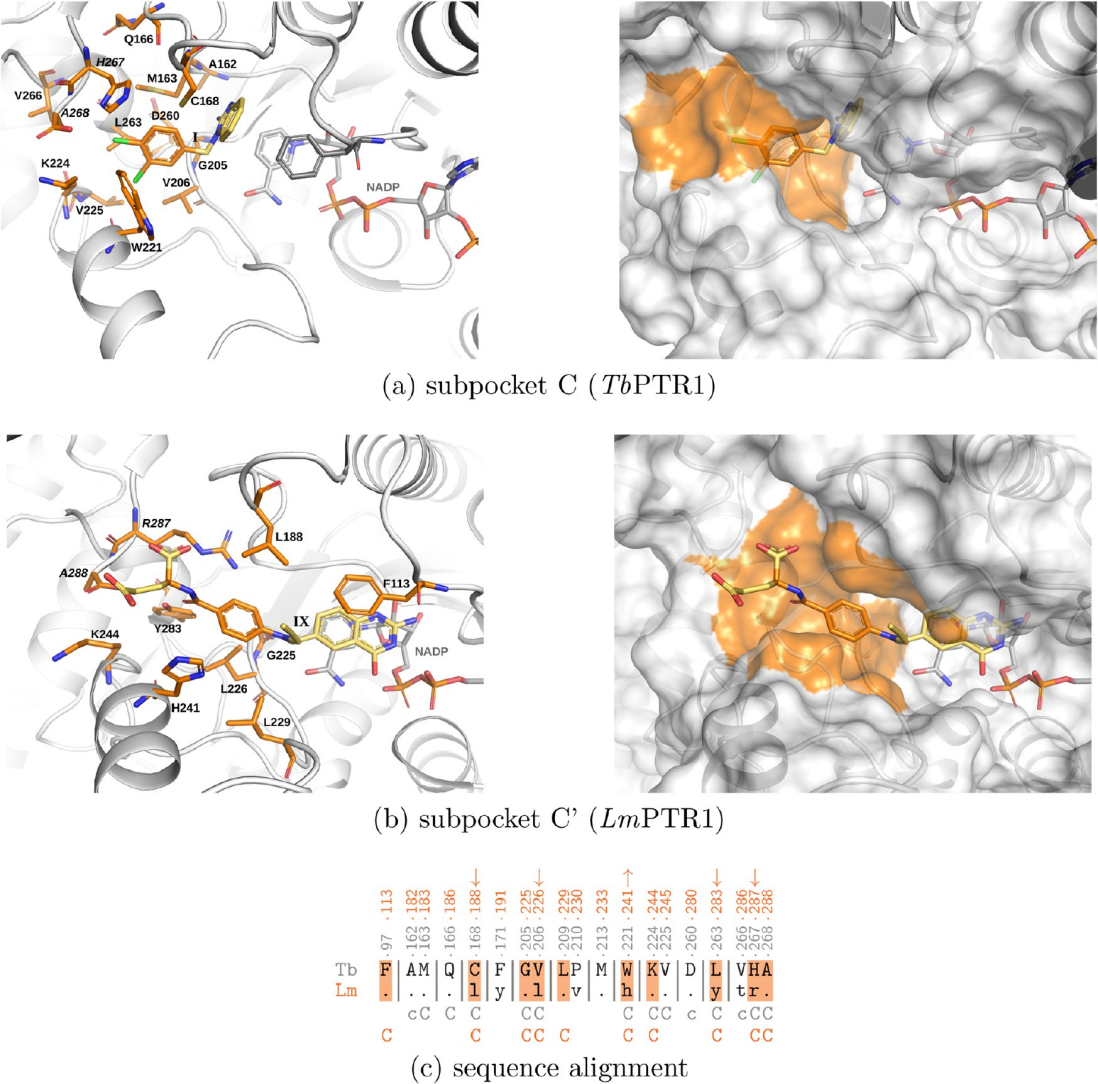
PTR1 subpockets C and C’. (a) Subpocket C in *Tb*PTR1, defined as in ref 25, with reference compound **I** (PDB code 3GN2 ^23^), and (b) subpocket C’ in *Lm*PTR1 with inhibitor **IX** (containing residues within 5.5 Å from the compound tail atoms marked in orange, PDB code 2BFA^33^). The proteins are shown in ribbon representation with residues lining the subpockets in stick representation on the left and surface representation on the right. Orange surfaces indicate the corresponding subpockets. NADP and compounds are shown in stick representation. (c) Sequence alignment of residues of the binding pockets of *Tb*PTR1 and *Lm*PTR1, with subpocket C residues marked. For *Tb*PTR1, the pocket and subpockets are defined as in ref 25, and for *Lm*PTR1, as within 5.5 Å of the ligands’ fragments shown. Sequence numbering for *Tb*PTR1 is shown in gray and for *Lm*PTR1 in orange (subpocket C). The *Lm*PTR1 subpocket C’ residues are shaded in orange. Arrows indicate the significantly (more than 10 g/mol) increased (↑) or decreased (↓) size (molecular weight) of an amino acid in *Tb*PTR1 vs *Lm*PTR1 at the specific position that affects the size of the subpocket.

**Figure 4:**
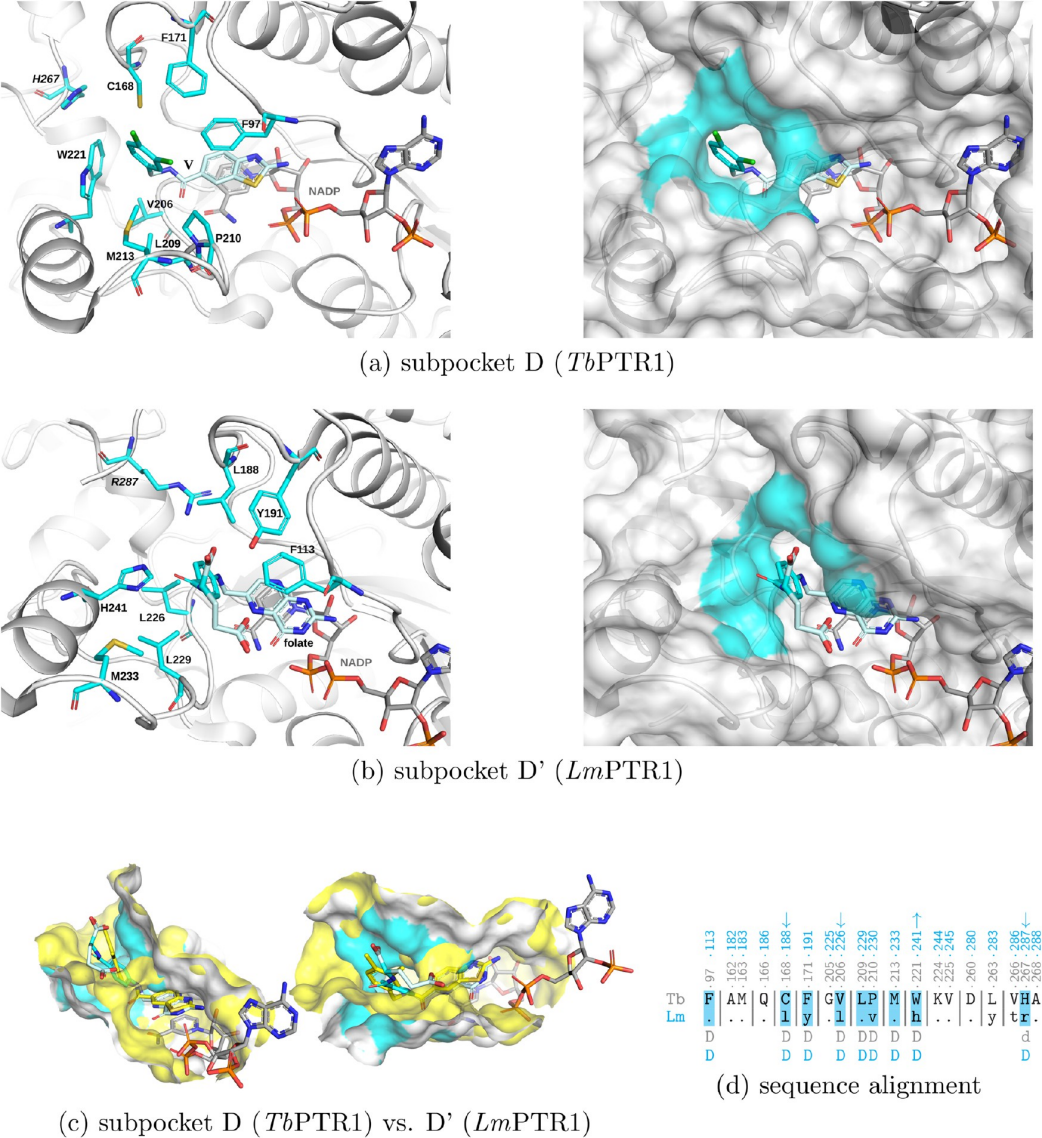
PTR1 subpockets D and D’. (a) Subpocket D in *Tb*PTR1, defined as in ref 25, with reference compound **V** (PDB code 6GEY^22^), and (b) subpocket D’ in *Lm*PTR1 with substrate folate (containing residues within 5.5 Å from the tail fragment colored cyan, PDB code 7PXX^34^). Cyan colored surfaces indicate the corresponding subpockets. (c) Two views on the aligned surfaces of subpockets D (semitransparent yellow) and D’ (solid white and cyan, as in [b]) are shown, with reference ligands to show differences in subpocket shapes. (d) Sequence alignment of residues of the binding pockets of *Tb*PTR1 and *Lm*PTR1, with subpocket D residues marked. For *Tb*PTR1, the pocket and subpockets are defined as in ref 25, and for *Lm*PTR1, as within 5.5–Å of the ligands’ fragments shown. Sequence numbering for *Tb*PTR1 is shown in gray and for *Lm*PTR1 in cyan. The *Lm*PTR1 subpocket D’ residues are shaded in cyan. The meaning of the arrows is explained in Figure 3.

The pocket in *Lm*PTR1 is more open and solvent-exposed than that of *Tb*PTR1.^25^ Thus, subpocket D’, corresponding to subpocket D of *Tb*PTR1, forms a wider entrance to the pocket than in *Tb*PTR1 (Figure 4b,c). In contrast to *Tb*PTR1, *Lm*PTR1’s subpocket D’ does not offer favorable hydrophobic contacts from both sides to ligand moieties such as phenyl, which extend into subpocket D/D’ in ligands like folate (compare Figure S3a,b, SI). It is worth noting that in crystal structures, His241 is much more conformationally variable than the corresponding residue in *Tb*PTR1, Trp221 (Figure S3, SI). It is thus a potentially less stable interaction partner for moieties bound in subpocket D’ or the biopterin pocket (see Figure S3, SI).

In *Lm*PTR1, there is also a half-open subpocket, denoted here as subpocket C’, between Leu188 and His241, corresponding to subpocket C of *Tb*PTR1, but significantly shallower due to the presence of larger residues than in *Tb*PTR1: Leu188 instead of Cys, Leu226 instead of Val, Tyr283 instead of Leu, and Arg287 instead of His (see Figure 3), and more exposed than in *Tb*PTR1, mostly due to the exchange of Trp221 to His241. The subpocket C’ in *Lm*PTR1 is amphipathic. Partly hydrophobic regions are due to the neighboring Leu188, Leu226, and hydrophobic portions of the His241, Lys244, and Arg287 side chains (Figure 3). Hydrophilic spots appear due to the neighboring side chains of Lys244, Tyr283, Arg287, and the C-terminus of Ala288. The crystallographic data (PDB code 2BFA,^33^ Figure 3b) show that subpocket C’ can be occupied by an inhibitor: it binds the para-aminobenzoic acid and glutamyl moieties of compound **IX** (CB3717, for chemical structure see Figure S1, SI).

Thus, inhibitor moieties designed for targeting subpockets C and D of *Tb*PTR1 could also target subpockets C’ and D’ in *Lm*PTR1, respectively. Further, for flexible ligands, the tails might also target subpocket C in *Tb*PTR1 and D’ in *Lm*PTR1, and vice versa, which could increase chances of finding pan-parasite PTR1 inhibitors. For example, the inhibitors with more polar tails, compatible with *Lm*PTR1 subpocket C’, could bind in solvent-exposed subpocket D of *Tb*PTR1. However, only the subpocket C of *Tb*PTR1 and the (corresponding) subpocket C’ of *Lm*PTR1 have the potential to form halogen bonds with halogenated ligands through the backbone carbonyl oxygen of Trp221 in *Tb*PTR1 ^23^ and His241 in *Lm*PTR1, which are similarly located (see Figure S4, SI). The latter halogen bond has not been observed in the crystal structures of the *Lm*PTR1 complexes so far. Therefore, targeting subpocket C or D of *Tb*PTR1 also offers the possibility to simultaneously target *Lm*PTR1, in particular with less hydrophobic moieties, although the expected interaction patterns would likely differ.

### Construction of the Virtual Compound Library of Compounds with Designed Linkers

A library of compounds containing the two fragments—2-aminobenzothiazole and 3,4-dichlorophenyl—connected by a series of linkers was designed computationally (Figure 1b). Most of the linkers were one of the types shown in Figure 5. First, following one of the series of the previously synthesized 2-aminobenzothiazoles,^22^ we focused on amide-based linkers, because these offer the possibility to easily ‘branch’ the compound with additional substituents (see red arrow in Figure 5). We further considered amine-based linkers, which also offer the possibility of ‘branching’ the compounds (tertiary amines, see red arrow in Figure 5), since molecular interaction fields calculated previously^25^ suggested favorable spots for hydrogen bond donors (but not for acceptors) in the center of the *Tb*PTR1 pocket, where the linker amide bond would be likely located.

**Figure 5:**
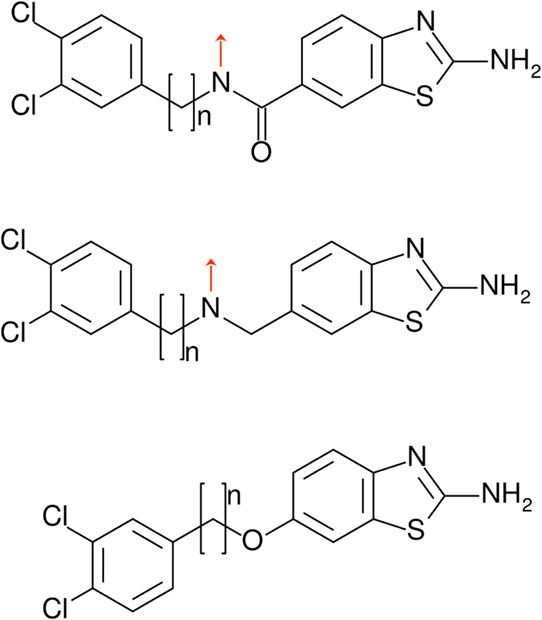
Designed series of compounds with three types of linkers connecting the 3,4-dichlorophenyl and 2-aminobenzothiazole fragments. Red arrows indicate possible substitution points.

Most amine or amide-based linkers in the constructed library were N-substituted (Figure S5, SI). If the 3,4-dichlorophenyl moiety of the designed compounds bound in subpocket C (Figure 3a), which we aimed to prioritize, the N-substituent would be positioned in subpocket D (Figure 4a). Due to the hydrophobicity and partial solvent exposure of subpocket D, the N-substituents tested in silico were largely hydrophobic, and some additionally had a hydrophilic moiety.

The next important question was which length of the linker would ensure optimal interactions of both linked fragments with *Tb*PTR1 in subpocket C or D. The 3,4-dichlorophenyl moiety was already used as a substituent in the previously published 2-aminobenzothiazole series^22^ (e.g., compounds **II**, **III**, and **V** in Table 1). Overall, the crystallographic data show that the substituents connected by three-atom or shorter linkers result in positioning of the 3,4-dichlorophenyl moiety in subpocket D of the *Tb*PTR1 binding site, so that the moiety is exposed to solvent (e.g., see the crystallographic complex of **V** and *Tb*PTR1 in Figure 4a). One hypothesis is that, with shorter linkers, the 3,4-dichlorophenyl tail does not reach the *Tb*PTR1 subpocket C to form favorable halogen bonds, and therefore instead occupies subpocket D. Indeed, in docking simulations of **V**, for the top-ranked pose (by docking score, Figure 6a), in which the 3,4-dichlorophenyl tail is positioned in subpocket C, no halogen bonds are formed, and typical hydrogen bonds of the 2-aminobenzothiazole core are not formed. In contrast, the second-best pose (Figure 6b) has the 3,4-dichlorophenyl tail in subpocket D and corresponds to the binding mode in the crystallographic complex with *Tb*PTR1 (Figure 4a). So far, none of the 2-aminobenzothiazole derivatives crystallized with *Tb*PTR1 have extended into subpocket C, except compound **X**,^22^ which occupies both subpockets C and D with a branched substituent (Figure S6, chemical structure in Figure S1, SI). Therefore, to evaluate the possibility of targeting subpocket C, we tested four-atom-long linkers, as present in the amide-based scaffold of **1a** (Table 2). For **1a**, in the top-ranked pose from docking simulations (Figure 6c), one halogen bond forms, together with the expected hydrogen bonding pattern of 2-aminobenzothiazole. However, the tail of **1a** occupies subpocket D in the second-best pose (Figure 6d), suggesting that such a binding mode is also possible.

**Figure 6:**
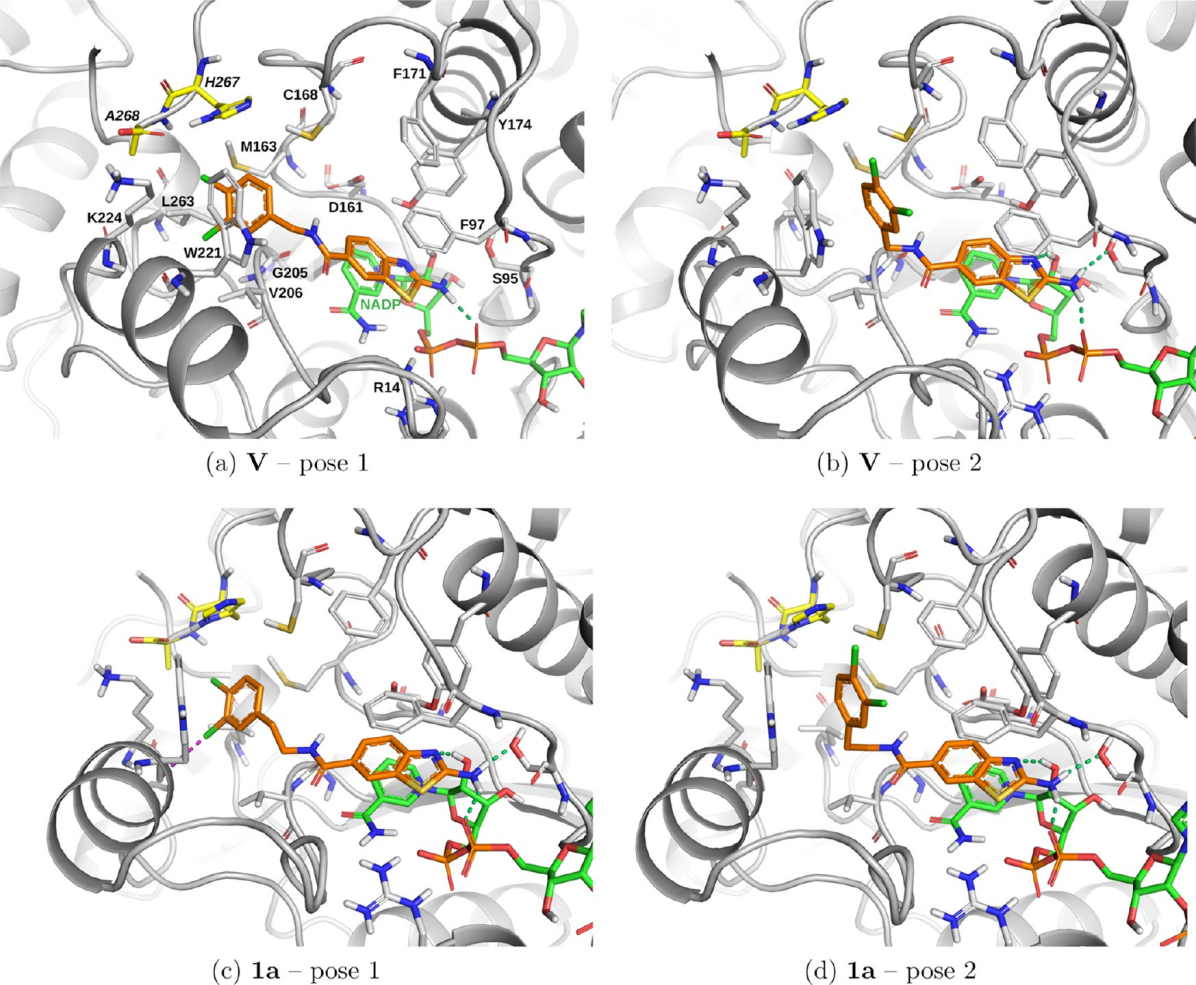
Top two docking poses of **V** and **1a** in *Tb*PTR1. The 3,4-dichlorophenyl moiety is located in subpocket C (a and c, respectively) or subpocket D (b and d, respectively). Hydrogen bonds and halogen bonds are indicated by green and magenta dashed lines, respectively. Docking scores are presented in Table S2 (SI). The compounds were docked into a crystal structure of *TbP*TR1 with the PDB ID 9QDK.

**Table 2:**
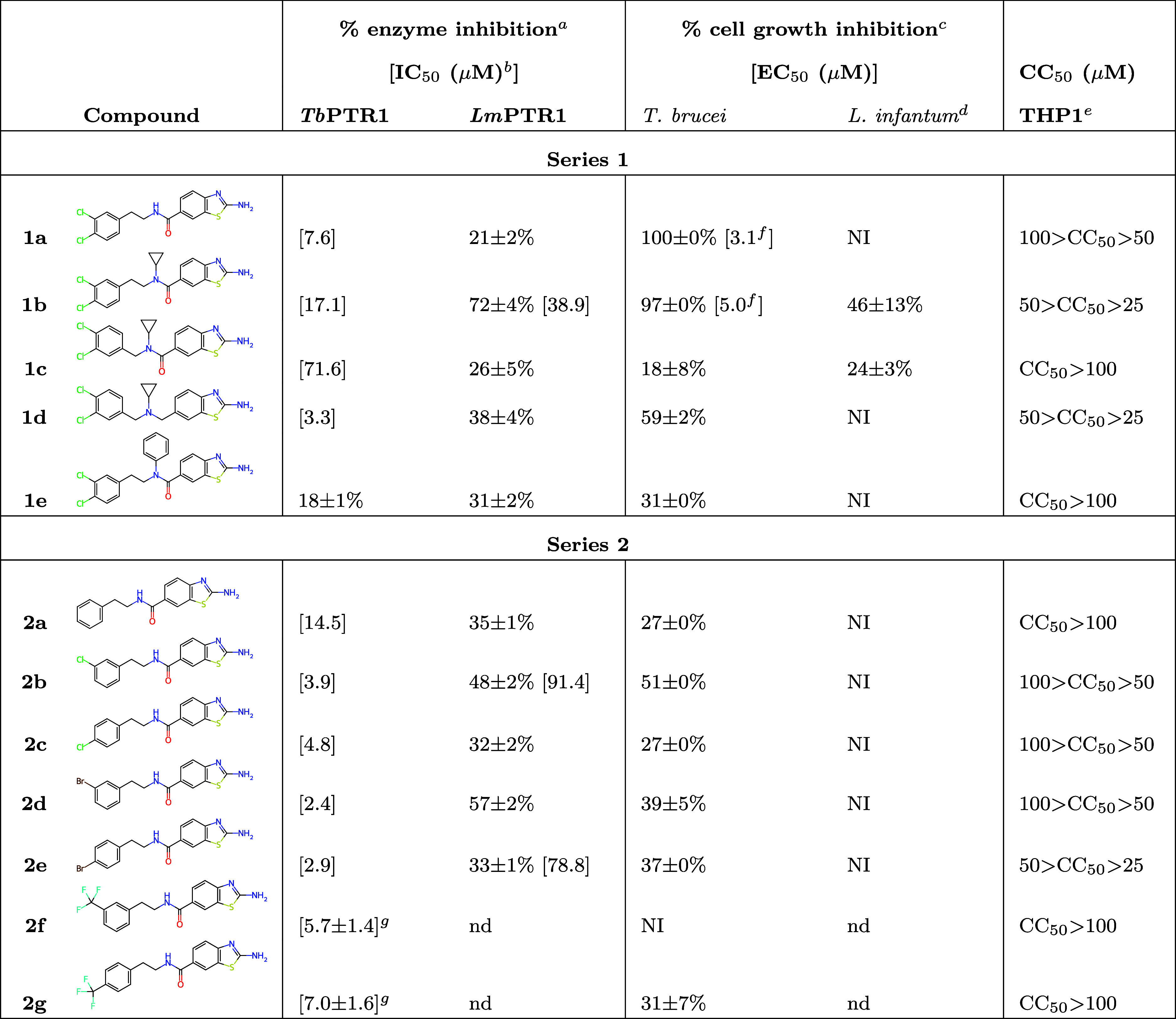
Measured Enzyme and Parasite Inhibitory Activity and Toxicity

a At 50 μM.

b If not otherwise noted, measurements were performed in triplicate, and the standard deviation is within ± 10% of the value, as reported previously.^14,18,21,22^

c At 10 μM.

d *L. infantum* intracellular amastigotes.

e Cytotoxic concentration for THP1 cells.

f 95% confidence interval for EC_50_: **1a**: 2.83–3.37 μM, **1b**: 4.45–5.88 μM.

g Direct method of activity measurement, experiment performed in duplicate (in contrast to the other IC_50_ results); NI, no inhibition; nd, no data.

On one hand, there may be an unfavorable entropic contribution to binding free energy for the compounds containing longer, more rotatable linkers if their flexibility is restrained upon binding. On the other hand, such linkers are more adaptable, which may be advantageous for targeting multiple PTR1 variants (and potentially also parasite DHFR). There is, however, a risk of binding to off-targets. Therefore, we also tried to make the initial scaffolds more conformationally restrained by substituting the scaffolds with the amide or amine nitrogen. All scaffolds tested in silico are shown in Figure S5 (SI). Preliminary computational ADMET predictions indicated no significant liabilities for these compounds (see more details and additional data in Table S1, SI).

### Criteria for Selection of Compounds for Chemical Synthesis

The compound library was evaluated via docking simulations. We primarily focused on *Tb*PTR1 as the main target, since our FBDD approach was aimed mainly at this PTR1 variant, and 2-aminobenzothiazoles had already been shown to be overall less active against the other important trypanosomatid target *Lm*PTR1.^22^ Favorable binding to *Tb*PTR1 was thus one of the main criteria for compound selection. Nevertheless, the binding modes of compounds in *Lm*PTR1 were also analyzed, and compound activities were measured against *Lm*PTR1 to evaluate the multi-PTR1-targeting inhibitory potential.

Compound selection was based on a set of criteria in addition to favorable docking scores, since docking scores are known not to correlate well with binding free energies.^35^ Four compounds were selected for synthesis based on the combined consideration of the following criteria: (i) binding mode in *Tb*PTR1 reproducing the 2-aminobenzothiazole binding mode, as observed in the previous work^22^ (e.g., see Figure 4a); and with the 3,4-dichlorophenyl moiety located in either subpocket C or in subpocket D; (ii) better or comparable top docking score than the reference compound **V**; (iii) acceptable predicted in silico ADMET properties; (iv) synthetic feasibility and availability of reagents, which was evaluated by expert opinion. The docking scores for selected poses (sorted by docking score) of the reference compounds and the five compounds selected for synthesis (series 1, compounds **1a**–**1e**) are shown in Tables S2 and S3 (SI).

### Docking Poses in *Tb*PTR1 Show Occupation of Both Subpockets C and D by Designed Compounds

The top docking poses of the first selected scaffold, **1a**, show that the phenyl tail occupies either subpocket C or subpocket D of *Tb*PTR1 (Figure 6c,d). In the pose with the phenyl tail of **1a** in subpocket C, one halogen bond is formed between the *meta*-Cl of the 3,4-dichlorophenyl moiety and the backbone carbonyl oxygen of Trp221 (Figure 6c). In the pose with the phenyl tail occupying subpocket D, the placement of the tail is similar to that of **V** (Figure 6b,d, respectively).

The 3,4-dichlorophenyl and 2-aminobenzothiazole moieties of both **1b** and **1c**, which have N-cyclopropyl substituents, can in principle adopt two configurations: with the N-cyclopropyl moiety on the same or on the opposite side of the peptide bond as 2-aminobenzothiazole, referred to here as *cis* and *trans*, respectively. Both configurations, *cis* and *trans*, were considered in our analyses as possible binding modes. In the docking results, both configurations of **1b** and **1c** show favorable interactions with *Tb*PTR1 and appear as the first two docking poses in the score-based ranking (Figure 7a–d, Table S2 in the SI). For the *trans* configuration of **1b** (pose 1, Figure 7a), one halogen bond is formed by the 3,4-dichlorophenyl moiety—as for **I**, it is between the *meta*-Cl and the backbone carbonyl oxygen of Trp221. In the *cis* configuration (pose 2, Figure 7b), the tail of **1b** occupies subpocket D. The *trans* and *cis* binding poses show comparable contributions to the Glide docking score (van der Waals: −45.0 vs −45.2 kcal/mol; electrostatic: −6.5 vs −8.1 kcal/mol, respectively, Table S2, SI). The internal energy is, however, significantly more favorable for the *trans* than the *cis* configuration (6.6 vs 13.7 kcal/mol, Table S2, SI). In the *trans* pose (pose 1, Figure 7a), the N-cyclopropyl is in contact with the side chains of Met213 and Trp221, while in the *cis* configuration, it contacts the side chain of Cys168 (pose 2, Figure 7b).

**Figure 7:**
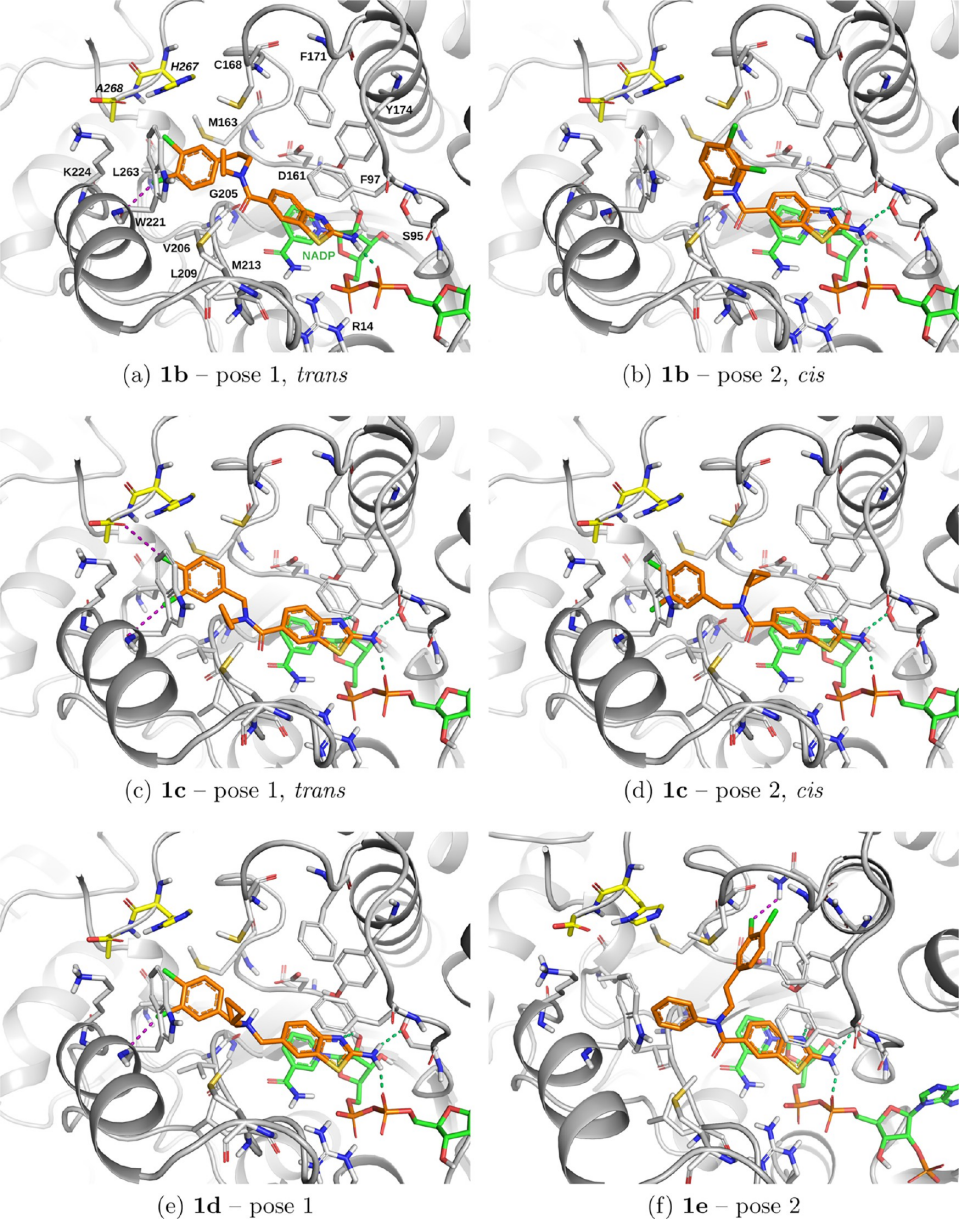
Docking poses to *Tb*PTR1 for the amine or amide-substituted compounds selected for synthesis: **1b** (a,b), **1c** (c,d), **1d** (e), and **1e** (f). The Glide SP top poses sorted by docking score are shown. Hydrogen bonds and halogen bonds are indicated by green and magenta dashed lines, respectively. Docking scores are given in Table S2 (SI). The compounds were docked into a crystal structure of *TbP*TR1 with PDB ID 9QDK.

**1c** has the same N-substituent as **1b**, but a shorter three-atom-long linker. The *trans* configuration of **1c**, similarly to **1b**, is the favored one in terms of both Glide SP docking score and Emodel score, and reproduces the two expected halogen bonds of the tail (Figure 7c), whereas these halogen bonds are absent for the *cis* configuration, in which the 3,4-dichlorophenyl is also in subpocket C (Figure 7d). **1c** has better van der Waals energy (−50.2 vs −47.0 kcal/mol) and better internal energy (11.6 vs 16.8 kcal/mol) in the *trans* than *cis* configuration, whereas the electrostatic component is comparable. In the *trans* pose, the N-cyclopropyl is in contact with the side chains of Leu209, Met213, and Trp221 (pose 1, Figure 7c), while in the *cis* configuration, it contacts the side chain of Phe97 (pose 2, Figure 7d).

The top pose (in terms of both docking score and Emodel) of **1d**, the only selected compound with an amine-based three-atom-long linker, was predicted to fit reasonably well in *Tb*PTR1, with the tail in subpocket C and one halogen bond formed between the *meta*-Cl and the backbone carbonyl oxygen of Trp221 (Figure 7e). The N-cyclopropyl substituent is in the vicinity of Cys168, Trp221, and Met213, with all of which it can potentially form closer contacts if some flexibility of the amine-based linker is assumed.

The last selected compound, **1e**, has a flipped position of the 2-aminobenzothiazole ring in the top-scoring pose (not shown), so it was discarded according to the selection criteria, whereas in the second pose (Figure 7f), the 3,4-dichlorophenyl tail is positioned in subpocket B,^25^ forming a halogen bond with the side chain amide nitrogen of Asn175. Although this compound did not fully satisfy the selection criteria, it was selected for synthesis to support the elucidation of the SAR.

### Docking Poses in *Lm*PTR1 Show Specific Linker Length Preference

In docking to *Lm*PTR1, all compounds selected for synthesis except **1e** show standard binding modes of 2-aminobenzothiazole (Figure 8) with hydrogen bonds to the phosphate of the NADP cofactor and one hydrogen bond between the 2-aminobenzothiazole nitrogen (as an acceptor) and the hydroxyl group of the cofactor ribose. For **1b**, the hydrogen bond to Ser111 is not formed (Figure 8b,c), while for **1e**, the 2-aminobenzothiazole ring is flipped (Figure 8f). The tails in all the top poses are in extended configurations, targeting subpocket C’ of *Lm*PTR1, similarly to the binding modes for *Tb*PTR1 with the tail in subpocket C (Figure 7a,c,d,e).

**Figure 8:**
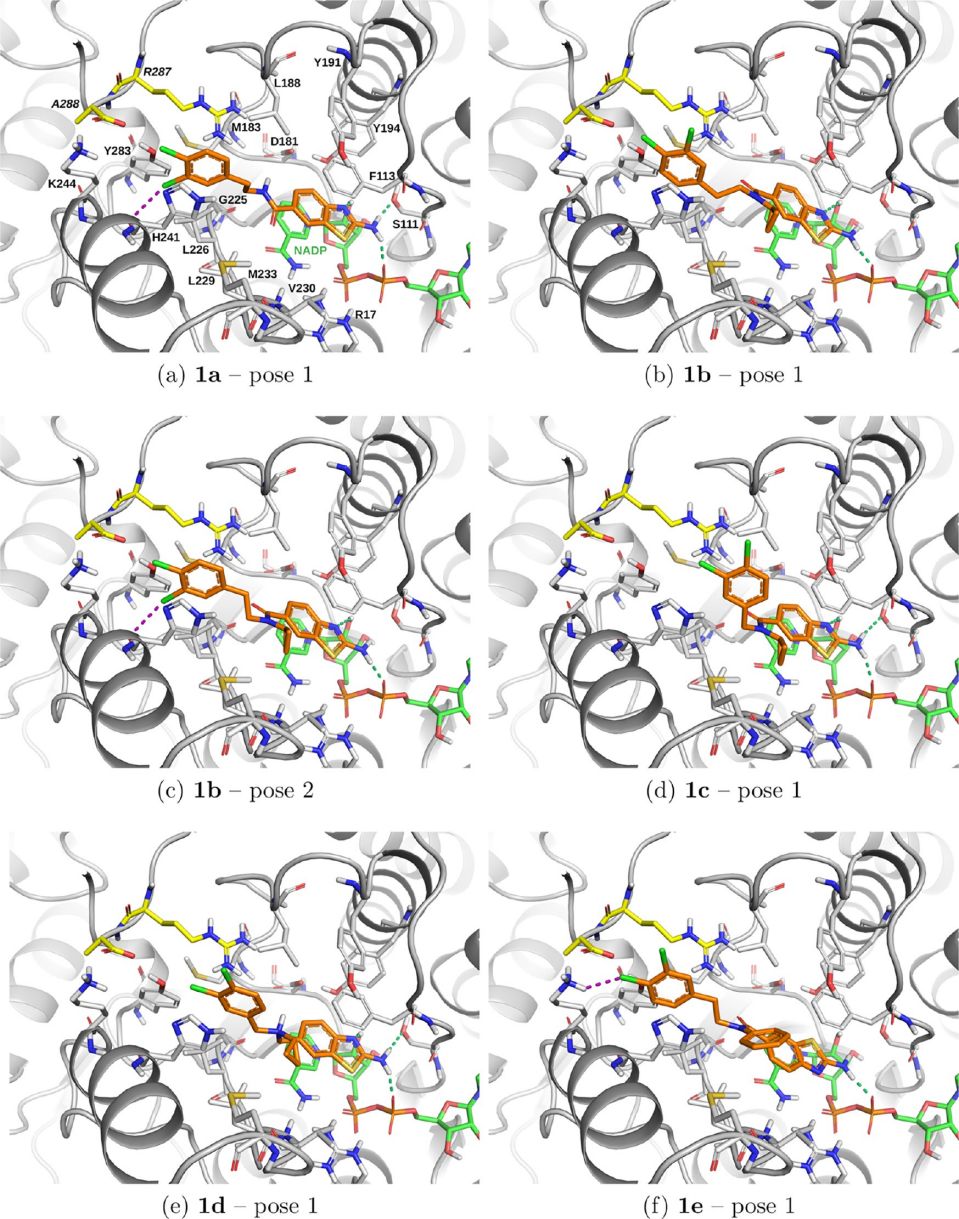
Docking poses to *Lm*PTR1. The top poses according to the docking score are shown for **1a** (a) and the amine or amide-substituted compounds **1b**–**1e** selected for synthesis (panels b–f, respectively). Hydrogen bonds and halogen bonds are indicated by green and magenta dashed lines, respectively. Docking scores are given in Table S3 (SI). The compounds were docked into a crystal structure of *Lm*PTR1 with the PDB ID 1E92.

All the top poses of the N-substituted compounds display a *cis* configuration (of the N-substituent vs the 2-aminobenzothiazole core). For **1b**, **1c**, and **1d** (Figure 8b–e), the N-cyclopropyl substituent forms favorable van der Waals interactions with the surrounding hydrophobic residues, Phe113 and Leu229, and potentially, with Val230, if the latter side chain is rotated. The *N*-phenyl substituent of **1e** (Figure 8f) forms contacts with Met233 and Phe113, but it does not form stacking interactions and is substantially solvent-exposed, which may be unfavorable for binding.

For **1a** and **1b**, one of the chlorine atoms in the tail is a halogen bond donor to the backbone carbonyl oxygen of His241 (Figure 8a,c), and for **1e**, it is a halogen bond acceptor from the side chain of Lys244 (Figure 8f). For these compounds, with four-atom-long linkers, the phenyl tail is also involved in parallel stacking interactions with the imidazole ring of His241. In contrast, in the shorter scaffolds of **1c** and **1d**, the 3,4-dichlorophenyl tail does not form stacking interactions (Figure 8d,e).

Thus, based on inspection of the docking data, the four-atom-long linkers seem to be more optimal for targeting *Lm*PTR1, due to the stacking interactions with His241, and the N-cyclopropyl substituent of **1b**, **1c**, and **1d** makes stabilizing interactions with both *Lm*PTR1 and *Tb*PTR1. Therefore, the docking poses indicate that some of the selected compounds may have potential for targeting two-species PTR1.

### Synthesis of Designed Compounds

The benzothiazoles of series 1 and 2 (discussed later) were synthesized as follows. The 6-amide derivatives **1a**, **1b**, **1c**, **1e**, **2a**, **2b**, **2c**, **2d**, **2e**, **2f**, and **2g** were obtained by coupling commercially available 2-aminobenzothiazole-6-carboxylic acid with the appropriate amines (**3a**–**3k**) under standard conditions (using EDC and HOBt in DMF at room temperature, overnight) (Scheme 1).

**Scheme 1:**
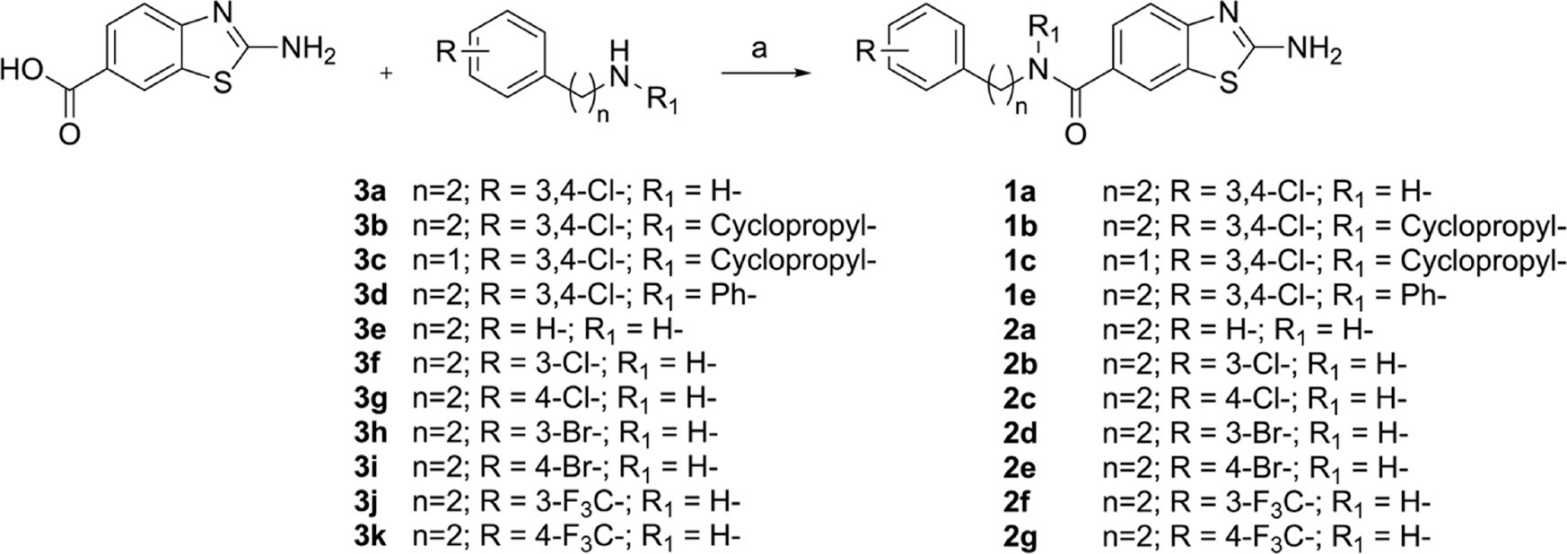
Reagents and Conditions: (a) 2-aminobenzothiazole-6-carboxylic Acid (1 Equiv), Amine (1 Equiv), EDC·HCl (1 Equiv), HOBt (1 Equiv), Triethylamine (1 Equiv), Anh. DMF, r.t., Overnight

Only the 6-amine derivative, **1d**, was prepared by reducing **1c** with LiAlH_4_ in anhydrous THF at −20 °C for 2 h (Scheme 2).

**Scheme 2:**
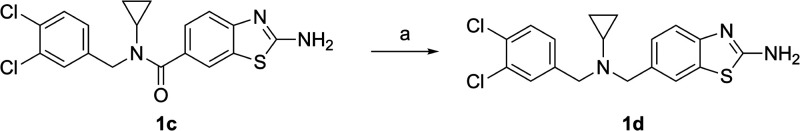
Reagents and Conditions: (a) LiAlH_4_ 1M in THF (1.2 Equiv), Anh. THF, −20°C, 2 h

The reference compound **I** has been synthesized according to the literature.^23^ Amines **3a**, **3e–** **3k** were commercially available and used without purification, whereas **3b–** **3d** were prepared as shown in Scheme 3. Synthesis of N-cyclopropyl-3,4-dichlorophenethylamine (**3b**) involved hydrolysis of 3,4-phenylacetonitrile to the corresponding carboxylic acid (**4**) in refluxing concentrated HCl for 6 h. Subsequent coupling of **4** with cyclopropylamine (under the same standard conditions) afforded the amide **5**, which was then reduced to amine **3b** with LiAlH_4_ in anhydrous THF at reflux for 3 h. N-cyclopropylbenzylamine (**3c**) was obtained via an S_N_2 reaction between 3,4-dichlorobenzyl bromide and cyclopropylamine, using K_2_CO_3_ as the base in DMF at room temperature overnight. Lastly, *N*-phenyl-3′,4’-dichlorophenethylamine (**3d**) was prepared by reacting 3,4-dichlorophenethylamine with phenylboronic acid in the presence of copper­(II) acetate as a catalyst (Scheme 3).

**Scheme 3:**
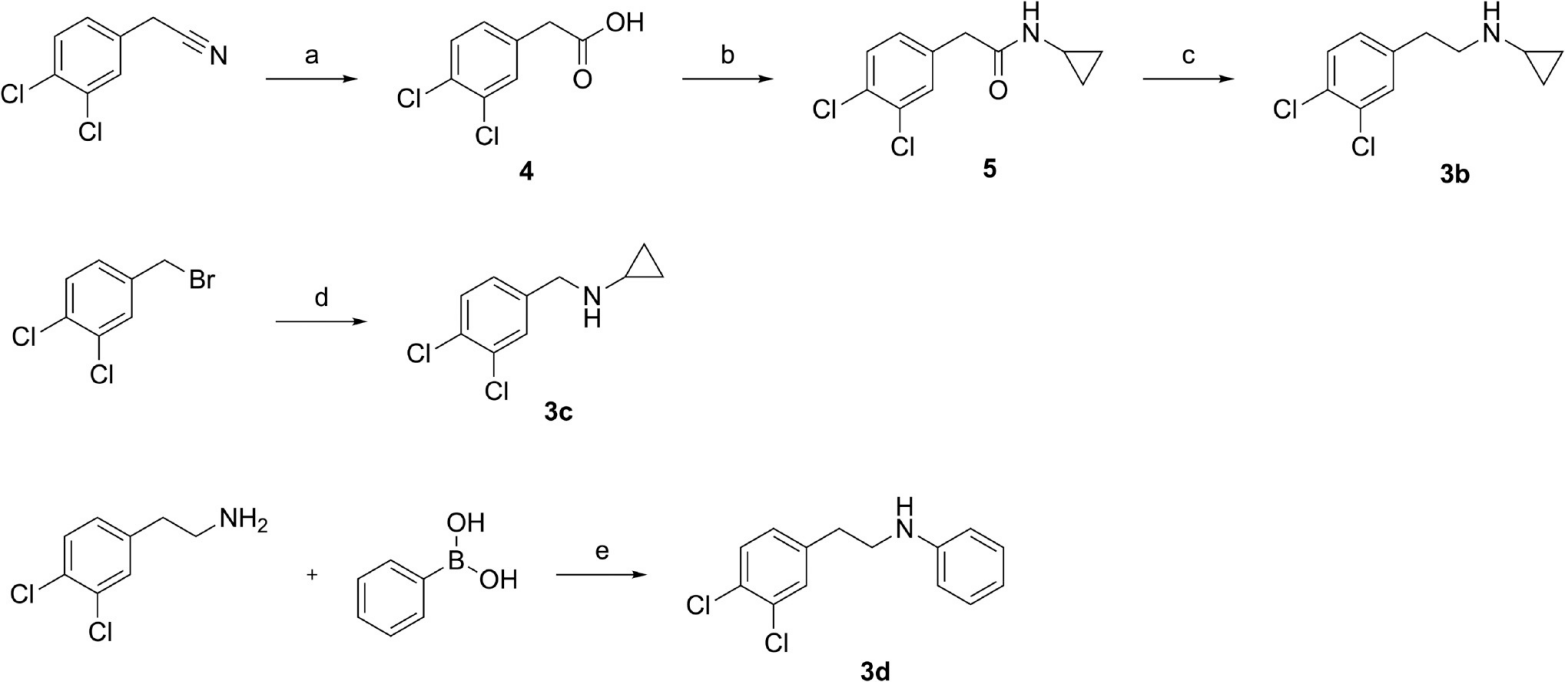
Reagents and Conditions: (a) 37% HCl Aq., Water, Refl., 6 h; (b) Cyclopropylamine (1 Equiv), EDC·HCl (1 Equiv), HOBt (1 Equiv), Triethylamine (1 Equiv), Anh. DMF, r.t., Overnight; (c) LiAlH_4_ 1M in THF (1.2 equiv), Anh. THF, 0°C to Refl., 3 h; (d) Cyclopropylamine (2 Equiv), K_2_CO_3_ (2.5 Equiv), DMF, r.t., Overnight; (e) CuAcO (1% mol), DCM, r.t. 1 h

### The Activities and Toxicities for Series-1 Compounds Pinpoint Lead Scaffolds for Further Antitrypanosomatid Drug Design

The inhibitory activities against *Tb*PTR1, *Lm*PTR1, *T. brucei*, and *L. infantum*, as well as the cytotoxic concentration for THP-1 cells, were measured for the differently linked compounds (Table 2, series 1). The best compounds have similar activity against *Tb*PTR1 as the previously reported amide-linked 2-aminobenzothiazoles:^22^ the best compound of that series was **V** with an IC_50_ of 5.7 μM (Table 1). With the exception of **1e** and **1c** (18% at the tested concentration and IC_50_ = 71.6 μM, respectively), the tested compounds have low micromolar activities against *Tb*PTR1 (Table 2), with IC_50_ values of 3.3 μM for **1d**, 7.6 μM for **1a**, and 17.1 μM for **1b**.

As regards toxicity, the compounds showed a comparable toxicity profile to the previously reported 2-aminobenzothiazole derivatives.^22^ Notably, even the compounds with higher toxicity to THP-1 cells (**1b** and **1d**, CC_50_ 25–50 μM, Table 2) are improved over the parent compound **I** (CC_50_ 8 ± 0 μM, Table 1). Early ADMET profiles (Table S4 in SI) additionally show that compounds **1a**–**1d** significantly inhibit CYPs 2C19, 2D6, and 3A4, and **1b**–**1d** display a significant level of mitotoxicity.

Among the five tested compounds, three show >50% inhibition of *T. brucei* activity. **1d**, with the best IC_50_ for *Tb*PTR1, is moderately active on *T. brucei* (59%). **1a** is the best *T. brucei* inhibitor, showing an EC_50_ of 3.1 μM and a selectivity index toward human THP-1 cells over 16 (Table 2). In addition, **1b** has an EC_50_ of 5.0 μM. However, considering the CC_50_ of 25–50 μM, it is particularly toxic for human cells (Selectivity Index toward THP-1 cells is within the range 5−10). Despite that, **1b** is an interesting scaffold, since, besides good antiparasitic activity against *T. brucei*, it shows activity against *Lm*PTR1 (IC_50_ = 39 μM) together with a moderate antiparasitic activity toward *L. infantum* amastigotes (46% at 10 μM, Table 2). The latter property is unusual for 2-aminobenzothiazole derivatives.^22^ The very low selectivity index of **1b** precludes further evaluation, but its scaffold, containing an N-cyclopropyl substituent at the amide bond, represents a promising starting point for the further development of derivatives targeting *L. infantum*.

It is worth noting that the **1b** scaffold is shared with **1c**, which, unlike the other compounds, also shows some activity against *L. infantum* (24%). However, **1c** has distinctly lower activity against *Lm*PTR1 than **1b** (72 and 26%, respectively). Overall, this suggests that the unusual activities of the N-cyclopropyl-substituted compounds against *L. infantum* parasites may be due to mechanisms other than targeting PTR1, which could be related to binding other critical intracellular targets or facilitating transport to parasite cells. When evaluating the antiparasite activities against *Leishmania*, it must also be remembered that, overall, thiadiazoles and 2-aminobenzothiazoles do not effectively target DHFR,^21,22^ which is required for inhibitors to exert their effect against these trypanosomatids.^7^

### QM Evaluation of the *Tb*PTR1 Subpocket C Reveals Sensitivity of Binding Energies to Positions of Halogen Phenyl Substituents

In subpocket C, halogens on tail phenyls may form halogen bonds (Figure 1), which are poorly described by classical molecular mechanics force fields. Therefore, we investigated the effects of halogen substitutions in the 3,4-dichlorophenyl moiety, positioned as in **I** in the *Tb*PTR1 pocket, on its binding energy to *Tb*PTR1 using the computationally efficient QM-based model of ligand–protein interaction energy.^36,37^ This nonempirical QM model consists of two long-range interaction energy terms—multipole electrostatic energy (*E*_EL,MTP_^(10)^) and approximate dispersion term *D*_as_ ^38,39^ (MED = *E*_EL,MTP_^(10)^ + *D*_as_). In contrast to classical docking approaches, this model is able to capture the electronic nature of halogen bonds at a computational cost as low as that of empirical scoring functions.^36,37^

In the MED model applied here, the binding energy of the selected ligand moiety (or compound) is approximated as the sum of the interaction energies of each pocket residue with the moiety. The MED model has been proven to correlate well with inhibitory activities in a number of protein–ligand complexes (as summarized in ref 37), including the binding of a *Tb*PTR1 ligand series with varying substituents bound in subpocket D.^40^

We assumed that the binding mode of **I** is well-defined, and that substitutions at positions 3 and 4 of the phenyl ring will not significantly change the phenyl moiety position in the *Tb*PTR1 subpocket C. In addition to the MED energy terms, we also calculated the short-range exchange repulsion energy *E*_EX_^(10)^ to obtain more insight into possible steric clashes. We calculated the interactions of phenyl rings that were either unsubstituted or substituted at position 3 (termed *meta*/m) and/or 4 (*para*/p) with F, Cl, Br, CF_3_, or CH_3_ (Figure S7, SI). The main calculation results are shown in Figure 9 and Table S5 in the SI.

**Figure 9:**
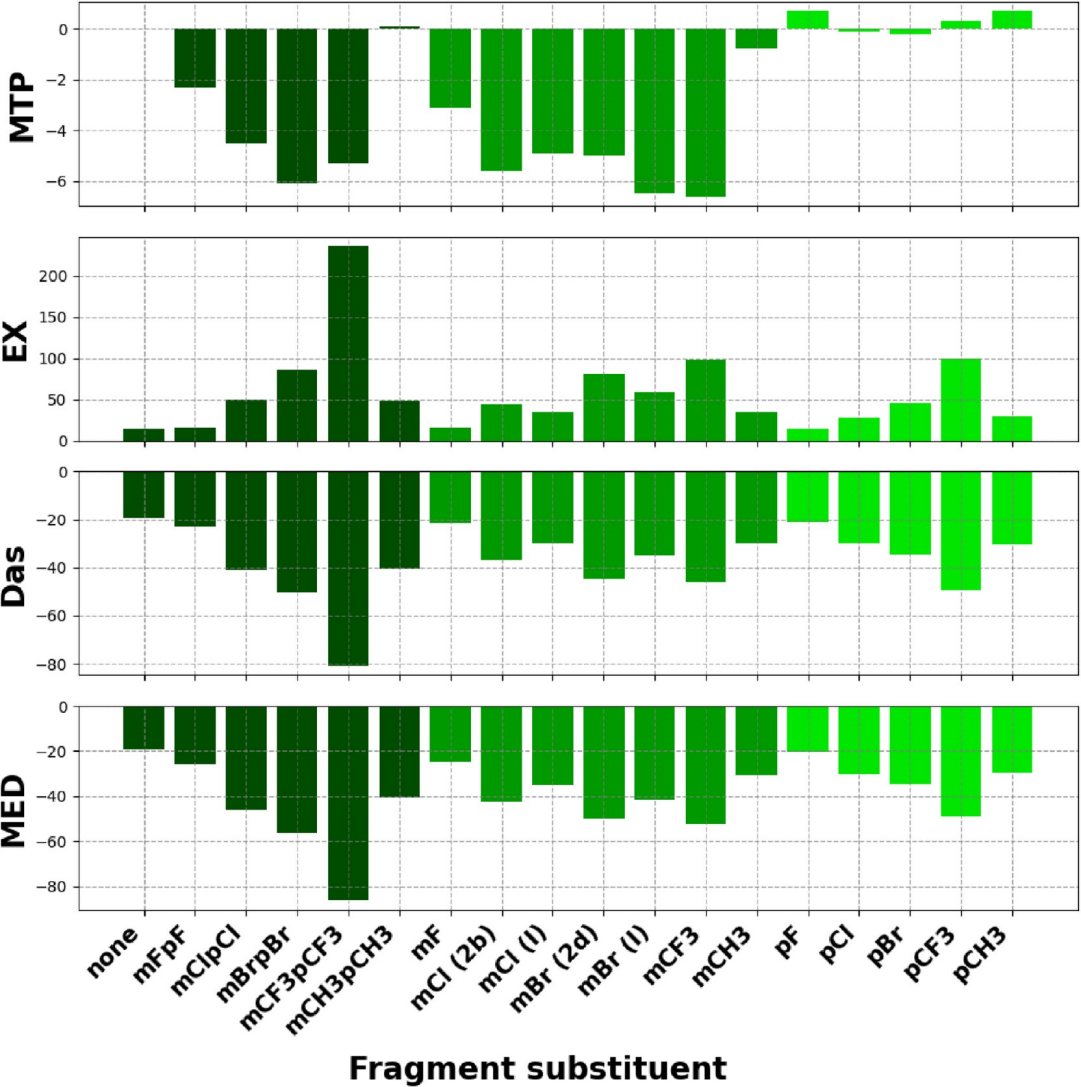
Total interaction energy components (kcal/mol) for 3 or 4-substituted phenyl tail fragments with *Tb*PTR1. The fragment definition and naming are given in Figure S7 in the SI and in the *Experimental procedures*. The energy terms are multipole electrostatic contribution *E*_EL,MTP_^(10)^ (MTP), exchange energy *E*_EX_^(10)^ (EX), approximate dispersion energy *D*_as_ (Das), and the MED energy (MED).^38,42^ The corresponding data are given in Table S5 (SI). The data for fragments with both *para* and *meta* substituents are colored dark green, with only *meta* substituents—medium green, and with only *para* substituents—light green. Abbreviations: “m”—*meta*, “p”—para. For mCl and mBr substituents, the calculation results for the two system variants are given, with the moiety positioned in the *Tb*PTR1 pocket as in compound **I** (for both mCl and mBr) and **2b** or **2d**, depending on the substituent (PDB codes: 3GN2, 9HUT, and 9HUW, respectively).

The MED energy correlates with the available activity data from Spinks et al. for **I** derivatives with a 3 or 4-substituted phenyl^24^ (Figure S8 and Table S6 in SI, *R* ^2^ = 0.76), which suggests that these modeled moieties may adopt a similar binding mode as in the previous compound series,^24^ and also validates the model. Due to the rather hydrophobic character of the analyzed interactions, the *D*_as_ energy dominates the MED energy value (ranging from ∼−80 to ∼−20 kcal/mol, Figure 9), whereas *E*_EL,MTP_^(10)^ energy values are relatively insignificant (from −7 to 1 kcal/mol). We may also note that increasing the size of the substituent at either the *meta* or the *para* position is favorable for the inhibitor–receptor interaction energy. However, increasing the substituent size too much may also result in steric clashes, which are not entirely accounted for by the MED model due to the omission of the short-range interaction energy terms (in particular, the exchange repulsion energy *E*_EX_^(10)^). This omission is often beneficial as it contributes to the MED's insensitivity to structural imperfections common in biomolecular complexes.^41^ Notably, the *E*_EX_^(10)^ value is clearly higher for the mCF_3_pCF_3_ variant. This suggests the incompatibility of the mCF_3_pCF_3_ variant with subpocket C of *Tb*PTR1 due to steric clashes. Overall, in particular for mCF_3_pCF_3_, the highest *E*_EX_^(10)^ per-residue contributions are for Trp221 and His267 (Table S7, SI), which directly neighbor the *meta*- and *para*-substituents, respectively.

Furthermore, the MED energies for *meta* substituents are overall more favorable in *Tb*PTR1 subpocket C than in the *para* substituents. The *meta*-Br and *meta*-Cl-substituted phenyls are only slightly less energetically favorable than those of the 3,4-dichlorophenyl moiety (Figure 9). Notably, these data agree with the preferential formation of halogen bonds by *meta*-Cl in subpocket C of *Tb*PTR1 in docking simulations (Figure 7a,e). Therefore, the removal of the *para*-halogen could be considered to reduce the hydrophobicity of the compounds and could be potentially favorable for targeting the more solvent-exposed pocket of *Lm*PTR1, in particular, subpocket C’.

### The Series-2 Compounds Confirm the QM Predictions, Showing Comparable Anti-PTR1 Activities to Series 1

To verify the QM predictions and analyze the binding modes of the phenyl rings with different substituents at the *meta* and *para* positions, we synthesized a second series of derivatives of **1a** (Table 2, series 2). The substituted compounds are active against *Tb*PTR1 at the low micromolar level with IC_50_s in the range 2.4–7.0 μM, similarly to the parent compound **1a** (7.6 μM). The activities against *Lm*PTR1 are distinctly lower than against TbPTR1—up to 57% inhibition at the tested concentration for **2d** with *meta*-Br (Table 2). All compounds of series 2 also show rather low activities against *T. brucei* (Table 2)—mostly in the range 27–39%, with the exception of the moderately active **2b** (51%). The tested compounds from this series are also inactive against *L. infantum*, similarly to the parent compound **1a**.

Compound **2a**, without any substituents on the phenyl ring, is slightly less active than the halogenated derivatives (IC_50_ = 14.5 μM, Table 2), indicating that the tested halogen substituents tend to increase inhibitory activity against *Tb*PTR1. Notably, despite tiny activity differences, the activities of the compounds halogenated at the *meta* position are more favorable for each of the three tested halogens (Cl, Br, and CF_3_) than their *para* equivalents, which is consistent with the QM predictions (Figure 9). The same observation can also be made for Cl and Br substitutions and *Lm*PTR1. In summary, the preference for the *meta* halogen substituent is remarkably consistent between the targets and even for different halogens.

As regards toxicity, the compounds in series 2 show relatively low toxicities against THP-1 cells (Table 2). For three compounds—**2a**, **2f**, and **2g**—CC_50_ > 100 μM, and for the three compounds—**2b**, **2c**, and **2d**, including the most active one on *T. brucei* (**2b**) – 100 μM > CC_50_ > 50 μM. The most toxic compound is **2e** (50 μM > CC_50_ > 25 μM), which also shows low activity against *T. brucei* (37%, Table 2). Early ADMET profiles show that among the current compounds, only **2e** is mitotoxic, and all compounds block hERG channels (Table S4 in SI). Furthermore, compounds **2b**–**2e** show similar problems as observed for series 1: inhibition of CYPs 2C19, 2D6, and 2C9. The exception is **2a**, the only identified liability of which is significant blocking of hERG channels.

### Differing Conformational and Subpocket Preferences of the Inhibitors in the Crystal Structures of the *Tb*PTR1 Complexes

We determined the crystal structures of five *Tb*PTR1–NADP­(H)–inhibitor complexes with compounds **1a**, **2b**, **2c**, **2d**, and **2e** to resolutions ranging from 1.58 to 1.85 Å (Figure 10; Tables S8 and S9, SI). In all complexes, the crystal asymmetric unit includes a whole *Tb*PTR1 tetramer, whose structure is highly similar to the previously reported models.^14,15,21,43,44^ The 2-aminobenzothiazole moiety of all compounds adopted exactly the same binding mode in the current crystal structures (Figure 10), as was observed in the previously published structures^22^ and in most docking results in this work (Figure 7). More details of the general characteristics of the structures are presented in the SI.

**Figure 10:**
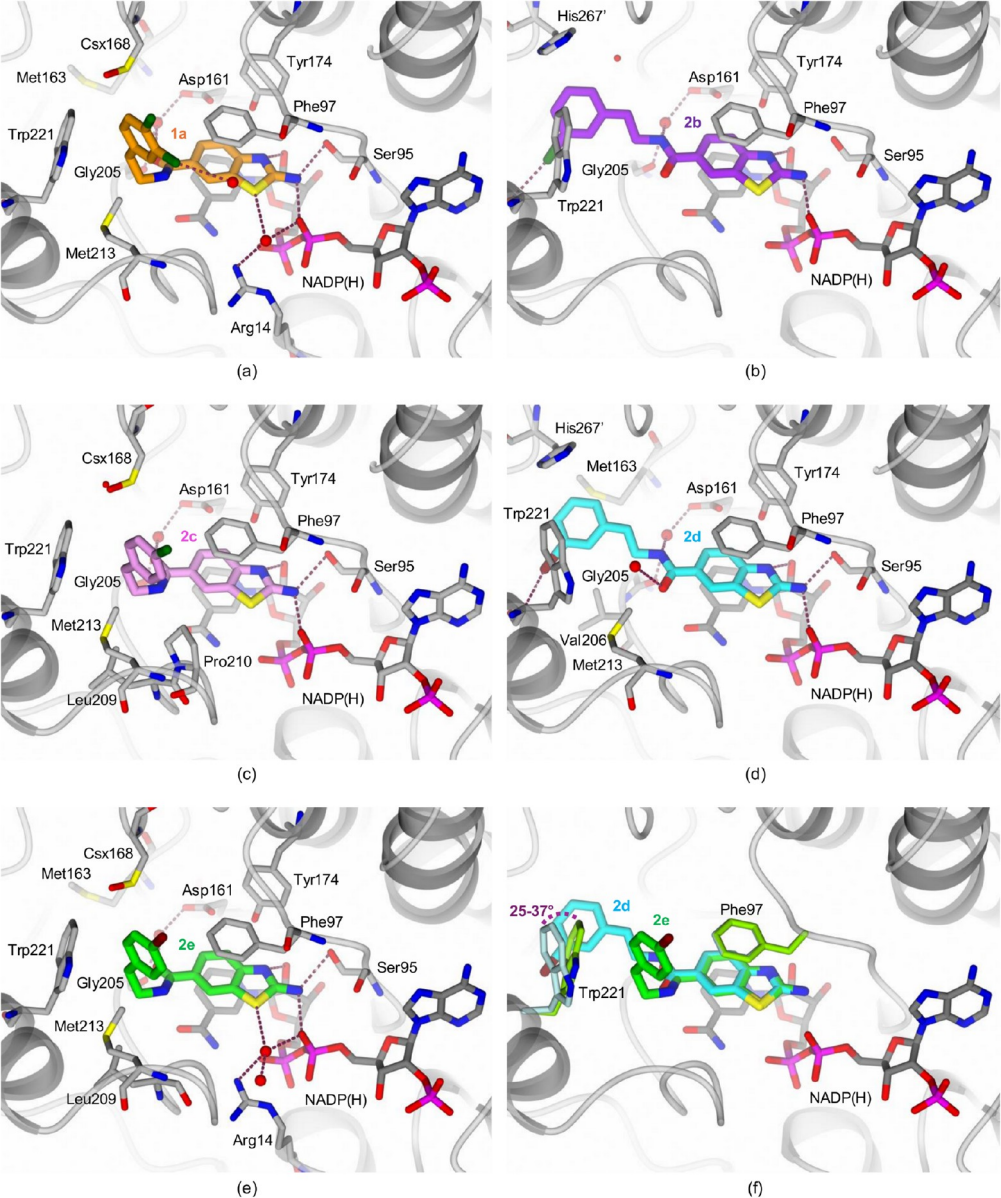
Crystal structures of the ternary complexes of *Tb*PTR1 with the cofactor NADP­(H) and the inhibitors **1a**, **2b**, **2c**, **2d**, and **2e**. The active site of *Tb*PTR1 in gray cartoon is shown with the cofactor NADP­(H), selected side chains and the inhibitors in stick representation with (a) **1a** (orange carbons), (b) **2b** (purple carbons), (c) **2c** (pink carbons), (d) **2d** (cyan carbons), and (e) **2e** (green carbons). In the complexes with **2b** and **2d**, the linker is in the *trans* configuration, and the phenyl ring is located inside subpocket C, lined by Met163, Val206, Trp221, Lys244, and His267’ (from the partner subunit). The other three compounds share a *para*-halogen group (either a chlorine in **1a** and **2c** or a bromine in **2e**) and show a *cis*-amide linker, and the phenyl moiety occupies subpocket D at the entrance of the catalytic cavity, lined by Phe97, Cys168 (oxidized to S-oxycysteine, Csx168), Leu209, Pro210, and Trp221. (f) Structural comparison between the complexes with **2d** (cyan carbons) and **2e** (green carbons). The comparison highlights the slight rotation (25–37°) of the Trp221 indole ring in the complex with **2d**, opening subpocket C to accommodate the phenyl ring of the inhibitor. Water molecules are shown as red spheres (arbitrary radius), whereas hydrogen bonds and halogen bonds are shown as red dashed lines.

### Positioning of Compound Tails Is Diverse and Flexible

The crystal structures, similarly to the docking results (Figure 7), also show that, in contrast to the 2-aminobenzothiazole moieties, the compound tails are conformationally variable, as indicated by the less well-defined shapes of their electron density maps (Figure S9, SI), and lower occupancies than for the 2-aminobenzothiazole cores (50–70% vs 70–90%, respectively, Table S10, SI). In some subunits of some structures, parts of the ligand tails are missing, whereas the 2-aminobenzothiazole cores are present (in *Tb*PTR1 complexes with **1a** and **2e**, Table S10, SI).

Furthermore, the amide linkers of the compounds adopt either a *cis* or *trans* configuration, orienting the compound tails in different subpockets of the active site (for the compounds without an additional substituent on amide N, “*cis*” means placement of 2-aminobenzothiazole and the halogenated phenyl tail on the opposite sides of the amide). Despite different orientations of the amide moieties, a conserved water-mediated interaction is always formed with the Asp161 carboxylate and the Gly205 backbone carbonyl (Figure 10a–e).

In the complexes with **2b** and **2d**, the linker is in the *trans* configuration, with its amide oxygen pointing toward the substrate loop, and the tail phenyl ring is located inside subpocket C (Figure 10b,d). In this orientation, the *meta*-halogen on the phenyl moiety (either a chlorine in **2b** or a bromine in **2d**) forms a halogen bond with the backbone carbonyl of Trp221, similarly to **I** (Figure 1a). However, the other three compounds crystallized with *Tb*PTR1, sharing a *para*-halogen (either a chlorine in **1a** and **2c** or a bromine in **2e**), have the amide linker in the *cis*-configuration with the phenyl moiety occupying subpocket D at the entrance of the catalytic cavity (Figure 10a,c,e). Notably, one of the top two predicted binding modes of **1a** is consistent with the crystallographically resolved binding mode in *Tb*PTR1 (compare Figures 6d and 10a), with the exception of the orientation of the amide bond (which, however, does not directly interact with the receptor and probably depends on the water network that cannot be fully reproduced in classical docking simulations). Finally, comparison among the compounds suggests that the presence of the *para*-halogen moiety (as in **2c**, **2e**, and **1a**) prevents binding inside subpocket C, consistent with the QM predictions (Table S5, SI), suggesting that *para-*halogens are less favorable than *meta* in subpocket C.

### The Interplay between Compound Tail Positioning and Conformation of *Tb*PTR1 Substrate Loop

As observed previously, the substrate loop that flanks subpocket C and interacts with the inhibitors’ tails adopts slightly differing conformations in different *Tb*PTR1 crystal structures.^25,45^ Notably, since the inhibitors’ tails are flexible, the preferences for either subpocket C or D may be related to the conformation of the substrate loop. Indeed, in the complexes with compounds **2b** and **2d**, targeting subpocket C, we observe a slight rotation of the indole moiety of Trp221 by 25–37° with respect to the conformation in the complexes with compound tails occupying subpocket D (Figure 10f). Therefore, the conformational change in Trp221 appears to be associated with the presence of substituents in subpocket C of *Tb*PTR1. Overall, the flexibility of Trp221 might play a role in the inhibitor binding process and may affect binding affinities of the compounds through the entropic contribution to binding free energy.

Furthermore, the compounds with tails residing in subpocket C (Figure 10), bind similarly to the reference compound **I** ^23^ (Figure 1a), and form a halogen bond with the backbone carbonyl oxygen of Trp221, but the orientation of the phenyl ring is not the same as in **I** (as shown for **2b** in Figure 11; the conformation and position of **2d** is virtually the same as for **2b**, not shown). Moreover, the *E*_MED_ interaction energies (Figure 9 and Table S5 in SI) suggest that positioning of the substituted phenyl moieties as in the crystal structures of **2b** and **2d** with *Tb*PTR1 is more energetically favorable than their **I**-derived configuration. Thus, the conformationally adaptable tails of **2b** and **2d** seem to be more optimally positioned in terms of halogenated phenyl interactions with subpocket C of *Tb*PTR1 than the corresponding moiety of **I**. This observation could be exploited in a further fragment-based compound design.

**Figure 11:**
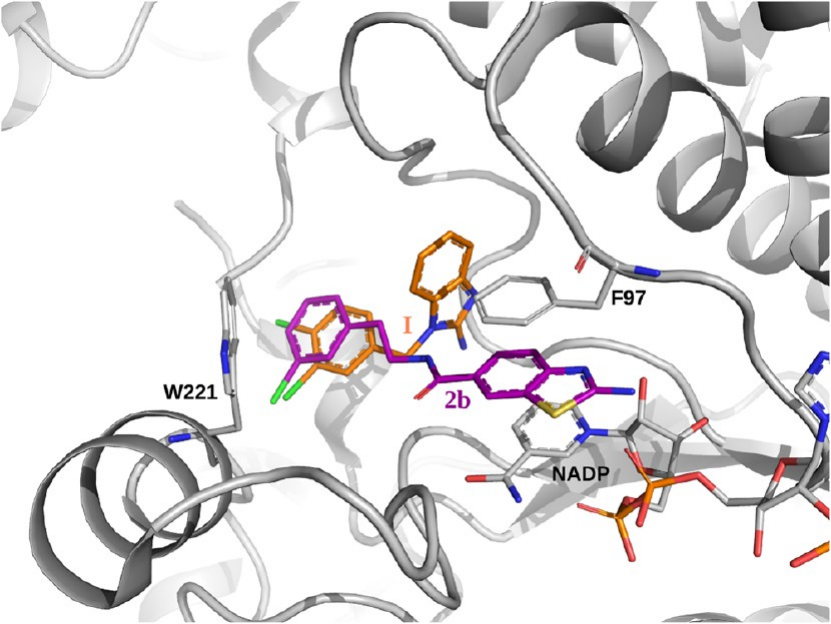
Comparison of series 2 inhibitor and parent compound binding modes with respect to *Tb*PTR1 in crystal structures. Overlay of the parent compound **I** (in orange, PDB code 3GN2) on the synthesized compound **2b** (mCl, magenta), whose crystal structure was determined here (PDB code 9HUT).

### SAR for Different Scaffolds Informed by Docking and Crystallography

In the most favorable docking poses for **1b**, we observe a similar binding pose as for **1a** in the crystal structure—with 3,4-dichlorophenyl in subpocket D (Figure S10a, SI). However, despite a favorable docking score (Table S2, SI) and overall favorable van der Waals contacts (e.g., proximity to the Phe97 side chain), the N-cyclopropyl clashes slightly with the conserved water, and it makes too close contact (heavy atoms within 3-Å distance) with the Cys168 side chain (see Figure S10a, SI). This could explain why adding N-cyclopropyl to the **1a** scaffold in **1b** does not improve binding affinity to *Tb*PTR1 (IC_50_ 7.6 and 17.1 μM, respectively, see Table 2). A similar hypothesis can be made for **V** and its N-cyclopropyl-substituted variant **1c**, a distinctly weaker binder of *Tb*PTR1 than **V** (5.4 and 71.6 μM, respectively, see Tables 1 and 2). In this case, the hydrophobic N-cyclopropyl makes contact with hydrophobic Val206 (potentially favorable nonpolar contact), but is also close to the nearby conserved water (potentially unfavorable), and interacts with the backbones of Gly205 and Ser207 (potentially unfavorable polar-nonpolar contact, Figure S10b, SI). The contact with Gly205 may be too close (about 2.8 Å) to be resolved through the system conformational variability since it involves interactions with the protein backbone.

Furthermore, the positioning of the 3,4-dichlorophenyl tail of **1a** in subpocket D of *Tb*PTR1 is likely due to the fact that the *para*-Cl of the phenyl tail does not sterically fit in subpocket C with the **1a** linker (while *meta*-chlorinated or brominated compounds, **2b** and **2d**, respectively, fit, Figure 10). However, the steric clash of *para*-chlorine may be mitigated by connecting the halogenated phenyl moiety with the benzothiazole core by the more adaptable amine-based linker. For example, **1d** is a stronger *Tb*PTR1 binder than **1c** (3.3 and 71.6 μM, respectively, Table 2). Finally, the weak activity of **1e** (18%, Table 2) is consistent with the lack of any docking pose that satisfies the constraints for 2-aminobenzothiazole and 3,4-dichlorophenyl observed in the previous and current crystal structures of *Tb*PTR1 with other compounds bearing these moieties (Figures 8 and 10).

Considering *Lm*PTR1, a higher activity of **1b** than of the other compounds (72% vs up to 38%, respectively, Table 2) may be attributed to the likely favorable combination of the following interactions in the top docking pose: (i) stacking interactions of 3,4-dichlorophenyl with His241 (observed for both **1a** and **1b** in docking studies, Figure 8a–c), (ii) favorable interactions of N-cyclopropyl with the hydrophobic residues Phe113, Leu229, and Val230, (iii) and potential formation of a halogen bond between the *meta*-Cl of 3,4-dichlorophenyl and the backbone carbonyl oxygen of His241. Among the considered scaffolds, these three types of interactions with *Lm*PTR1 are observed together in docking simulations only for **1b**. The hypothesis that halogen bonds may be also of importance in *Lm*PTR1 is further strengthened by a slight, but consistent increase of anti-*Lm*PTR1 activities for series-2 compounds with *meta*-Cl (**2b**, 48%, Table 2) and *meta*-Br (**2d**, 57%) in the tail phenyl vs their *para* equivalents (*para*-Cl [**2c**]: 32%, *para*-Br [**2e**]: 33%) and the unsubstituted variant (**2a**: 35%).

## Summary and Conclusions

We used a structure-based fragment hybridization pipeline to develop new 2-aminobenzothiazole derivatives aimed at targeting *Tb*PTR1 and *Lm*PTR1. The work builds upon previously discovered inhibitors,^22–24^ and focuses on extending the 2-aminobenzothiazole series by connecting a 3,4-dichlorophenyl moiety with various linkers. A virtual compound library was constructed and evaluated by docking simulations and ADMET property predictions, leading to the synthesis of 12 compounds. Among these, **1a** and **1b** emerged as promising inhibitors of *Tb*PTR1, with **1b** also showing a micromolar inhibition level against *Lm*PTR1 and moderate activity against *L. infantum* (close to 50% at 10 μM), demonstrating its potential as a two-species PTR1 inhibitor, which is rather unusual for 2-aminobenzothiazole derivatives.^22^ The latter activity is, however, likely related to off-target effects, which could be explored in future studies, since PTR1 is nonessential in *Leishmania*.^7,46^

In addition, the designed compounds were found to be not only less toxic for THP-1 cells than the reference compound **I**, but also active against *Tb*PTR1, and more active than **I** against *T. brucei* (Table 2). Due to the incorporation of the 2-aminobenzothiazole core, their toxicity profile is comparable to the previously synthesized 2-aminobenzothiazole derivatives (Table S4 in SI and ref 22). The compound most active against *T. brucei*, **1a**, achieved a relatively low THP-1 toxicity and a selectivity of over 16 against THP-1 cells, which could make it a candidate for an early lead.

Overall, the designed compounds present a useful stepping stone to further optimization as single- or two-species PTR1 inhibitors, effective against trypanosomatid parasites. More specifically, for the amide-based inhibitors, such as **1b**, one might consider applying a conformational locking strategy^47^ to enforce the amide conformation to be more favorable for targeting *Tb*PTR1, and to potentially increase specificity toward PTR1. Furthermore, it might be interesting to explore other potential targets for **1a** and **1b**, since we expect that additional targets play a role in their on-parasite activity.

Moreover, our exploration of halogen substituents showed that the singly halogenated compounds are slightly more active on *Tb*PTR1 than the doubly halogenated compounds, although the magnitude of the effect is small in the context of drug design. Consistent with the QM data, the substituents at the *meta* position of the phenyl ring in the analyzed scaffolds were found to be slightly energetically more favorable for both anti-*Tb*PTR1 and anti-*Lm*PTR1 activity. Although the effect on the PTR1 activity is small, it is consistent for different halogen substituents at the *para*/*meta* positions, and for both *Tb*PTR1 and *Lm*PTR1 (Table 2), showing surprising sensitivity of the QM method. Unfortunately, removing *para*-chlorine from the doubly chlorinated **1a** phenyl also significantly reduced the low micromolar antiparasite activity. The predictions of the QM calculations were confirmed by the crystallographic data and showed that the position of the halogen substituents affected the positioning of the flexible halogenated phenyl tails in subpocket C vs D.

Overall, the data provide insights into the halogen interactions with PTR1, although the halogen substitutions do not play a dominant role in anti-PTR1 activities. The approach used here may be useful for other ligand series, in particular, where halogens have a greater effect on compound activity.

The crystallographic data clearly demonstrate the flexibility of the halogenated phenyl tails, which adopt different conformations depending on the linker type and subpocket interactions. Notably, there is an interplay between the Trp221 side chain rotational state and ligand binding in either subpocket C or D. Importantly, we also found that the halogenated phenyl rings of compounds **2b** and **2d** are positioned differently than the ring of compound **I** in crystallographic structures and have QM interaction energies in subpocket C that are more favorable than that of compound **I**. These structural insights will support future compound optimization. In future work, one might consider studying in more detail how the dynamics of the PTR1-inhibitor complexes affect the formation of halogen bonds and compound activities, as suggested by the crystallographic data. Also, exchanging 2-aminobenzothiazole for another aromatic ring more compatible with PTR1 could be considered.

In summary, we have described a combined computational and experimental approach to designing, synthesizing, and evaluating the SAR for new halogenated 2-aminobenzothiazole derivatives as PTR1 and parasite inhibitors. The work offers insights into the molecular basis of inhibitor binding and activity, providing a guide for further optimization of these compounds for improved therapeutic efficacy and selectivity.

## Experimental Procedures

### Computational Methods

#### Structure Preparation, Analysis, and Molecular Docking Simulations

The Maestro suite 2015-2 ^48^ was used in all the calculation steps described below. The OPLS_2005 force field^49^ (the default force field when the study was started) was used in the preparation process. The molecular structures of receptors and chemical compounds were prepared similarly to those reported previously^14^ (see the details in SI). ADMET properties of the prepared compounds were evaluated with the QikProp tool of the Maestro suite, and PAINS filtering was performed (more details in SI). Docking grids of size 40 × 40 × 40 Å centered on Phe97 for *Tb*PTR1 and on Phe113 for *Lm*PTR1 were calculated, with internal boxes of size 15 × 15 × 15 Å. The sizes were increased from the default values to account for the relatively large PTR1 pocket, considering the placement of ligands in different subpockets. Molecular docking simulations were performed using the Glide software.^50,51^ Standard settings for the ligand van der Waals radii were kept. After initial tests, the SP docking protocol was used. The docking method was validated by redocking and cross-docking simulations (ligand RMSDs are provided in Table S11, SI). Ligands were treated as flexible; nitrogen inversions and ring conformations were sampled. Biased sampling was only performed for amide torsions, which were penalized if in a nonplanar conformation. Epik state penalties were added to the docking score, and the planarity of conjugated π-groups was enhanced. Twenty poses were subjected to postdocking energy minimization with a pose-rejection threshold of 0.5 kcal/mol, and up to 10 final docking solutions were retained. In addition, the docking procedure used a special treatment of halogen atoms as halogen bond donors. The poses for each ligand were sorted based on the docking score and further analyzed as described in the Results. It is worth noting that the docking scores are not accurate estimates of binding free energy, so poses other than the top-ranked one (according to the score) were considered as probable binding modes. If not otherwise noted, the three top poses for each compound were subjected to a more detailed analysis and visual inspection.

#### QM Calculations of *Tb*PTR1–Ligand Interaction Energy

To evaluate the halogen interactions in a systematic, nonempirical manner, the inhibitor was truncated to the substituted benzene (Figure S7 in SI). Together with compound **I** (mClpCl), shown in Figure S7a, 18 ligand fragments were analyzed. The *meta*- and *para*-substitutions (also referred to as “m” and “p,” respectively) with a halogen series (Cl, F, Br, CF_3_), methyls, and unsubstituted variants, were considered. The compound variant naming is shown in Figure S7a; e.g., the fragment with both *para* and *meta* positions of phenyl is named mClpCl, while the fragment with a substitution only at the *meta* position is named mCl.

Binding poses of the analyzed compounds were modeled on the basis of the crystallographic binding mode of ligand **I** in the PTR1 binding site (PDB code 3GN2 ^23^), and prepared in the same way as the receptors for docking simulations. The modified inhibitor structures were built and minimized within the *Tb*PTR1 pocket and then truncated to the final fragments. The *Tb*PTR1 ligand binding energy was calculated for the binding pocket model composed of 11 amino acid residues within 4 Å of any atom of the **I** ligand fragment (Figure S7b, SI), i.e., Met163, Gln166, Pro167, Cys168, Gly205, Val206, Trp221, Lys224, Leu263 (chain A of *Tb*PTR1), and His267 and Ala268 (chain D).

Binding energies were calculated using the MED model (MED = *E*_EL,MTP_^(10)^ + *D*_as_), including the long-range interaction energy terms: multipole electrostatic (*E*_EL,MTP_^(10)^) and approximate dispersion energy *D*_as_.^38,39^ To facilitate the analysis of possible steric clashes, the short-range exchange repulsion energy (*E*_EX_^(10)^) was calculated following the Hybrid Variation-Perturbation Theory^52,53^ (HVPT) as the difference between the first-order Heitler–London energy and the first-order electrostatic term, based on the HF/def2TZVP wave function obtained with counterpoise correction to alleviate the basis set superposition error.^54–56^ The total binding energy of a given compound was computed as the sum of the interaction energy values obtained for amino acid residue-ligand dimers. The remaining calculation details are presented in the SI.

### Chemistry

#### Synthetic Procedures

All commercially available chemicals and solvents were of reagent grade and were used without further purification unless otherwise specified. Synthesis of intermediates is reported in SI. The following solvents were used: tetrahydrofuran (THF), ethyl ether (Et_2_O), dimethyl sulfoxide (DMSO), ethyl acetate (EtOAc), dichloromethane (DCM), dimethylformamide (DMF), methanol (MeOH), and acetonitrile (ACN). Reactions were monitored by thin-layer chromatography on silica gel plates (60F-254, E. Merck) and visualized with UV light, cerium ammonium sulfate, or alkaline KMnO_4_ aqueous solution. NMR spectra were recorded on a Bruker 400 spectrometer with ^1^H at 400.134 MHz and ^13^C at 100.62 MHz. Proton chemical shifts were referenced to the solvent residual peaks. Chemical shifts are reported in parts per million (ppm, δ units). Coupling constants are reported in Hertz (Hz). Splitting patterns are designed as s, singlet; d, doublet; t, triplet; q, quartet; dd, double doublet; m, multiplet; b, broad. High-resolution mass spectra were obtained using MeOH as solvent and a Thermo Fisher HPLC-MS UltiMate 3000 mass spectrometer, with a hybrid Q Exactive quadrupole–HESI-II electron spray orbitrap mass analyzer. Purity was determined using a HPLC-UV/vis (Agilent Infinity II, see Table S12 and chromatograms in Figure S11, SI). Chromatographic separations were carried out on a RP Kinetex 2.6 μm Biphenyl 100 Å maintained at 30 °C. The separation was performed under gradient conditions using solvent A *t*_0_ 95–5% over 22 min at a flow rate of 1 mL/min with eluents A (H_2_O + 0.1% Formic acid) and B (ACN + 0.1% Formic acid). The separation was monitored using wavelengths of 220 and 254 nm. Purity detection was obtained with the HPLC method of % area calculation and blank subtraction. The representative synthesized compounds showed a purity level above 95% by HPLC-UV/vis analysis.

#### General Procedure for the Synthesis of 6-carboxyamide-2-aminobenzothiazoles **1a**, **1b**, **1c**, **1e**, **2a**, **2b**, **2c**, **2d**, **2e**, **2f**, and **2g**

A solution of 2-amino-6-benzothiazolcarboxylic acid (1 equiv) was prepared in anhydrous DMF under a nitrogen atmosphere at 0 °C. To this solution were added EDC·HCl (1 equiv) and HOBt (1 equiv). The mixture was stirred at 0 °C for 10 min. Subsequently, a solution of the appropriate amine (1 equiv) in anhydrous DMF was added dropwise, followed by the addition of TEA (1 equiv). The temperature was then allowed to rise spontaneously, and the mixture was stirred overnight at room temperature. After completion, DMF was removed under reduced pressure, and the residue was suspended in water and extracted with ethyl acetate. The organic layer was washed three times with a saturated solution of Na_2_CO_3_ and then with brine, dried over anhydrous Na_2_SO_4_, and concentrated. The crude product was crystallized from DCM or diethyl ether to yield the desired product.

##### 2-amino-*N*-(3,4-dichlorophenethyl)­benzo­[*d*]­thiazole-6-carboxamide (**1a**)

White solid, 63% yield. ^1^H NMR (400 MHz, DMSO-*d*_6_) δ 2.74–2.87 (m, 2H), 3.48 (t, *J* = 5.2 Hz, 2H), 6.66 (s, 2H), 7.05 (dd, *J* = 1.5, 7.2 Hz, 1H), 7.31–7.37 (m, 2H), 7.56 (dd, *J* = 1.5, 7.5 Hz, 1H), 7.69 (d, *J* = 7.4 Hz, 1H), 8.30 (d, *J* = 1.5 Hz, 1H), 8.57 (s, 1H). ^13^C NMR (100 MHz, DMSO-*d*_6_) δ 33.97, 41.77, 118.63, 121.08, 123.23, 127.99, 129.54, 130.28, 131.23, 131.30, 131.62, 133.37, 135.62, 155.96, 167.42, 169.16. HRMS *m*/*z* [M + H]^+^ Calcd for C_16_H_13_Cl_2_N_3_OS: 365,0156. Found: 365.0155.

##### 2-amino-*N*-cyclopropyl-*N*-(3,4-dichlorophenethyl)­benzo­[*d*]­thiazole-6-carboxamide (**1b**)

White solid, 45% yield. ^1^H NMR (400 MHz, DMSO-*d*_6_) δ 0.72–1.14 (m, 3H), 2.25 (p, *J* = 9.2 Hz, 1H), 2.81–3.14 (m, 2H), 3.53 (t, *J* = 7.1 Hz, 2H), 6.66 (s, 2H), 7.14 (ddd, *J* = 1.2, 2.3, 7.5 Hz, 1H), 7.26–7.57 (m, 3H), 7.65 (d, *J* = 7.4 Hz, 1H), 7.85 (d, *J* = 1.4 Hz, 1H). ^13^C NMR (101 MHz, DMSO) δ 9.05, 31.35, 35.04, 45.35, 118.85, 121.08, 122.61, 128.14, 128.77, 130.22, 131.34, 131.43, 132.54, 133.24, 135.53, 156.15, 169.16, 172.70. HRMS *m*/*z* [M + H]^+^ Calcd for C_19_H_17_Cl_2_N_3_OS: 405,0469. Found: 405.0470.

##### 2-amino-*N*-cyclopropyl-*N*-(3,4-dichlorobenzyl)­benzo­[*d*]­thiazole-6-carboxamide (**1c**)

White solid. 58% yield.^1^H NMR (400 MHz, DMSO-*d*_6_) δ 0.19 (t, *J* = 3.2 Hz, 2H), 0.25–0.39 (m, 2H), 3.12 (s, 2H), 3.88–4.01 (m, 1H), 7.01–7.17 (m, 2H), 7.17–7.28 (m, 1H), 7.32–7.42 (m, 2H), 7.51 (s, 1H), 7.71 (dd, *J* = 1.6, 10.5 Hz, 1H). ^13^C NMR (100 MHz, DMSO) δ 9.05, 32.72, 51.04, 118.85, 121.08, 122.61, 127.76, 128.77, 129.60, 130.09, 130.27, 131.33, 132.54, 137.74, 156.15, 169.16, 172.26. HRMS *m*/*z* [M + H]^+^ Calcd for C_18_H_15_Cl_2_N_3_OS: 391.0313. Found: 391.0315.

##### 6-((cyclopropyl­(3,4-dichlorobenzyl)­amino)­methyl)­benzo­[_*d*_]­thiazol-2-amine (**1d**)

White solid, 36% yield. ^1^H NMR (400 MHz, DMSO-*d*_6_) δ 1.12 (tdd, *J* = 8.2, 5.3, 1.0 Hz, 2H), 1.19–1.41 (m, 2H), 2.22 (p, J = 5.4 Hz, 1H), 3.69 (dt, J = 2.2, 0.9 Hz, 4H), 7.19 (dddt, J = 11.3, 8.2, 2.1, 0.8 Hz, 2H), 7.27–7.53 (m, 2H), 7.53–7.96 (m, 2H). ^13^C NMR (100 MHz, DMSO) δ 7.50, 36.94, 58.93, 59.14, 116.86, 121.83, 126.76, 127.70, 129.64, 129.78, 130.02, 130.04, 131.78, 135.43, 138.30, 151.31, 165.50. HRMS *m*/*z* [M + H]^+^ Calcd for C_18_H_18_Cl_2_N_3_S: 378.0593. Found: 378.0590.

##### 2-amino-*N*-(3,4-dichlorophenethyl)-*N*-phenylbenzo­[*d*]­thiazole-6-carboxamide (**1e**)

White solid, 15% yield. ^1^H NMR (400 MHz, MeOD) δ 2.92 (d, *J* = 7.7 Hz, 2H), 3.56 (t, *J* = 7.6 Hz, 2H), 7.15–7.21 (m, 1H), 7.21–7.33 (m, 2H), 7.35 (d, *J* = 3.8 Hz, 4H), 7.63–7.75 (m, 2H), 8.20 (d, *J* = 1.5 Hz, 1H). HRMS *m*/*z* [M + H]^+^ Calcd for C_22_H_17_Cl_2_N_3_OS: 441.0469. Found: 441.0470.

##### 2-amino-*N*-phenethylbenzo­[*d*]­thiazole-6-carboxamide (**2a**)

White solid, 72% yield. ^1^H NMR (400 MHz, DMSO-*d*_6_) δ 2.85 (dd, *J* = 6.6, 8.4 Hz, 2H), 3.40–3.65 (m, 2H), 6.93–7.53 (m, 6H), 7.64–7.81 (m, 3H), 8.14 (d, *J* = 1.8 Hz, 1H), 8.43 (t, *J* = 5.6 Hz, 1H). ^13^C NMR (101 MHz, DMSO) δ 35.18, 40.88, 116.78, 120.14, 124.91, 126.00, 127.11, 128.28, 128.59, 130.75, 139.58, 155.10, 165.83, and 168.33. HRMS *m*/*z* [M + H]^+^ Calcd for C_16_H_15_N_3_OS: 297.0936. Found: 297.0935.

##### 2-amino-*N*-(3-chlorophenethyl)­benzo­[*d*]­thiazole-6-carboxamide (**2b**)

White solid, 48% yield. ^1^H NMR (400 MHz, DMSO-*d*_6_) δ 2.86 (t, *J* = 7.2 Hz, 2H), 3.50 (td, *J* = 5.6, 7.1 Hz, 2H), 6.93–7.41 (m, 5H), 7.58–7.82 (m, 3H), 8.12 (dd, *J* = 0.5, 1.8 Hz, 1H), 8.42 (t, *J* = 5.6 Hz, 1H). ^13^C NMR (101 MHz, DMSO) δ 34.61, 40.45, 116.77, 120.13, 124.90, 126.01, 127.04, 127.43, 128.52, 130.06, 130.75, 132.84, 142.22, 155.11, 165.91, 168.34. HRMS *m*/*z* [M + H]^+^ Calcd for C_16_H_14_ClN_3_OS: 331.0546. Found: 331.0546.

##### 2-amino-*N*-(4-chlorophenethyl)­benzo­[*d*]­thiazole-6-carboxamide (**2c**)

White solid, 51% yield. ^1^H NMR (400 MHz, DMSO-*d*_6_) δ 2.84 (t, *J* = 7.2 Hz, 2H), 3.41–3.64 (m, 2H), 7.18–7.30 (m, 2H), 7.30–7.40 (m, 3H), 7.67–7.79 (m, 3H), 8.12 (d, *J* = 1.8 Hz, 1H), 8.41 (t, *J* = 5.6 Hz, 1H). ^13^C NMR (101 MHz, DMSO) δ: 34.38, 40.60, 116.78, 120.13, 124.91, 127.04, 128.16, 130.51, 130.65, 130.76, 138.64, 155.12, 165.88, 168.35. HRMS *m*/*z* [M + H]^+^ Calcd for C_16_H_14_ClN_3_OS: 331.0546. Found: 331.0545.

##### 2-amino-*N*-(3-bromophenethyl)­benzo­[*d*]­thiazole-6-carboxamide (**2d**)

Pale yellow solid, 43% yield. ^1^H NMR (400 MHz, DMSO-*d*_6_) δ 2.85 (t, *J* = 7.2 Hz, 2H), 3.42–3.59 (m, 2H), 7.26 (dd, *J* = 0.9, 5.2 Hz, 2H), 7.33 (s, 1H), 7.35–7.44 (m, 1H), 7.47 (q, *J* = 1.2 Hz, 1H), 7.61–7.79 (m, 3H), 8.11 (d, *J* = 1.8 Hz, 1H), 8.41 (t, *J* = 5.6 Hz, 1H). ^13^C NMR (101 MHz, DMSO) δ 34.57, 41.11, 117.97, 120.42, 122.10, 122.57, 125.66, 128.84, 128.88, 129.50, 129.63, 130.96, 137.72, 155.31, 166.76, 168.50. HRMS *m*/*z* [M + H]^+^ Calcd for C_16_H_14_BrN_3_OS: 375.0041. Found: 375.0035.

##### 2-amino-*N*-(4-bromophenethyl)­benzo­[*d*]­thiazole-6-carboxamide (**2e**)

Pale yellow solid, 37% yield. ^1^H NMR (400 MHz, DMSO-*d*_6_) δ 2.83 (t, *J* = 7.2 Hz, 2H), 3.40–3.54 (m, 2H), 7.09–7.30 (m, 2H), 7.34 (d, *J* = 8.4 Hz, 1H), 7.41–7.57 (m, 2H), 7.62–7.83 (m, 3H), 8.12 (d, *J* = 1.8 Hz, 1H), 8.40 (t, *J* = 5.6 Hz, 1H). ^13^C NMR (101 MHz, DMSO) δ: 34.43, 40.53, 116.77, 119.10, 120.13, 124.90, 127.02, 130.75, 130.94, 131.09, 139.07, 155.11, 165.86, 168.34. HRMS *m*/*z* [M + H]^+^ Calcd for C_16_H_14_BrN_3_OS: 375.0041. Found: 375.0041.

##### 2-amino-*N*-(4-(trifluoromethyl)­phenethyl)­benzo­[*d*]­thiazole-6-carboxamide (**2f**)

White solid, 61% yield. ^1^H NMR (400 MHz, DMSO-*d*_6_) δ 2.86 (dd, *J* = 4.6, 5.7 Hz, 2H), 3.47 (t, *J* = 5.2 Hz, 2H), 6.66 (s, 2H), 7.14 (s, 1H), 7.45–7.62 (m, 3H), 7.68 (d, *J* = 7.5 Hz, 1H), 8.33 (d, *J* = 1.4 Hz, 1H), 8.57 (s, 1H). ^13^C NMR (100 MHz, DMSO-*d*_6_) δ 34.99, 40.50, 118.63, 121.08, 123.23, 125.58, 125.62, 129.22, 129.25, 129.54, 131.62, 136.78, 155.96, 167.42, 169.16. HRMS *m*/*z* [M + H]^+^ Calcd for C_17_H_14_F_3_N_3_OS: 365.0810. Found: 365.0810.

##### 2-amino-*N*-(3-(trifluoromethyl)­phenethyl)­benzo­[*d*]­thiazole-6-carboxamide (**2g**)

White solid, 69% yield. ^1^H NMR (400 MHz, DMSO-*d*_6_) δ 2.57–3.01 (m, 2H), 3.48 (t, *J* = 5.2 Hz, 2H), 6.66 (s, 2H), 7.09–7.24 (m, 1H), 7.28 (t, *J* = 7.4 Hz, 1H), 7.37–7.52 (m, 2H), 7.56 (dd, *J* = 1.6, 7.6 Hz, 1H), 7.68 (d, *J* = 7.3 Hz, 1H), 8.33 (d, *J* = 1.5 Hz, 1H), 8.57 (s, 1H). ^13^C NMR (100 MHz, DMSO-*d*_6_) δ 35.23, 41.77, 118.63, 121.08, 122.98, 123.02, 123.23, 125.65, 125.69, 128.79, 128.81, 129.54, 131.62, 132.40, 155.96, 167.42, 169.16. HRMS *m*/*z* [M + H]^+^ Calcd for C_17_H_14_F_3_N_3_OS: 365.0810. Found: 365.0815.

### *Tb*PTR1 and *Lm*PTR1 Indirect Enzyme Assay

The in vitro assays used in the current study were based on those reported in the literature.^57^ As PTR1 enzymes use dihydrobiopterin (H_2_B) as a substrate and also require NADPH for the reaction, the reduction of H_2_B to tetrahydrobiopterin by PTR1 is nonenzymatically linked with the reduction of cytochrome c in this assay, which is detected at 550 nm. The formation of cyt c Fe^2+^ results in an increase in the photometric readout. *Tb*PTR1 and *Lm*PTR1 activity was assayed in a buffer containing 20 mM sodium citrate (pH 6.0). The final reaction mixture contained the test compound at a range of concentrations and *Tb*PTR1/*Lm*PTR1 (6.0 nM/12 nM), H_2_B (0.3 μM/3 μM), cytochrome c (100 μM/100 μM), and NADPH (500 μM/500 μM). The final assay volume was 50 μL in 384-well clear plates (Greiner Bio-One, 781101). Compound screening was performed by the addition of the compound to the assay plates (in 100% DMSO), followed by the addition of 45 μL Reaction Mix (enzyme, H_2_B cytochrome c in 20 mM sodium citrate buffer). A pre-read was made at 550 nm using an EnVision Multilabel Reader 2103 (PerkinElmer Inc., US), followed by incubation of the assay plates at 30 °C for 10 min. The reaction was initiated by the addition of 5 μL of NADPH (5 mM in ultrapure water) followed by kinetically reading the assay plates at 550 nm using the EnVision Multilabel Reader at 10, 20, 30, 40, and 50 min. The slope of each assay well was calculated. The screening data were analyzed using ActivityBase (IDBS), and for outlier elimination in the control wells, the 3-σ method was applied. Based on the slope, the data were normalized to the positive control methotrexate for *Tb*PTR1/*Lm*PTR1 (1 μM/50 μM, yielding 100% inhibition) and negative controls (1% DMSO, yielding 0% inhibition), and % inhibition was calculated for all samples. The measurement at time 0 min was used to flag optically interfering samples. Each compound was tested in triplicate, and the pIC_50_ value, standard deviation, Hill slope, and minimum and maximum signals for each dose–response curve were obtained using a 4-parameter logistic fit in the XE module of Activity Base (IDBS),^9^ as also reported previously.^14,18,21,22^

### *Tb*PTR1 Kinetic Direct Enzyme Assay

A direct kinetic assay was carried out to measure NADPH consumption over time using a Jasco V730 double-beam spectrophotometer. The assay was conducted as reported in ref 58. Thawed protein was incubated in 40 mM citrate buffer (pH 3.7) at a final concentration of 30 nM, along with the endogenous substrate at 27 μM. The reaction was carried out in a Kartell semimicro disposable polystyrene plastic cuvette (Kartell LABWARE, Milan, Italy). The inhibitor was delivered in the concentration range of 0.5–50 mM, depending on the inhibitor potency, from an initial DMSO stock solution previously prepared (5 or 10 mM) and preincubated with the enzyme for 15 min at 20 °C. The maximum DMSO concentration was 2% in the assay solution. Each compound was tested in duplicate with a control assay without the inhibitor. The experiment was conducted 3 times. The reaction was initiated by adding NADP­(H) to a final concentration of 134 μM in the cuvette, and the change in absorbance (ΔOD/min) at λ = 340 nm was monitored over 180 s at 25 °C with 27 μM. Six different concentrations were analyzed and fitted to a three-parameter logistical (3PL) model. A 95% confidence interval (CI) was used to calculate the standard error for each curve fitting, and a Student’s *t* test was performed for each duplicate with a significance threshold of *p* ≤ 0.05. Data fitting and statistical analysis were carried out using the GraphPad Prism Suite (2021).

### Cell Cultures

#### THP-1

Human leukemia cell line THP-1 (ATCC TIB202) cells were cultured in RPMI-1640 medium supplemented with 10% heat-inactivated fetal bovine serum (FBS), 2 mM l-glutamine, 100 IU/mL penicillin/streptomycin, and 20 mM HEPES. The cell line was maintained in a humidified incubator at 37 °C with 5% CO_2_. Subcultures were performed every 3 days in 20 mL of media at a concentration of 2 × 10^5^ cells/mL in a T75 flask. All cell culture reagents were purchased from Lonza Bioscience (Morrisville, NC).

#### Trypanosoma brucei

*Trypanosoma brucei* Lister 427 bloodstream forms were cultivated in a humidified incubator maintained at 37 °C with 5% CO_2_. The parasites were grown in 5 mL T25 ventilated flasks containing complete HMI-9 medium.^59^ The medium was supplemented with 10% heat-inactivated FBS and 100 IU/mL penicillin/streptomycin. To maintain the cultures, subpassages were performed every 2 days at a concentration of 1 × 10^4^ cells/mL.

#### Leishmania infantum

Luciferase-expressing *L. infantum* (MHOM/MA/67/ITMAP-263) axenic amastigotes were cultured in MAA/20 medium at 37 °C with 5% CO_2_.^60^ The parasites were maintained in 5 mL T25 ventilated flasks and subcultured every 7 days at a concentration of 1 × 10^6^ cells/mL.

### Cytotoxicity Assay

The cytotoxicity of the compounds on THP-1-derived macrophages was assessed using the colorimetric MTT assay (3-(4,5-dimethylthiazol-2-yl)-2,5-diphenyl tetrazolium bromide) as described elsewhere.^61^ THP-1 cells were suspended in RPMI complete medium at a density of 1 × 10^6^ cells/mL, and 100 μL/well was seeded in a 96-well plate. The cells were differentiated into macrophages by adding 40 ng/mL of phorbol-myristate 13-acetate (PMA, Sigma, Saint Louis, MI, USA) for 24 h, followed by replacement with fresh medium for another 24 h. Subsequently, the cells were incubated with 100 μL of compounds, ranging from 100 to 12.5 μM, diluted in RPMI complete medium. Each condition was tested in triplicate. After 72 h of incubation at 37 °C with 5% CO_2_, the medium was removed, and 200 μL of 0.5 mg/mL MTT solution diluted in RPMI was added. The plates were incubated for an additional 4 h, after which 160 μL of the media was removed and replaced with 160 μL of 2-propanol. Absorbance was read at 570 nm using a Synergy 2 Multi-Mode Reader (Biotek, Winooski, VT, USA). Cytotoxicity was evaluated by presenting the CC_50_ (the concentration of the drug that reduces cell viability by 50%) interval or by determining the CC_50_ value through nonlinear regression analysis using GraphPad Prism version 8.1.1 for Windows (GraphPad Software, San Diego, CA, USA). Results represent the average of at least three independent experiments.

### Activity against *T. brucei*

The efficacy of the compounds against *Trypanosoma brucei* *brucei* L427 wild-type (WT) bloodstream forms was assessed using a resazurin-based assay as described elsewhere.^18^ *T. b. brucei* L427 bloodstream forms were prepared at a cell density of 5 × 10^3^ cells/mL in supplemented complete medium. Serial dilutions of the test compounds were prepared in a final volume of 100 μL, and 100 μL of the parasite suspension was added to each well containing the compound dilutions, resulting in a total volume of 200 μL per well. A dose–response curve for pentamidine was included as a quality control in each assay, and each condition was tested in duplicate. The plates were incubated for 72 h under the specified conditions for the parasite. After the incubation period, 20 μL of a 0.5 mM resazurin solution was added to each well, and the plates were incubated for an additional 4 h under the same conditions. Fluorescence was measured at an excitation wavelength of 544 nm and an emission wavelength of 590 nm using a Synergy 2 Multi-Mode Reader (Biotek, Winooski, VT, USA). The results were expressed as the percentage of parasite growth inhibition compared to the control (untreated parasites) and represented the average of at least three independent experiments. The half-maximal inhibitory concentration (IC_50_) values, representing the concentration required to inhibit 50% of parasite growth, were determined by nonlinear regression analysis using GraphPad Prism version 8.1.1 for Windows (GraphPad Software, San Diego, CA, USA).

### Activity against *L. infantum* Intracellular Amastigotes

The activity against *Leishmania infantum* intracellular amastigotes was evaluated following the method described with modifications.^62^ THP-1 cells were differentiated into macrophages with PMA as described above. The cells were then infected with *L. infantum* axenic amastigotes expressing episomal luciferase at a macrophage-to-amastigote ratio of 1:10 for 4 h at 37 °C and 5% CO_2_. Noninternalized parasites were washed away, and compounds were added at various concentrations to a final volume of 100 μL. A dose–response curve for miltefosine was included in all assays as a quality control. Each condition was tested in quadruplicate. After 72 h of incubation, the medium was replaced with 100 μL of PBS, and 25 μL of Glo-lysis buffer from the Steady-Glo Luciferase Assay System (Promega, Madison, WI, USA) was added. The plates were agitated at 100 rpm for 10 min at room temperature. Subsequently, 30 μL of the Steady-Glo reagent (Promega, Madison, WI, USA) was added and incubated for 15 min in the dark under the same conditions. A total of 140 μL from each well was transferred to white-bottom 96-well plates, and luminescence intensity was measured using a Synergy 2 multimode reader (Biotek, Winooski, VT, USA). The antileishmanial effect was evaluated by comparison of the nontreated infected cells. The IC_50_ was determined (for miltefosine) through nonlinear regression analysis using GraphPad Prism version 8.1.1 for Windows (GraphPad Software, San Diego, CA, USA). Results represent the average of at least three independent experiments.

### Crystal Structure Determination for *Tb*PTR1 Ternary Complexes

#### Protein Expression and Purification

Recombinant *Tb*PTR1 was expressed as a histidine-tagged protein and purified by established methods.^18^ Briefly, *Tb*PTR1 was produced in *E. coli* BL21­(DE3) cells cultured at 37 °C in SuperBroth medium supplemented with 100 mg/L ampicillin to mid log phase. Protein overexpression was then induced by adding 1 mM isopropyl-β-d-thiogalactopyranoside and culturing cells for 16 h at 24 °C under vigorous aeration. Cells, collected by centrifuge (3500*g*, 20 min, 8 °C), were resuspended in buffer A (50 mM Tris-HCl, pH 7.5, and 250 mM NaCl) supplemented with 20 mM imidazole and 0.5 mg/mL lysozyme, and then disrupted by sonication after 30 min incubation on ice. The target protein was purified by immobilized metal-affinity chromatography (IMAC) through a HisTrap FF 5 mL column (Cytiva), followed by size-exclusion chromatography (SEC) on a HiLoad 16/600 Superdex 200 pg column (Cytiva). The resulting protein sample was dialyzed overnight in 20 mM Tris-HCl, pH 7.5 (membrane cutoff 10 kDa) and concentrated to 10–14 mg/mL; the high purity (>98%) was confirmed by SDS-PAGE analysis and MALDI-TOF mass spectrometry. The final protein yield was established as approximately 80 mg/L bacterial culture. The purified sample of His-tagged *Tb*PTR1, supplemented with 10 mM DTT, was stored at 8 °C until required.

#### Protein Crystallization

*Tb*PTR1 crystals were obtained by the vapor-diffusion hanging-drop technique,^63^ according to established procedures.^18,21,44^ Briefly, drops were prepared by mixing equal volumes of protein (10–14 mg/mL, in 20 mM TRIS, pH 7.5, and 10 mM DTT) and precipitant (1.8–2.2 M sodium acetate and 0.1 M sodium citrate, pH 5) solutions. Well-ordered monoclinic crystals grew within a few days of equilibration at room temperature against a 600 μL reservoir. *Tb*PTR1-NADP­(H)-inhibitor complexes were obtained by the soaking technique on preformed protein crystals.^63^ Compounds, solubilized in DMSO (40 mM stock solution), were separately added to the crystallization drops (final inhibitor concentration of 2–4 mM), and the mixtures were incubated at room temperature for 1–4 h. Crystals were then washed in the cryoprotectant solution (made of precipitant supplemented with 30% v/v glycerol) and flash frozen in liquid nitrogen.

#### X-Ray Data Collection, Structure Solution, and Refinement

X-ray diffraction images were collected using synchrotron radiation at Diamond Light Source (DLS, Didcot, United Kingdom) beamline I03, equipped with a Dectris Pilatus3 6 M detector. Diffraction data were integrated using XDS and scaled with SCALA from the CCP4 suite.^64–66^ Data collection and processing statistics are reported in Table S8. Protein crystals belong to the primitive monoclinic space group P2_1_, and their asymmetric unit includes a functional *Tb*PTR1 tetramer (Table S8, SI). Structures were solved by molecular replacement using the software Molrep and a whole enzyme tetramer (PDB code 6TBX^67^) as a search model (solvent and nonprotein atoms were excluded).^68^ All models were refined using REFMAC5 from the CCP4 suite.^66,69^ The molecular graphic software Coot was used for visual inspection, modeling, and manual rebuilding of missing atoms.^70^ The automatic placement of water molecules was performed in all structures with the ARPwARP suite.^71^ The occupancy of exogenous ligands was adjusted and refined to values resulting in atomic displacement parameters that are coherent with those of neighboring protein atoms in fully occupied sites. The final models were inspected manually and checked with the programs Coot and Procheck,^70,72^ and validated by PDB deposition. Refinement and validation statistics are displayed in Table S9 (SI). Structural models were rendered through the molecular graphic software CCP4 mg.^73^ Final coordinates and structure factors were deposited in the PDB under the codes 9HUP (*Tb*PTR1–NADP­(H)–**1a**), 9HUT (*Tb*PTR1–NADP­(H)–**2b**), 9HUU (*TbPTR1*–NADP­(H)–**2c**), 9HUV (*Tb*PTR1–NADP­(H)–**2e**), and 9HUW (*Tb*PTR1–NADP­(H)–**2d**).

## Supplementary Material







## Notes

Supplementary Figures: S1, Additional reference compounds; S2, Subpocket A of *Tb*PTR1; S3, Conformational variability of ligands and PTR1 binding site residues; S4, Halogen bond acceptor residues in PTR1s; S5, Virtual compound library with diverse linkers; S6, Crystallographic poses of previously published 2-aminobenzothiazoles; S7, *Tb*PTR1–fragment system for QM; S8, Inhibitory activity vs MED energies; S9, Ligand omit maps for ternary *Tb*PTR1–NADP­(H)–inhibitor structures; S10, Selected docking vs crystallographic poses; S11, HPLC UV–vis chromatograms. Supplementary Tables: S1, In silico ADMET properties; S2–S3, Glide SP docking scores for *Tb*PTR1 and *Lm*PTR1; S4, Measured early toxicity profiles; S5, QM *Tb*PTR1-fragment interaction energies; S6, QM binding energies vs inhibitory activities; S7, The QM His267/Trp221-fragment interaction energies; S8–S9, Supplementary crystallographic data; S10, Crystallographic cofactor/inhibitor/substrate binding loop occupancies; S11, Redocking and cross-docking results; S12, HPLC UV–vis purity evaluation. Supplementary Sections: Supplementary Methods including structure preparation for docking; QM binding energy calculations; synthesis of intermediates; liability assays; Supplementary Results including preliminary ADMET predictions and PAINS filter; general crystal structure characteristics (PDF)
Coordinates of the top 3 docked PTR1 complexes with series-1 compounds and reference compound **V** (ZIP)
Molecular formula strings (CSV)

## Notes

Atomic coordinates of crystal structures described in this paper will be released in the Protein Data Bank upon publication with identifiers: 9HUP (*Tb*PTR1–NADP­(H)– **1a**), 9HUT (*Tb*PTR1–NADP­(H)– **2b**), 9HUU (*Tb*PTR1–NADP­(H)– **2c**), 9HUV (*Tb*PTR1–NADP­(H)– **2e**), 9HUW (*Tb*PTR1–NADP­(H)– **2d**).

## Abbreviations

DHFR: dihydrofolate reductase
FBDD: fragment-based drug design
SAR: structure–activity relationship
*Lm*: *Leishmania major*
m: *meta*
p: *para*
PAINS: pan-assay interference compounds
PTR1: pteridine reductase 1
*Tb*: *Trypanosoma brucei*
