# Supplementary material for: Design of 2‑Aminobenzothiazole Derivatives Targeting Trypanosomatid PTR1 by a Multidisciplinary Fragment Hybridization Approach
Source: J Med Chem. 2025 Sep 30;68(19):20595–618. doi: 10.1021/acs.jmedchem.5c01799 (PMC12516686; doi:10.1021/acs.jmedchem.5c01799)
Supplement: Supplementary file 1 [file jm5c01799_si_001.pdf]

Supporting information
Design of 2-aminobenzothiazole derivatives targeting trypanosomatid PTR1 by a multidisciplinary fragment hybridization approach

Joanna Panecka-Hofman,[*,†,‡] Pasquale Linciano,[¶,§] Ina Pöhner,[†,||] Edyta Dyguda-Kazimierowicz,[⊥] Wiktoria Jedwabny,[⊥] Giacomo Landi,[#] Nuno Santarem,[@] Gesa Witt,[△] Bernhard Ellinger,[△] Maria Kuzikov,[△] Rosaria Luciani,[¶] Stefania Ferrari,[¶] Daniele Aiello,[¶] Stefano Mangani,[#] Cecilia Pozzi,[#,∇] Anabela Cordeiro-da-Silva,[@] Sheraz Gul,[△] Maria Paola Costi,[¶] and Rebecca C. Wade[*,†,††,‡‡]

†Molecular and Cellular Modeling Group, Heidelberg Institute for Theoretical Studies (HITS), D-69118 Heidelberg, Germany

‡Biophysics Division, Institute of Experimental Physics, Faculty of Physics, University of Warsaw, 02-093 Warsaw, Poland

¶Department of Life Sciences, University of Modena and Reggio Emilia, 41121 Modena, Italy

§Department of Drug Science, University of Pavia, 27100 Pavia, Italy

||School of Pharmacy, Faculty of Health Sciences, University of Eastern Finland, 70211 Kuopio, Finland

⊥Faculty of Chemistry, Wrocław University of Science and Technology, 50-370 Wrocław, Poland

#Department of Biotechnology, Chemistry and Pharmacy, University of Siena, 53100 Siena, Italy

@Instituto de Investigação e Inovação em Saúde, Universidade do Porto and Institute for Molecular and Cell Biology, 4150-180 Porto, Portugal

△Fraunhofer Institute for Translational Medicine and Pharmacology ITMP, Discovery Research ScreeningPort, D-22525 Hamburg, Germany

∇Consorzio Interuniversitario Risonanze Magnetiche di Metallo Proteine (CIRMMP), 50019 Sesto Fiorentino, Florence, Italy

††Center for Molecular Biology of Heidelberg University (ZMBH), DKFZ-ZMBH Alliance, and Interdisciplinary Center for Scientific Computing (IWR), Heidelberg University, D-69120, Heidelberg, Germany.

‡‡Faculties of Engineering Sciences and Biosciences, Heidelberg University, D-69120, Heidelberg, Germany.

E-mail: joanna.panecka@uw.edu.pl; rebecca.wade@h-its.org

Supplementary figures

S1 Chemical structures of additional reference compounds. S4

S2 Definition of subpocket A of *Tb*PTR1. . . . . S4

S3 Conformational variability of the ligands and the flanking residues in the PTR1 binding site. S5

S4 Location of potential halogen bond acceptor residues in the PTR1 binding site. S6

S5 Virtual compound library with diverse linkers. S7

S6 The crystallographic poses of the previously published 2-aminobenzothiazoles. S8

S7 The *Tb*PTR1–fragment system used for the QM calculations. S8

S8 Correlation plot for experimental *Tb*PTR1 activities (pK_i by Spinks et al.) versus predicted non-empirical MED energies. S9

S9 Five crystal structures of the ternary complexes of TbPTR1 with the cofactor NADP(H) and an inhibitor with omit maps. S10

S10 Docking poses of selected ligands and comparison with crystallographic poses. S11

S11 HPLC UV-vis chromatograms for the selected compounds. S12

Supplementary tables

S1 Selected ADMET properties computed with QikProp for the virtual compound library. S14

S2 Glide SP scores from docking simulations to *Tb*PTR1. . . . . S15

S3 Glide SP scores from docking simulations to *Lm*PTR1. . . . . S16

S4 Measured early toxicity profiles of the synthesized compounds. S17

S5 Total and component interaction energy values for compound fragments in *Tb*PTR1 at different levels of QM theory and with the MED model. S18

S6 Summary of calculated *Tb*PTR1–fragment QM binding energies and the measured *Tb*PTR1 inhibitory activity data. S19

S7 Interaction energy values for His267 and Trp221 residues of *Tb*PTR1 receptor and compound fragments. S20
S8 Crystallographic data collection and processing statistics. S21
S9 Crystal structure solution and refinement statistics. S22
S10 Estimated occupancy of the cofactor, inhibitor, and substrate binding loop in the five crystal structures of the *Tb*PTR1 tetramer. S23
S11 Redocking and cross-docking results of selected compounds. S24
S12 Summary of the HPLC UV-vis purity evaluation results for the representative compounds. S25

Supplementary sections

Supplementary methods **S26**
Structure preparation for docking simulations S26
The QM binding energy calculation method S27
Synthesis of intermediates S28
Liability assays S30

Supplementary results **S31**
Preliminary ADMET predictions and PAINS filter S31
General characteristics of the crystal structures of the *Tb*PTR1 complexes S32

Supplementary figures

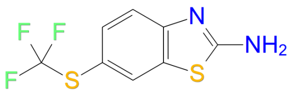

(a) **VIII**

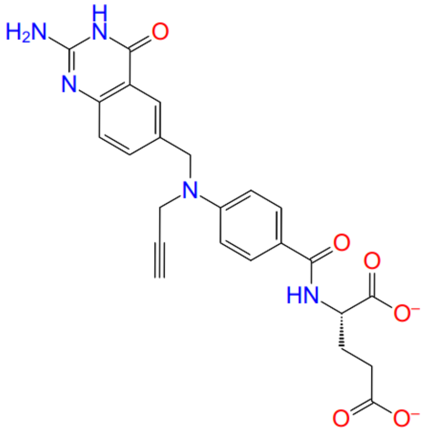

(b) **IX**

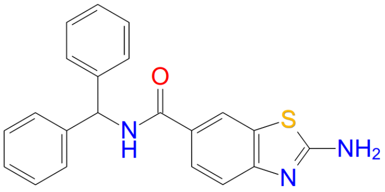

(c) **X**

Figure S1: Chemical structures of additional reference compounds mentioned in the text (supplements Tab. 1 in the main text).

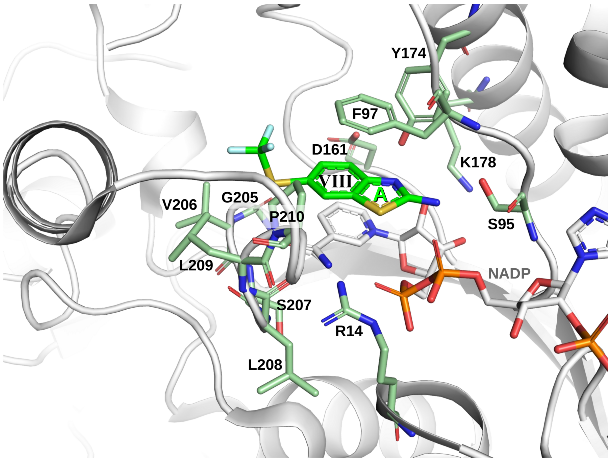

Figure S2: Definition of subpocket A of *Tb*PTR1 as in ref.[1]. The magnified view of the active site in the *Tb*PTR1-NADP$^+$ complex with compound **VIII** (PDB code 6GCQ[2]) is shown. The compound and subpocket-A protein residues are displayed as green sticks.

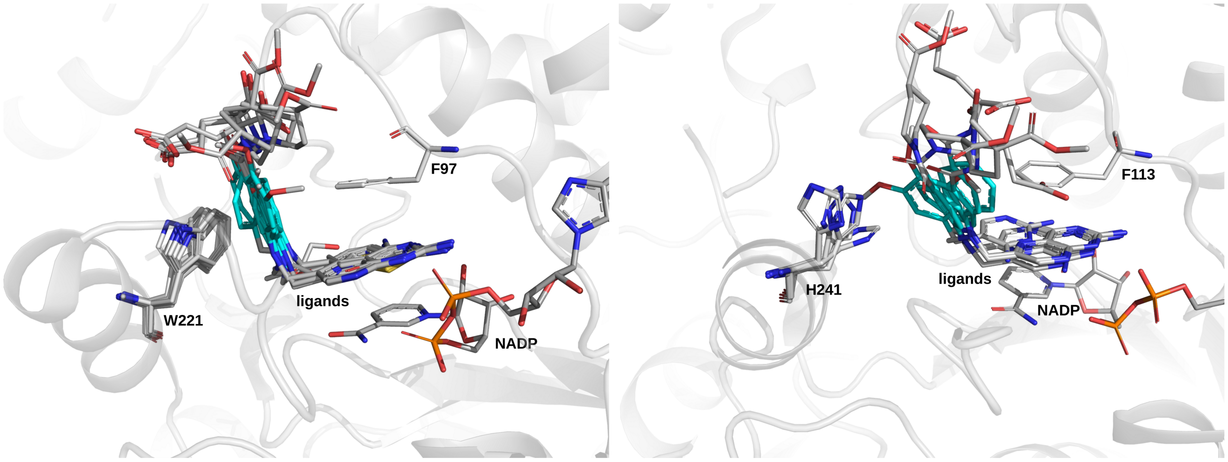

(a) (b)

Figure S3: Conformational variability of the ligands and the flanking residues in the PTR1 binding site. The conformations of ligands in aligned crystal structures together with the conformations of flanking residues Trp221 in *Tb*PTR1 (a) and His241 in *Lm*PTR1 (b) for the structures in which the ligand is bound both in the biopterin pocket (subpocket A according to ref. 1) and subpocket D/D', but not in subpocket C/C'. PDB codes: (a) 3BMC (reference), 3MCV, 6GD0, 2C7V, 2X9G, 2X9V, 6GCK, 6GCP, 6GDO, 6GEX, 6GEY, 6RX0, 6RX5, 6RX6; (b) 7PXX (reference), 1E7W, 2BFM, 2QHX, 3H4V, 6RXC. The semi-transparent cartoon, NADP and Phe97 or Phe113 are shown as sticks for the reference structure. Phenyl rings in subpocket D/D' of ligands are colored cyan.

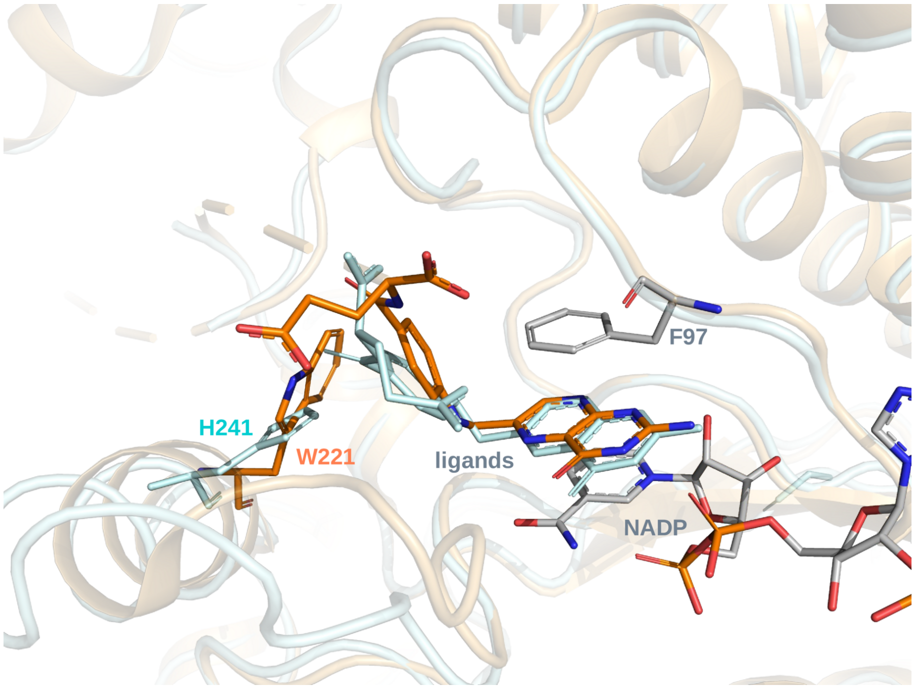

Figure S4: Aligned *Tb*PTR1 (PDB code: 3BMC, colored orange) and *Lm*PTR1 (PDB code: 7PXX, colored cyan) crystal structures showing the location of potentially halogen-bond accepting residues Trp221 and His241 (backbone carbonyl oxygens). The complexed ligands (folates), NADP and Phe97 of *Tb*PTR1 are shown in stick representation for reference.

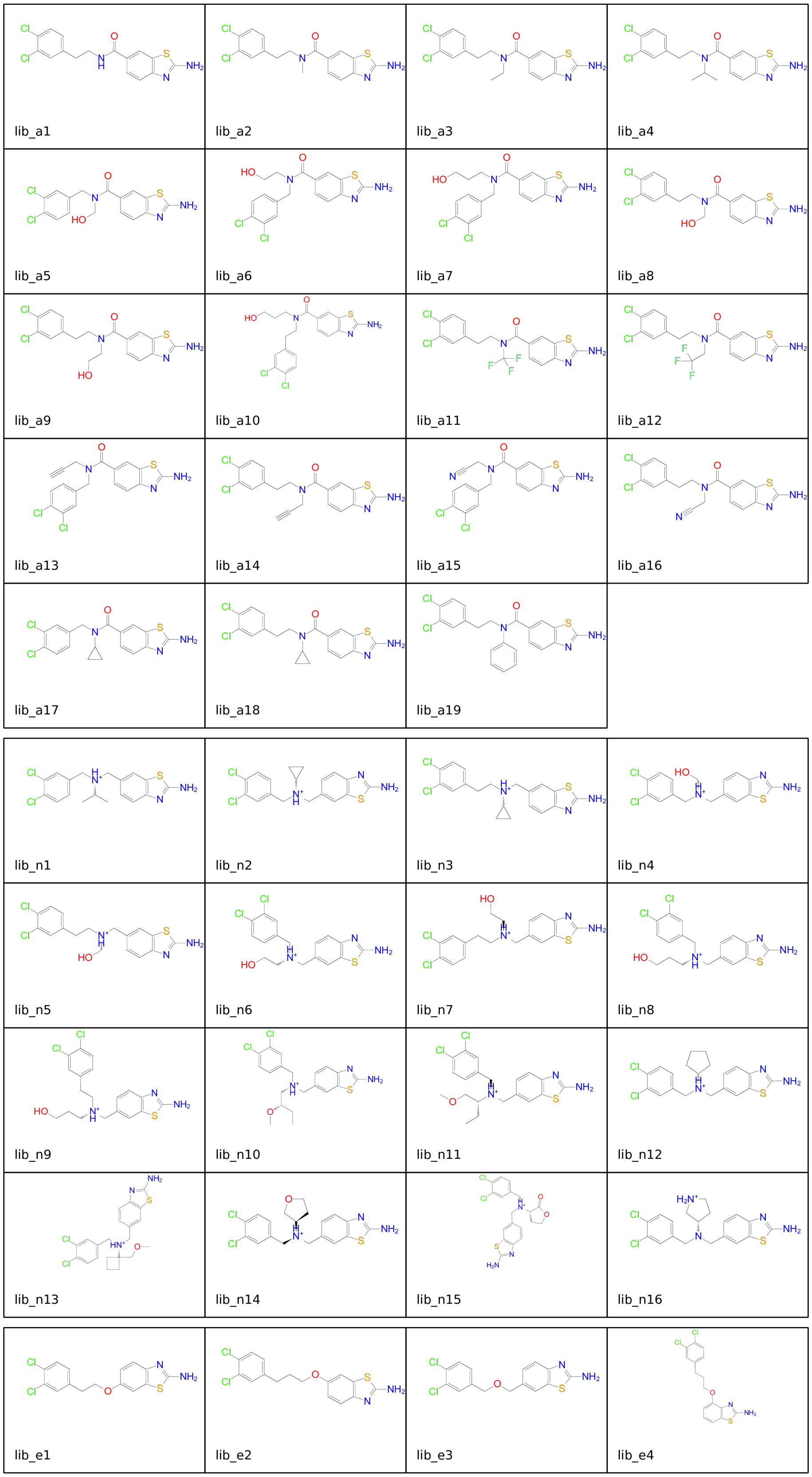

Figure S5: Virtual compound library with diverse linkers.

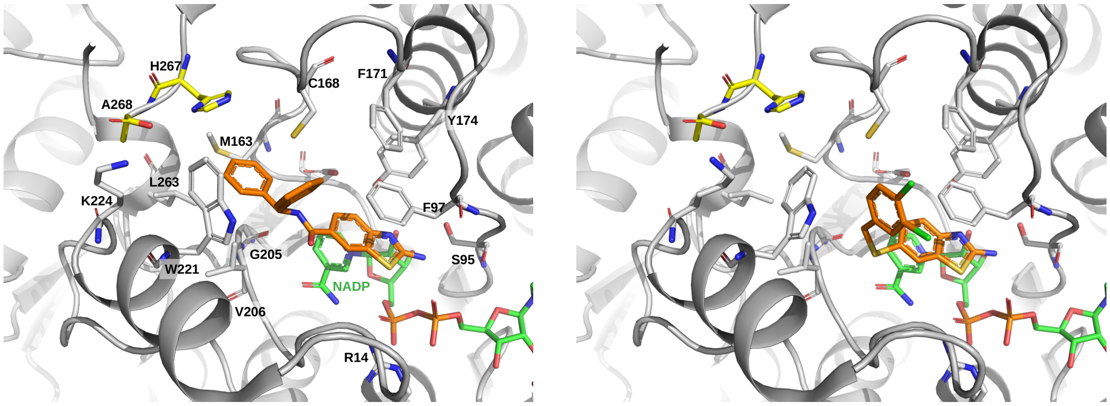

(a) (b)

Figure S6: The crystallographic poses of the previously published 2-aminobenzothiazole derivatives in *Tb*PTR1[2] (stick representation with orange carbons): (a) **X** (PDB code: 6GDP); (b) **III** (PDB code: 6GCP).

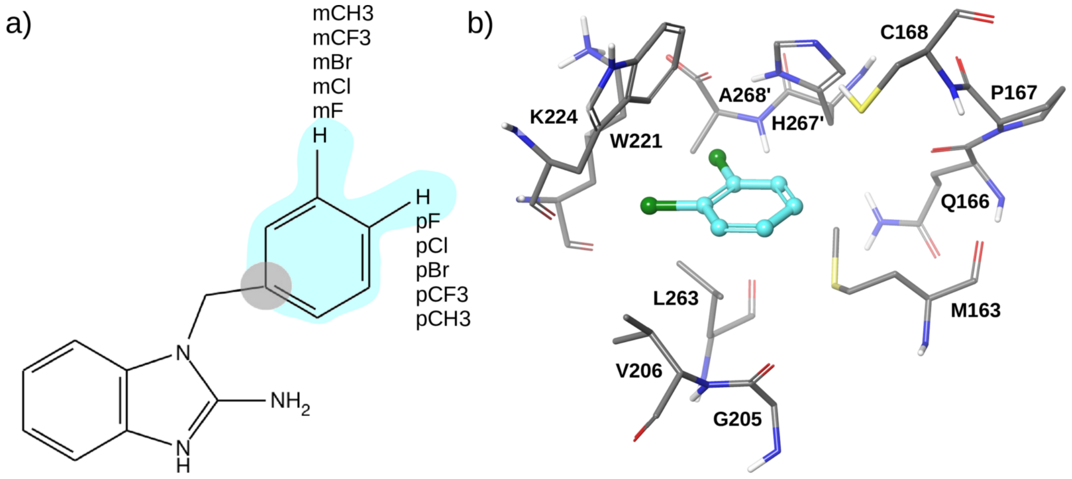

Figure S7: The *Tb*PTR1–fragment system used for the QM calculations. a) Compound **I** with the fragment used for calculations colored cyan. Positions 3 (*meta* or "m") and 4 (*para* or "p") are labelled by the substitutions considered. b) The *Tb*PTR1 binding pocket defined for the QM calculations. Only polar hydrogens are shown.

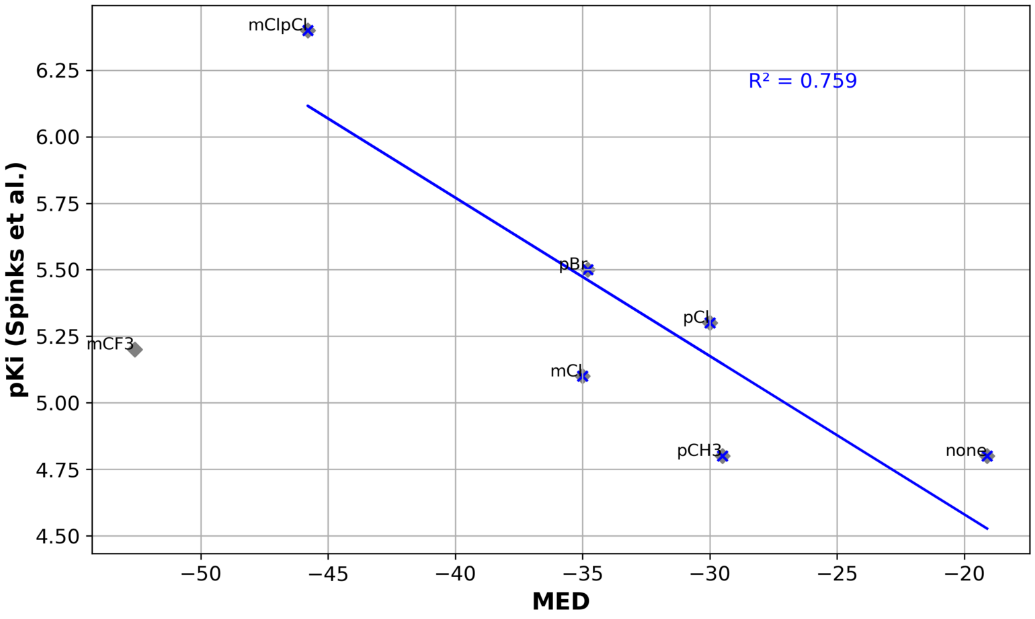

Figure S8: Correlation plot for experimental *Tb*PTR1 activities (pK_i by Spinks et al.[3]) versus predicted non-empirical MED energies (kcal/mol) for the compounds with differing substituents at the tail phenyl ring. With exclusion of mCF_3 (treated as an outlier), $R^2 = 0.759$ and $p - value = 0.024$, suggesting statistical significance at the significance level of 0.05. The data plotted are given in Tab. S6.

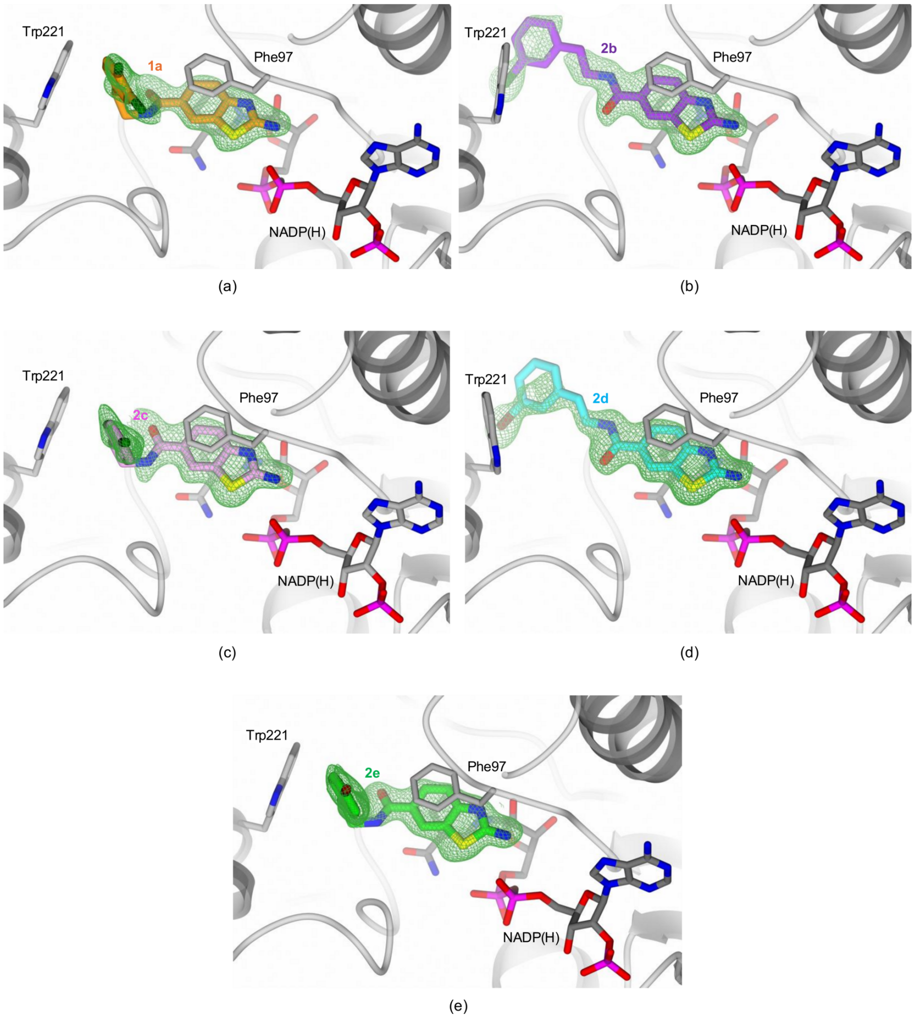

Figure S9: Five crystal structures of the ternary complexes of TbPTR1 with the cofactor NADP(H) and an inhibitor with omit maps. The active site of TbPTR1 is shown as grey cartoon with the cofactor NADP(H) in stick representation with dark gray carbons and the inhibitors in stick representation with carbons colored as follows: (a) **1a** (orange, PDB code 9HUP), (b) **2b** (purple, PDB code 9HUT), (c) **2c** (pink, PDB code 9HUU), (d) **2d** (cyan, PDB code 9HUW), and (e) **2e** (green, PDB code 9HUV). In all panels, the inhibitor is surrounded by the omit map (green mesh contoured at the 2.5σ level), oxygen atoms are colored red, nitrogen blue, sulfur yellow, phosphorus magenta, chlorine dark green, and bromine tan.

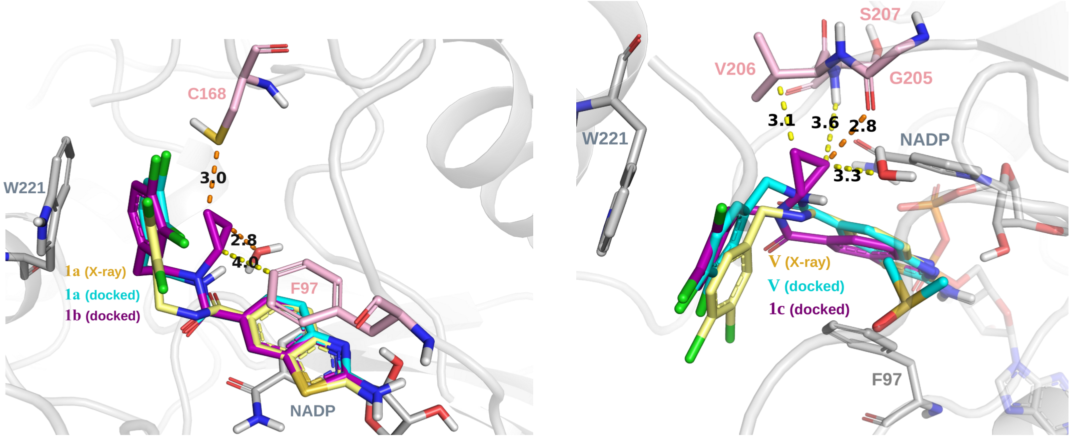

(a) (b)

Figure S10: Docking poses of selected ligands and comparison with crystallographic poses. The highest ranked (according to docking score) docked poses of (a) **1b** (pose 2) and **1a** (pose 2); and (b) **V** (pose 2) and **1c** (pose 5), resembling crystallographic poses of **1a** (PDB code: 9HUP, in pale yellow) (a) and **V** (PDB code: 6GEY, in pale yellow) (b) in *Tb*PTR1. Residues F97 and W221 are shown for orientation. The protein residues with heavy atoms within 4.5 Å of the N-cyclopropyl heavy atoms of either **1b** or **1c** are displayed (in pink) along with the conserved water molecule located near the N-cyclopropyl poses. Representative residue–residue distances are shown (in Å) and marked by dashed lines: too short distances – in orange, all other selected distances – in yellow.

1c

2a

2b

2c

2e

2f

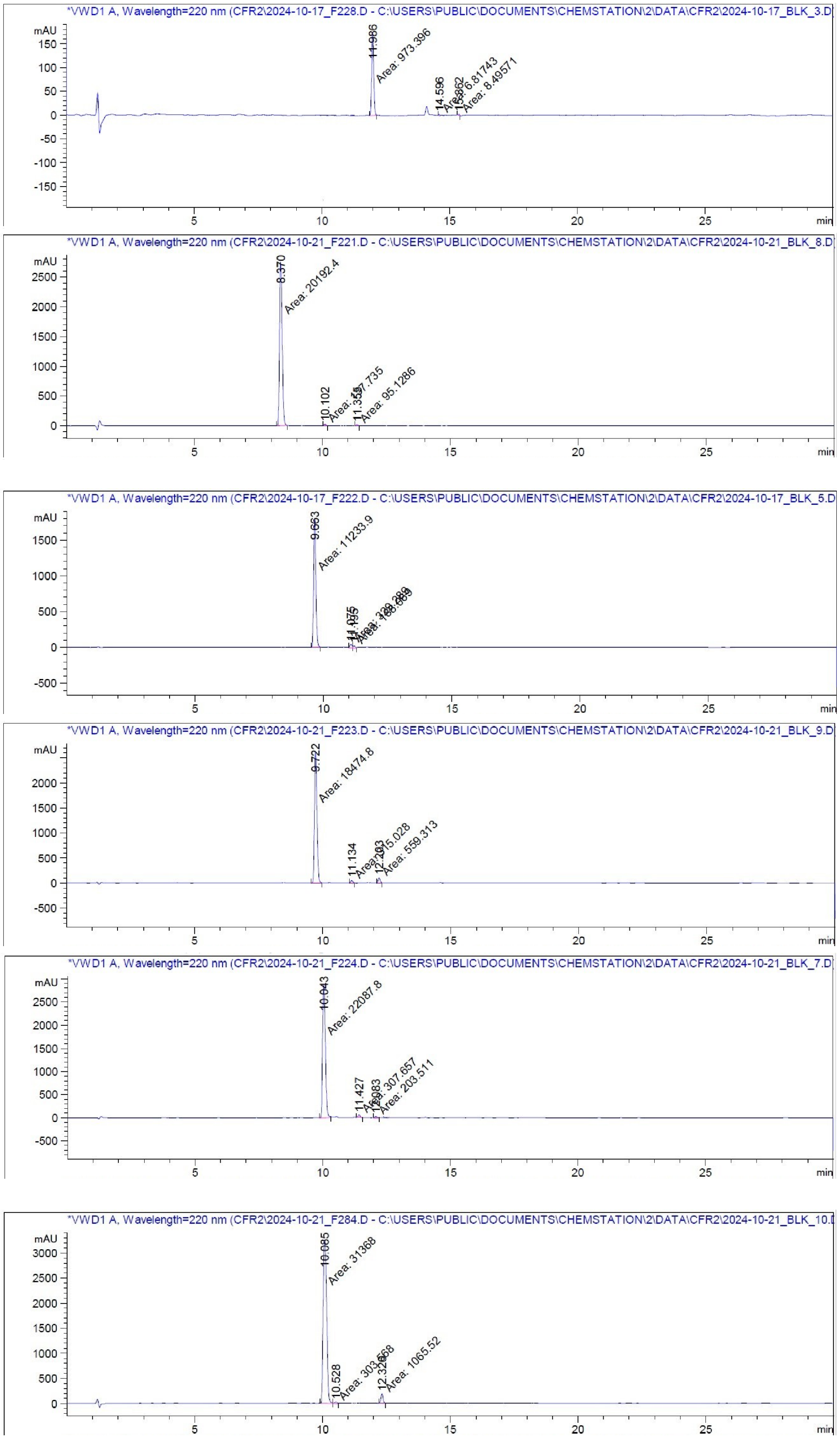

Figure S11: HPLC UV-vis chromatograms for the selected compounds.

Supplementary tables

Table S1: Selected ADMET properties computed with QikProp for the virtual compound library (see Tab. S5).

ID	linker	MW[a]	WPSA	dHB[b]	aHB[c]	R5[d]	R3[e]	#stars	#rtvFG	QPlogPo/w	QPlogS	CIQPlogS	QPlogHERG	QPPCaco	QPlogBB	QPPMDCK	#metab	QPlogKhsa	%HOA[f]
a1 (**1a**)	CONH-2C	366.264	178.851	3	4.5	0	0	1	0	3.685	-5.599	-5.567	-5.73	537.637	-0.653	2414.259	2	0.257	100
a2	CONCH3-2C	380.291	178.74	2	5	0	1	1	0	4.066	-5.95	-5.773	-5.669	709.779	-0.524	3255.147	2	0.385	100
a3	CONHC2H5-2C	394.318	178.903	2	5	0	1	1	0	4.387	-6.125	-6.055	-5.627	736.585	-0.567	3395.195	2	0.477	100
a4	CONCtProp-1C	408.345	177.616	2	5	0	1	1	0	4.637	-6.279	-6.337	-5.514	763.542	-0.535	3472.853	2	0.583	100
a5	CON-CH2OH-1C	382.264	178.555	2	5.7	0	0	1	0	3.156	-5.065	-5.551	-5.306	259.272	-0.991	1093.491	3	0.109	88.628
a6	CON-C2H4OH-1C	396.29	178.871	3	6.7	0	0	1	0	2.885	-4.969	-5.381	-5.392	211.79	-1.167	882.241	3	-0.007	85.468
a7	CON-C3H6OH-1C	410.317	178.659	3	6.7	0	0	1	0	3.157	-4.997	-5.654	-5.246	208.678	-1.205	865.926	3	0.07	86.945
a8	CON-CH2OH-2C	396.29	178.552	2	5.7	0	0	1	0	3.644	-5.524	-5.831	-5.66	322.658	-1.012	1385.045	3	0.224	93.183
a9	CON-C2H4OH-2C	410.317	178.901	3	6.7	0	0	1	0	3.302	-5.288	-5.654	-5.585	219.86	-1.24	918.98	3	0.102	88.2
a10	CON-C3H6OH-2C	424.344	178.901	3	6.7	0	1	1	0	3.66	-5.722	-5.929	-5.82	214.511	-1.379	894.838	3	0.202	90.106
a11	CN(But-2OC)C	424.387	172.261	2	5.7	0	0	0	0	4.205	-4.276	-5.347	-5.721	353.843	-0.004	1563.854	5	0.441	100
a12	CONCF3-2C	434.263	**259.149**[h]	2	5	0	1	1	0	4.538	-6.457	-6.863	-5.601	705.259	-0.324	8913.564	2	0.46	100
a13	CONCH2CF3-2C	448.289	**274.98**[h]	2	5	0	1	2	0	4.953	**-6.806**[h]	-7.148	-5.676	745.968	-0.336	10000	2	0.559	100
a14	CONCCC-1C	390.286	178.711	2.5	5	0	1	1	0	4.059	-5.766	-5.923	-5.754	704.696	-0.585	3228.762	3	0.334	100
a15	CONCCC-2C	404.313	178.657	2.5	5	0	1	1	0	4.461	-6.132	-6.204	-5.96	729.549	-0.656	3349.731	3	0.445	100
a16	CONCCN-1C	391.274	174.895	2	6.5	0	1	0	0	2.979	-5.927	-6.431	-5.16	193.947	-1.089	762.943	3	0.076	85.33
a17	CONCCN-2C	405.301	178.83	2	6.5	0	1	2	0	3.418	**-6.522**[h]	-6.709	-5.755	234.317	-1.177	983.594	3	0.158	89.375
a18 (**1c**)	CONcProp-1C	392.302	155.636	2	5	0	0	0	0	3.85	-5.078	-5.82	-4.555	801.738	-0.39	2774.549	2	0.363	100
a19 (**1b**)	CONcProp-2C	406.329	178.62	2	5	0	1	1	0	4.318	-5.753	-6.102	-5.036	695.555	-0.52	3179.864	2	0.483	100
a20 (**1e**)	CONPh-2C_Ph	442.362	174.314	2	5	1	1	1	0	5.244	**-6.946**[h]	-7.387	-6.477	741.447	-0.598	3227.099	3	0.769	96.063
n1+[g]	CNtProp-C	380.334	178.593	2	4	0	0	1	0	4.141	-4.26	-5.098	-5.387	353.876	0.235	1694.044	4	0.548	96.809
n1	CNtProp-C	380.334	178.852	2	4	0	0	1	0	4.18	-4.504	-5.098	-5.751	353.872	0.202	1699.565	4	0.55	100
n2+ (**1d**)	CNcProp-1C	378.318	134.898	2	4	0	0	0	0	3.585	-3.078	-4.863	-4.45	439.197	0.293	1232.986	4	0.383	95.235
n2 (**1d**)	CNcProp-1C	378.318	134.076	2	4	0	0	0	0	3.616	-3.27	-4.863	-4.723	426.333	0.258	1181.685	4	0.393	95.185
n3+	CNcProp-CC	392.345	178.88	2	4	0	0	1	0	4.389	-4.69	-5.148	-5.801	350.816	0.122	1684.296	4	0.604	100
n3	CNcProp-CC	392.345	175.585	2	4	0	0	1	0	4.417	-4.827	-5.148	-5.981	351.073	0.096	1617.016	4	0.615	100
n4+	CN-CH2OH-1C	368.28	178.881	3	5.7	0	0	1	0	2.682	-3.454	-4.141	-5.892	135.849	-0.293	604.057	5	0.009	80.826
n4	CN-CH2OH-1C	368.28	178.91	3	5.7	0	0	1	0	2.792	-3.423	-4.141	-5.936	189.998	-0.142	868.386	5	0.005	84.078
n5+	CN-CH2OH-2C	382.307	178.888	3	5.7	0	0	1	0	3.172	-4.076	-4.414	-6.37	140.816	-0.413	628.019	5	0.141	83.974
n5	CN-CH2OH-2C	382.307	178.922	3	5.7	0	0	1	0	3.121	-4.044	-4.414	-6.471	146.288	-0.405	654.718	5	0.109	83.975
n6+	CN-C2H4OH-1C	382.307	174.499	3	5.7	0	0	0	0	3.181	-3.7	-4.414	-5.851	174.313	-0.251	748.346	5	0.147	85.686
n6	CN-C2H4OH-1C	382.307	178.91	3	5.7	0	0	1	0	2.901	-3.749	-4.414	-6.089	104.33	-0.509	454.269	5	0.083	80.06
n7+	CN-C2H4OH-2C	396.334	178.884	3	5.7	0	0	1	0	3.419	-4.135	-4.69	-6.267	138.566	-0.469	617.151	5	0.208	85.294
n7	CN-C2H4OH-2C	396.334	177.861	3	5.7	0	0	1	0	3.333	-4.161	-4.69	-6.385	104.737	-0.611	450.194	5	0.203	82.616
n8+	CN-C3H6OH-1C	396.334	176.519	3	5.7	0	0	1	0	3.215	-3.664	-4.69	-5.71	103.458	-0.514	436.795	5	0.19	81.828
n8	CN-C3H6OH-1C	396.334	175.858	3	5.7	0	0	1	0	3.252	-3.796	-4.69	-5.897	105.263	-0.536	441.34	5	0.195	82.184
n9+	CN-C3H6OH-2C	410.36	178.882	3	5.7	0	0	1	0	3.537	-4.235	-4.967	-6.188	103.482	-0.669	450.12	5	0.263	83.719
n9	CN-C3H6OH-2C	410.36	178.927	3	5.7	0	0	1	0	3.689	-4.684	-4.967	-6.706	103.248	-0.752	449.279	5	0.299	84.588
n10	CN(But-2OC)C	424.387	172.261	2	5.7	0	0	0	0	4.205	-4.276	-5.347	-5.721	353.843	-0.004	1563.854	5	0.441	100
n10+	CN(But-2OC)C	424.387	177.682	2	5.7	0	0	1	0	4.297	-4.533	-5.347	-5.876	353.877	-0.013	1674.686	5	0.469	100
n11+	CN(Prop-1COC)C	424.387	168.803	2	5.7	0	0	0	0	4.368	-4.272	-5.347	-5.512	353.858	0.011	1497.161	5	0.521	100
n11	CN(Prop-1COC)C	424.387	165.528	2	5.7	0	0	0	0	4.389	-4.249	-5.347	-5.462	353.88	0.009	1436.67	5	0.536	100
n12+	CN(cPent)C	406.372	134.093	2	4	0	0	0	0	4.136	-3.769	-5.604	-4.717	459.158	0.285	1280.602	4	0.605	100
n12	CN(cPent)C	406.372	138.791	2	4	0	0	0	0	4.173	-4.063	-5.604	-5.103	438.224	0.247	1291.934	4	0.608	100
n13+	CN(cBut-1COC)C	436.398	134.73	2	4.7	0	0	0	0	4.315	-3.935	-5.714	-5.015	458.027	0.134	1287.499	5	0.59	100
n13	CN(cBut-1COC)C	436.398	133.432	2	4.7	0	0	0	0	4.2	-3.597	-5.714	-4.759	455.675	0.155	1259.557	5	0.547	100
n14+	CN(cPent 3-O)C	408.345	132.98	2	5.7	0	0	0	0	3.237	-2.78	-5.065	-4.34	461.638	0.318	1270.12	5	0.224	93.583
n14	CN(cPent 3-O)C	408.345	135.911	2	5.7	0	0	0	0	3.221	-2.743	-5.065	-4.235	449.919	0.32	1281.823	5	0.222	93.29
n15+	CN(cPent 2C=O 3-O)C	422.328	134.037	2	7	0	0	0	1	2.467	-2.499	-4.914	-4.361	163.59	-0.061	419.419	5	0.046	81.012
n15	CN(cPent 2C=O 3-O)C	422.328	139.721	2	7	0	0	0	1	2.529	-2.802	-4.914	-4.739	154.518	-0.117	423.646	5	0.056	80.934
n16	CN(cPent 3-N)C	407.36	138.012	3	5.5	0	0	0	0	2.593	-2.127	-3.972	-5.747	60.367	0.415	166.084	4	0.256	74.002
e1	O-2C	339.239	178.795	2	2.75	0	1	1	0	4.415	-5.702	-5.77	-5.699	1418.177	-0.191	6883.208	3	0.439	100
e2	O-3C	353.265	178.796	2	2.75	0	1	1	0	4.797	-6.134	-6.057	-5.898	1418.173	-0.278	6883.324	3	0.558	100
e3	1C-O-1C	339.239	178.668	2	3.7	0	0	1	0	3.959	-5.132	-5.426	-5.305	1418.835	-0.153	6875.68	4	0.261	100
e4	alt-O-3C	353.265	178.861	2	2.75	0	1	1	0	4.811	-6.114	-6.057	-5.92	1525.988	-0.243	7456.749	3	0.552	100
**I**	-	292.167	129.636	2	2	0	0	0	0	3.807	-4.386	-5.074	-4.747	2019.087	0.07	5424.171	1	0.299	100
**II**	O-1C	325.212	178.789	2	2.75	0	0	1	0	4.057	-5.283	-5.484	-5.501	1418.173	-0.101	6882.689	3	0.329	100
**V**	CONH-1C	352.237	178.847	3	4.5	0	0	1	0	3.257	-5.072	-5.29	-5.287	517.678	-0.541	2317.44	2	0.147	94.592

[a] molecular weight; [b] number of hydrogen bond donors; [c] number of hydrogen bond acceptors; [d] Rule of Five; [e] Jorgensen's Rule of Three; [f] % of human oral absorption; [g] the compound states with positively charged amines are marked by "+"; [h] the values mentioned in the text are in bold.

Table S2: Glide SP scores (in kcal/mol) from docking simulations to *Tb*PTR1 for the synthesized compounds (series 1) and two reference compounds.

**compound**	**pose no.**	**docking score**	**Glide Evdw**[a]	**Glide Ecoul**[b]	**Glide Einternal**[c]	**Emodel**[d]
**1a**	1	-10.2	-41.1	-10.1	6.0	-84.0
	2	-9.4	-46.9	-8.8	3.4	-87.2
	3	-9.4	-47.0	-8.7	3.3	-87.1
**1b**	1	-9.3	-45.0	-6.5	6.6	-81.5
	2	-8.7	-45.2	-8.1	13.7	-77.5
	3	-8.6	-41.6	-6.1	11.1	-67.9
**1c**	1	-10.1	-50.2	-8.5	11.5	-87.7
	2	-9.5	-47.0	-8.1	16.8	-81.3
	3	-9.4	-48.0	-8.0	14.6	-80.9
**1d**	1	-10.2	-50.3	-10.4	9.6	-98.7
	2	-10.0	-46.3	-12.4	20.7	-90.6
	3	-10.0	-45.2	-12.3	11.7	-90.2
**1e**	1	-9.0	-45.8	-3.0	8.3	-71.8
	2	-8.9	-31.3	-9.7	11.8	-61.8
	3	-8.6	-43.35	-8.8	13.1	-71.5
			**Reference compounds**			
**II**	1	-8.7	-46.3	-4.7	7.9	-74.6
	2	-8.5	-43.7	-7.2	7.8	-74.1
	3	-8.4	-40.9	-6.4	3.5	-72.3
**V**	1	-9.3	-47.1	-6.4	5.1	-82.2
	2	-9.1	-46.0	-9.1	2.1	-84.6
	3	-8.5	-41.6	-5.7	3.8	-73.8

[a] van der Waals energy contribution to docking score; [b] electrostatic energy contribution to docking score; [c] ligand internal energy; [d] energy score, the terms of which are weighted such that it more accurately ranks the conformers of a single ligand than the docking score.

Table S3: Glide SP scores (in kcal/mol) from docking simulations to *Lm*PTR1 of the synthesized compounds (series 1) and two reference compounds.

compound	pose no.	docking score	Glide Evdw	Glide Ecoul	Glide Einternal	Emodel
**1a**	1	-8.8	-39.1	-8.2	4.1	-72.4
	2	-8.8	-39.0	-8.2	3.9	-71.9
	3	-8.5	-38.9	-8.9	5.7	-71.2
**1b**	1	-8.1	-40.8	-8.5	14.8	-66.7
	2	-8.0	-41.8	-6.0	17.9	-62.9
	3	-7.9	-43.6	-6.6	13.8	-67.7
**1c**	1	-7.2	-36.9	-6.0	10.1	-59.8
	2	-7.1	-38.0	-5.2	8.5	-60.6
	3	-7.0	-39.9	-6.6	11.9	-61.2
**1d**	1	-8.4	-39.3	-9.1	8.1	-71.0
	2	-8.1	-41.9	-7.3	3.7	-72.7
	3	-8.0	-42.3	-7.3	2.2	-74.4
**1e**	1	-7.1	-47.9	-3.0	14.4	-61.1
	2	-6.7	-21.2	-8.3	11.2	-40.3
	3	-6.5	-47.3	-1.7	11.2	-59.2
**Reference compounds**						
**II**	1	-8.2	-37.4	-8.7	6.7	-66.6
	2	-8.1	-36.7	-8.6	6.6	-65.8
	3	-8.1	-38.1	-8.3	6.8	-66.7
**V**	1	-8.3	-38.5	-9.5	2.2	-70.5
	2	-8.1	-37.4	-9.4	1.7	-68.9
	3	-7.1	-36.4	-4.6	0.4	-60.9

[a] van der Waals energy contribution to docking score; [b] electrostatic energy contribution to docking score; [c] ligand internal energy; [d] energetic score, the terms of which are weighted such that it more accurately ranks conformers of a single ligand than docking score.

Table S4: Measured early toxicity profiles of the synthesized compounds and compound **I** as a reference. The following values are given in μM units: IC_{50} against hERG, CYP isoforms, and mitochondria (mitotox); GI_{50} (50% growth inhibition); TGI (total growth inhibition); LC_{50} (50% lethal concentration) against A549 cell line; cytotoxic concentration for THP1 cells (CC_{50}). Abbreviations: max – maximum concentration or "out of range"; nd – not determined. The corresponding values given as % residual activity at 10 μM compound concentration are in square brackets.

	**CYP**					**A549 cytotoxicity**				
**Cmpd**	**1A2**	**2C9**	**2C19**	**2D6**	**3A4**	**GI_{50}**	**TGI**	**LC_{50}**	**mitotox.**	**hERG**
**Reference**										
**I**	29.9	4.2	0.3	0.0	3.1	nd	nd	nd	38.6	0.85
**Series 1**										
**1a**	max	max	8.5	0.1	1.0	27.0	54.4	max	27.9	max
**1b**	max	5.0	4.7	0.9	0.7	13.8	54.6	max	0.6	max
**1c**	max	48.3	2.9	2.5	1.3	24.6	61.3	100	3.8	max
**1d**	67.1	8.8	1.2	2.5	4.0	12.6	34.4	63.1	1.5	max
**1e**	[37.7%]	[35.3%]	[31.1%]	[59.9%]	[26.3%]	[102.6%]	nd	nd	[-33.8%]	[-34.8%]
**Series 2**										
**2a**	100	37.7	25.6	max	19.9	max	max	max	max	4.5
**2b**	max	35.2	6.3	0.9	6.5	54.5	95.9	max	max	2.8
**2c**	max	9.6	4.2	0.5	max	max	max	max	40.0	4.5
**2d**	max	74.5	2.9	5.0	5.4	60.4	85.5	max	max	3.2
**2e**	max	3.6	2.5	0.1	4.7	max	max	max	0.2	3.2
**2f**	nd	nd	nd	nd	nd	nd	nd	nd	nd	nd
**2g**	nd	nd	nd	nd	nd	nd	nd	nd	nd	nd

Table S5: Total and component interaction energy values (kcal/mol) for compound fragments in *Tb*PTR1 at different levels of QM theory and with the MED model[4,5]. These are shown in Fig. 9 (main text).

**Fragment substituents**[a]	$E^{(10)}_{EL,MTP}$	$E^{(10)}_{EX}$	D_{as}	E_{MED}[b]
none	0.0	14.5	-19.2	-19.1
mFpF	-2.3	16.1	-23.2	-25.5
mClpCl	-4.5	50.1	-41.2	-45.8
mBrpBr	-6.1	86.3	-50.3	-56.4
mCF3pCF3	-5.3	235.9	-80.7	-86.1
mCH3pCH3	0.1	48.7	-40.8	-40.7
mF	-3.1	15.9	-21.4	-24.5
mCl	-4.9	34.7	-30.1	-35.0
*mCl (**2b**)*[c]	*-5.6*	*44.3*	*-36.9*	*-42.5*
mBr	-6.5	58.8	-35.1	-41.7
*mBr (**2d**)*[c]	*-5.0*	*80.7*	*-44.9*	*-49.9*
mCF3	-6.6	98.4	-46.0	-52.6
mCH3	-0.8	34.4	-29.9	-30.8
pF	0.7	14.7	-21.0	-20.3
pCl	-0.1	27.9	-29.9	-30.0
pBr	-0.2	45.0	-34.6	-34.8
pCF3	0.3	99.6	-49.4	-49.1
pCH3	0.7	29.5	-30.2	-29.5

[a] The fragment definition and naming is shown in Fig. S7a and detailed in *Methods*. [b] E_{MED} energy is the sum of $E^{(10)}_{EL,MTP}$ and D_{as} energies. [c] The calculation results for the mCl and mBr fragments positioned as in chains A of the *Tb*PTR1 complexes with **2b** (PDB code 9HUT) and **2d** (PDB code 9HUW), respectively.

Table S6: Summary of calculated *Tb*PTR1–fragment QM binding energies (kcal/mol) and the measured *Tb*PTR1 inhibitory activity data from the current work and the work of Spinks et al.[3].

**Fragment substituents**	$E_{MED}{}^a$	**pIC$_{50}$^{b} (this work)**	**pKi (Spinks et al.[3])^c**
none	-19.1	4.8	4.8
mClpCl	-45.8	5.1	6.4
mCl	-35	5.4	5.1
mBr	-41.7	5.6	nd^d
mCF3	-52.6	5.2	5.2
pCl	-30	5.3	5.3
pBr	-34.8	5.5	5.5
pCH3	-29.5	nd^d	4.8
pCF3	-49.1	5.2	nd^d

^{a} The full set of the computed E_{MED} energies are given in Tab. S5. ^{b} The pIC$_{50}$ values (μM) are calculated for the current 2-aminobenzothiazole compound series (Tab. 2 in the main text). ^{c} The pKi values (μM) were measured by Spinks et al. for derivatives of **I**[3]. ^{d} "nd" – no data available.

Table S7: Interaction energy values (kcal/mol) for His267 and Trp221 residues of *Tb*PTR1 receptor and compound fragments at different levels of QM theory and with the MED model[4,5].

Residue	Fragment substituents	$E^{(10)}_{EL,MTP}$	$E^{(10)}_{EX}$	D_{as}	E_{MED}
**His267**	**none**	0.7	1.3	-2.8	-2.1
	mF	0.6	1.3	-2.8	-2.3
	mCl	0.5	1.3	-2.9	-2.4
	**2b** (mCl)	0.2	13.5	-8.5	-8.3
	mBr	0.5	1.3	-2.9	-2.4
	mCF3	0.5	1.5	-3.1	-2.6
	mCH3	0.8	1.5	-3.0	-2.2
	pF	-0.9	1.5	-3.2	-4.1
	pCl	-0.8	4.9	-5.4	-6.2
	**2d** (pCl)	1.5	11.4	-7.6	-6.1
	pBr	-0.9	8.3	-6.4	-7.2
	pCF3	-1.4	30.7	-12.1	-13.5
	pCH3	0.6	6.3	-6.0	-5.4
	mFpF	-1.0	1.5	-3.3	-4.2
	mClpCl	-0.7	4.9	-5.5	-6.2
	mBrpBr	-0.8	8.6	-6.5	-7.3
	mCF3pCF3	-1.3	53.3	-15.1	-16.4
	mCH3pCH3	0.2	7.8	-6.5	-6.3
**Trp221**	**none**	0.8	2.6	-3.8	-3.0
	mF	0.4	4.1	-5.2	-4.8
	mCl	-1.6	20.0	-10.7	-12.3
	**2b** (mCl)	0.1	18.7	-12.1	-12.0
	mBr	-2.9	41.7	-14.7	-17.6
	mCF3	-1.7	70.3	-19.4	-21.0
	mCH3	-0.2	18	-9.9	-10.2
	**2d** (pCl)	-1.7	33.0	-13.9	-15.6
	pF	0.4	2.3	-4.1	-3.7
	pCl	0.4	3.8	-5.4	-5.0
	pBr	0.4	5.0	-5.8	-5.4
	pCF3	-0.3	17.6	-9.4	-9.6
	pCH3	1.3	4.2	-5.4	-4.2
	mFpF	0.2	4.0	-5.5	-5.4
	mClpCl	-1.9	24.4	-13.1	-14.9
	mBrpBr	-3.1	42.5	-16.5	-19.5
	mCF3pCF3	-1.8	124.3	-29.0	-30.9
	mCH3pCH3	1.6	17.8	-11.0	-9.4

Table S8: Crystallographic data collection and processing statistics; values for the outer shell are given in parentheses.

	***Tb*PTR1 + NADP(H) +**				
	**1a**	**2b**	**2c**	**2d**	**2e**
**PDB code**	9HUP	9HUT	9HUU	9HUW	9HUV
**Diffraction source**	DLS I03	DLS I03	DLS I03	DLS I03	DLS I03
**Wavelength (Å)**	0.9762	0.9762	0.9762	0.9762	0.9196
**Temperature (K)**	100	100	100	100	100
**Detector**	Pilatus3 6M	Pilatus3 6M	Pilatus3 6M	Pilatus3 6M	Pilatus3 6M
**Crystal-detector distance (mm)**	313.0	313.0	313.0	313.0	312.6
**Rotation range per image (°)**	0.25	0.25	0.25	0.25	0.25
**Total rotation range (°)**	200	200	200	200	200
**Exposure time per image (s)**	0.15	0.15	0.15	0.15	0.15
**Space group**	$P2_1$	$P2_1$	$P2_1$	$P2_1$	$P2_1$
**No. of subunits in asymmetric unit**	4 (1 tetramer)	4 (1 tetramer)	4 (1 tetramer)	4 (1 tetramer)	4 (1 tetramer)
**a, b, c (Å)**<br>β (°)	74.33, 90.50, 83.10<br>115.43	74.34, 90.78, 83.21<br>115.47	74.55, 90.58, 83.14<br>115.62	74.40, 90.53, 82.81<br>115.43	74.30, 89.61, 82.60<br>115.33
**Mosaicity (°)**	0.12	0.17	0.18	0.15	0.11
**Resolution range (Å)**	90.50–1.76 (1.86–1.76)	90.77–1.84 (1.94–1.84)	57.75–1.85 (1.95–1.85)	90.35–1.80 (1.90–1.80)	89.61–1.58 (1.67–1.58)
**Total no. of reflections**	352966 (52961)	311894 (47562)	247874 (35701)	341245 (50903)	503522 (73118)
**No. of unique reflections**	97143 (14196)	84980 (12503)	84345 (12263)	90332 (13073)	130968 (18830)
**Completeness (%)**	98.8 (99.2)	98.3 (99.4)	99.2 (99.3)	98.8 (98.3)	98.1 (97.0)
**Redundancy**	3.6 (3.7)	3.7 (3.8)	2.9 (2.9)	3.8 (3.9)	3.8 (3.9)
**$\langle I/\sigma(I) \rangle$**	12.9 (2.0)	12.0 (2.1)	11.6 (2.0)	12.9 (2.1)	13.7 (2.6)
**R_{meas}**	0.065 (0.816)	0.061 (0.673)	0.057 (0.610)	0.054 (0.759)	0.057 (0.631)
**Overall B factor from Wilson plot (Å^2)**	26.3	32.3	27.2	31.6	21.2

Table S9: Crystal structure solution and refinement statistics; values for the outer shell are given in parentheses.

	***Tb*PTR1 + NADP(H) +**				
	**1a**	**2b**	**2c**	**2d**	**2e**
**PDB code**	9HUP	9HUT	9HUU	9HUW	9HUV
**Resolution range (Å)**	90.50–1.76 (1.81–1.76)	90.77–1.84 (1.89–1.84)	57.75–1.85 (1.90–1.85)	90.35–1.80 (1.85–1.80)	89.61–1.58 (1.62–1.58)
**Completeness (%)**	98.70 (99.00)	98.19 (99.22)	99.19 (99.28)	98.58 (98.09)	97.93 (96.73)
**No. of reflections, working set**	92122 (6867)	80735 (5997)	80137 (5897)	85762 (6283)	124306 (9012)
**No. of reflections, test set**	4991 (339)	4218 (332)	4190 (314)	4541 (336)	6631 (516)
**Final R_{cryst}**	0.194 (0.362)	0.198 (0.328)	0.180 (0.349)	0.190 (0.471)	0.171 (0.341)
**Final R_{free}**	0.239 (0.380)	0.253 (0.346)	0.231 (0.348)	0.233 (0.469)	0.198 (0.322)
**No. of non-H atoms**					
Protein	7362	7402	7452	7354	7482
Ligands (NADP(H), compound)	253 (192, 61)	280 (192, 88)	258 (192, 66)	258 (192, 66)	241 (192, 49)
Other (acetate anion, ethylene glycol)	8 (0, 0, 8)	8 (0, 0, 8)	16 (4, 1, 12)	20 (0, 0, 20)	16 (8, 0, 8)
Water	540	485	540	504	673
Total	8163	8175	8266	8136	8412
**R.m.s. deviations**					
Bonds (Å)	0.008	0.008	0.008	0.008	0.010
Angles (°)	1.557	1.558	1.528	1.539	1.703
**Average B factors (Å^2)**	32.9	36.8	31.2	35.2	29.0
Protein	32.5	36.8	30.6	34.9	28.2
Ligands (NADP(H), compound)	41.1	41.6	37.7	38.7	34.4
Other (acetate anion, ethylene glycol)	47.6	38.8	40.6	53.1	38.0
Water	42.4	43.2	40.9	45.3	40.9
**Estimate error on coordinates based on R value (Å)**	0.13	0.15	0.14	0.14	0.08
**Ramachandran plot**					
Most favored (%)	95.9	95.6	96.6	96.4	96.8
Allowed (%)	4.1	4.4	3.4	3.6	3.2
**RSCC**					
NADP(H) (chain)	0.96(A), 0.92(B), 0.75(C), 0.95(D)	0.96(A), 0.93(B), 0.86(C), 0.95(D)	0.96(A), 0.94(B), 0.83(C), 0.96(D)	0.96(A), 0.94(B), 0.83(C), 0.95(D)	0.96(A), 0.94(B), 0.80(C), 0.95(D)
Compound (chain)	0.83(A), 0.82(B), 0.83(C)	0.91(A), 0.94(B), 0.83(C), 0.92(D)	0.91(A), 0.88(B), 0.88(D)	0.92(A), 0.90(B), 0.87(D)	0.85(A), 0.81(B), 0.83(D)

Table S10: Estimated occupancy of the cofactor, inhibitor, and substrate binding loop (residues 207–218) in the four chains composing the functional *Tb*PTR1 tetramer in the five crystal structures.

	***Tb*PTR1 + NADP(H) +**				
	**1a**	**2b**	**2c**	**2d**	**2e**
**Subunit A**					
Inhibitor occupancy (phenyl tail occupancy)	90% (50%)	90% (70%)	90% (60%)	80% (60%)	90% (60%)
Cofactor occupancy	90%	90%	90%	90%	90%
Substrate binding loop occupancy	100%	100%	100%	100%	100%
**Subunit B**					
Inhibitor occupancy (phenyl tail occupancy)	80% (50%)	90% (70%)	80% (60%)	80% (60%)	80% (phenyl tail not modelled)
Cofactor occupancy	80%	90%	80%	80%	80%
Substrate binding loop occupancy	100%	100%	100%	100%	100%
**Subunit C**					
Inhibitor occupancy (phenyl tail occupancy)	Lack of inhibitor binding	70% (50%)	Lack of inhibitor binding	Lack of inhibitor binding	Lack of inhibitor binding
Cofactor occupancy	70%	70%	60%	50%	60%
Substrate binding loop occupancy	70% (residues 210-212 not modelled)	70%	70%	60% (residues 211-217 not modelled)	70%
**Subunit D**					
Inhibitor occupancy (phenyl tail occupancy)	80% (phenyl tail not modelled)	90% (70%)	90% (60%)	80% (60%)	80% (phenyl tail not modelled)
Cofactor occupancy	90%	90%	90%	90%	90%
Substrate binding loop occupancy	100%	100%	100%	100%	100%

Table S11: Redocking and cross-docking results of selected compounds binding in the biopterin subpocket and subpockets C and D of *Tb*PTR1. Chain A of the reference structures was considered. The RMSD values of the ligand heavy atoms are given.

**Ligand class (Ligand PDB code)**	**Ref. structure PDB code**	**RMSD (Å)**	**Comment**
7-deazaguanine 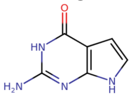	9QDK	0.29	original ligand (redocking)
thiadiazole (WHF) 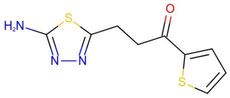	2YHU	1.24	
thiadiazole (3KH) 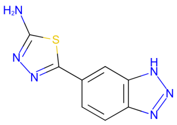	4WCD	0.58	
thiadiazole (3KN) 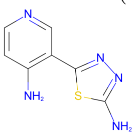	4WCF	0.45	
thiadiazole (6F4) 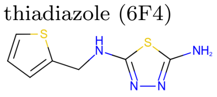	5IZC	1.43	the tail thiophene ring rotation observed in chain B with respect to A, RMSD =0.96 Å
benzothiazole (6KT) 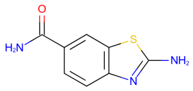	6GD4	1.06	small compound, no tail
benzothiazole (EWT/**V**) 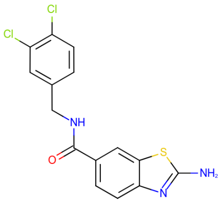	6GEY	1.66	the score for pose 2; shifted 3,4-dichlorophenyl tail

Table S12: Summary of the HPLC UV-vis purity evaluation results for the representative compounds (chromatograms are shown in Fig. S11). t_r – retention time, MW – molecular weight, nd – no data.

				Purity (%)	
**Compound**	**MW**	**HRMS m/z [M+H]$^+$**	t_r **(min)**	**at 220 nm**	**at 254 nm**
**1c**	392.3022	Calcd 392.0391<br>Found 392.0381	11.986	98.45	nd
**2a**	297.3748	Calcd 298.1014<br>Found 298.1006	8.37	98.85	97.76
**2b**	331.8199	Calcd 332.0624<br>Found 332.0613	9.603	95.59	97.21
**2c**	331.8199	Calcd 332.0624<br>Found 332.0615	9.722	95.48	95.63
**2e**	376.2709	Calcd 376.0119<br>Found 376.0110	10.043	97.73	98.54
**2f**	365.3728	Calcd 366.0888<br>Found 366.0877	10.085	95.81	96.78

Supplementary methods

Structure preparation for docking simulations

Computational preparation of the protein receptors. The following PTR1 structures were used for docking simulations: for *Tb*PTR1 – PDB code 9QDK in the complex with the ligand **I** occupying subpockets C and D and a fragment 7-deazaguanine in the biopterin pocket; for *Lm*PTR1 – PDB code 1E92 (in complex with 7,8-dihydrobiopterin).

The PTR1 homotetramers were prepared with PrepWizard from the Maestro suite[6]. The active site in chain A was selected. During the preparation process, all the $NADP^+$ cofactors, specifically-bound ligand (or ligands) in chain A and waters within 8 Å of the ligands were kept. The missing side-chains were modelled using Prime (in the Maestro suite). The cofactor and ligand protonation states were generated with Epik (Maestro) at pH 7±0.5, $NADP^+$ phosphates were kept deprotonated. Protein protonation states were calculated with PropKa at pH 7[7–9] and the hydrogen bonding network was optimized. Due to the presence of the ligands during receptor preparation, the hydroxyl group of the $NADP^+$ ribose is directed towards the biopterin pocket, allowing for the formation of a hydrogen bond present in many PTR1 complexes. Hydrogen atom positions were energy minimized. Finally, any ligands and water molecules were removed from the binding pocket. For the main docking receptors, the conserved water sites were calculated as described previously for *Tb*PTR1 and *Lm*PTR1, based on the crystallographic data[10].

Preparation of compound structures. 3-dimensional structures of the compounds were built in Maestro[6] and then prepared with the LigPrep workflow (Maestro). Ionization states and tautomers were generated at pH 7.0±2.0 with the Epik tool (Maestro suite). Up to 32 stereoisomers and up to two low-energy ring conformations (when needed) were generated per compound. The titratable moieties were present only in the linkers, mostly amines, for which the protonated and deprotonated variants were considered. For the compounds

containing amide bonds, both *trans* and *cis* isomers of peptide bonds in ligands were allowed.

Computational prediction of ADMET properties. Prediction of ADMET properties, including $\log P_{octanol/water}$, aqueous solubility (logS), estimated passage of the blood-brain barrier (logBB), estimated passive transport for cell lines such as Caco-2 or MDCK (models of the gut-blood and the blood-brain barrier, respectively), binding to human serum albumin (logKhsa) or blockage of hERG K^+ channels was carried out with QikProp[6]. Additionally, PAINS filtering was performed on compounds selected for synthesis with the web server PAINS-Remover (https://www.cbligand.org/PAINS/).

The QM binding energy calculation method

The multipole electrostatic component is obtained from the Cumulative Atomic Multipole Moments[11] (CAMM) expansion with a modified version[12] of GAMESS program[13] according to the following equation:

$$E^{(10)}_{\mathrm{EL,MTP}} = \sum_{a\in A}\sum_{b\in B}\sum_{k_a}\sum_{k_b}\sum_{\alpha}\sum_{\beta} \boldsymbol{M}^{\alpha}_{a}[k_a]\boldsymbol{T}^{k_a+k_b}_{\alpha\beta}\boldsymbol{M}^{\beta}_{b}[k_b], \tag{1}$$

where $\boldsymbol{M}^{\alpha}_{a}[k_a]$ and $\boldsymbol{M}^{\beta}_{b}[k_b]$ are the α and β components of atom-centered multipole tensors of rank k_a and k_b for interacting molecules A and B, respectively, and $\boldsymbol{T}^{k_a+k_b}_{\alpha\beta}$ is the $\alpha\beta$ element of the Cartesian interaction tensor containing the partial derivatives of $|\boldsymbol{R}_{ab}|^{-1}$ of rank $k_a + k_b$ ($\boldsymbol{R}_{ab}$ represents the distances between a and b atoms). The superscript one present in $E^{(10)}_{EL,MTP}$ denotes the first-order perturbation theory. The zero in the superscript implies that the multipole moments are calculated from Hartree-Fock (HF) densities obtained with the polarized triple zeta valence basis set of Ahlrichs et al. (def2TZVP)[14,15].

The D_{as} function[4,16,17] is expressed as the sum of the following atom-atom terms:

$$D_{\mathrm{as}} = \sum_{a\in A,\, b\in B}\Big(- \frac{\sqrt{C^6_a C^6_b}}{(r_{ab})^6} f_6\big(\sqrt{\beta_a\beta_b} r_{ab}\big) - \frac{\sqrt{C^8_a C^8_b}}{(r_{ab})^8} f_8\big(\sqrt{\beta_a\beta_b} r_{ab}\big)\Big) \tag{2}$$

where a and b denote atoms in monomers A and B, respectively, and $f_n(r)$ is the Tang-Toennies[18] damping function:

$$f_n(r) = 1 - e^{-r} \sum_{i=0}^{n} \frac{r^i}{i!} \tag{3}$$

The parameters C_x^n and β_x, $x = a, b$ are fitted to the sum of the SAPT(DFT)[19–22] dispersion ($E^{(2)}_{\text{disp}}$) and exchange-dispersion ($E^{(2)}_{\text{exch-disp}}$) energy values, $E^{(2)}_{\text{dispx}}$:

$$E^{(2)}_{\text{dispx}} = E^{(2)}_{\text{disp}} + E^{(2)}_{\text{exch-disp}} \tag{4}$$

where the superscript (2) in energy terms indicates the second-order perturbation theory contributions.

Synthesis of intermediates

Synthesis of 2-(3,4-dichlorophenyl)acetic acid (4). A solution of 2-(3,4-dichlorophenyl)acetonitrile (500 mg, 2.69 mmol, 1 equiv.) in 37% aqueous HCl (10 mL) was refluxed for 6 hours, monitored by TLC until the starting material was no longer visible. After refluxing, the solution was cooled to room temperature and neutralized with solid $NaHCO_3$ until effervescence ceased. The aqueous solution was then washed with diethyl ether (Et_2O). Subsequently, it was acidified with 1N HCl to pH 1 to precipitate the product. The solid was collected by filtration, rinsed with water, and dried, yielding 395 mg (72% yield) of a white solid. This product was used in the subsequent step without further purification. ^{1}H NMR (400 MHz, DMSO-d_6) δ (ppm) 12.53 (br s, 1H), 7.56 (d, J = 8.4 Hz, 1H), 7.55 (d, J = 1.6 Hz, 1H), 7.26 (dd, J = 2.0 Hz, 8.0 Hz, 1H) 3.63 (s, 2H). HRMS m/z [M-H]$^-$ Calcd for $C_8H_6Cl_2O_2$: 202.9667, found: 202.9671.

Synthesis of N-cyclopropyl-2-(3,4-dichlorophenyl)acetamide (5). To a solution of **4** (250 mg, 1.23 mmol, 1 equiv.) in anhydrous DMF under a nitrogen atmosphere at 0°C,

EDC·HCl (235 mg, 1.23 mmol, 1 equiv.) and HOBt (166 mg, 1.23 mmol, 1 equiv.) were added. The mixture was stirred at 0°C for 10 minutes. Subsequently, cyclopropylamine (85 μL, 1.23 mmol, 1 equiv.) was added, followed by the addition of TEA (150 μL, 1.23 mmol, 1 equiv.). The temperature was then allowed to rise spontaneously, and the mixture was stirred overnight at room temperature. After completion, the DMF was removed under reduced pressure, and the residue was suspended in water and extracted with ethyl acetate. The organic layer was washed three times with a saturated solution of Na_2CO_3 and then with brine, dried over anhydrous Na_2SO_4, and concentrated to give 258 mg (86% yield) of a pale-yellow liquid, pure enough to be used in the next step. ^{1}H NMR (400 MHz, DMSO-d_6) δ (ppm) 8.14 (br s, 1H), 7.62 (d, J = 8.4 Hz, 1H), 7.56 (d, J = 1.6 Hz, 1H), 7.29 (dd, J = 2.0 Hz, 8.0 Hz, 1H), 3.85 (s, 2H), 2.60-2.80 (m, 1H), 0.76-0.84 (m, 2H), 0.51-0.62 (m, 2H). HRMS m/z $[M+H]^+$ Calcd for $C_{11}H_{12}Cl_2NO$: 244.0290, found: 244.0254.

Synthesis of N-(3,4-dichlorophenethyl)cyclopropanamine (3b). To a solution of **5** (250 mg, 1.03 mmol, 1 equiv.) in anhydrous THF (10 mL) at 0°C and under nitrogen atmosphere, 1M $LiAlH_4$ in THF (1.23 mL, 1.23 mmol, 1.2 equiv.) was added dropwise. The mixture was refluxed for 3 hours and cooled at room temperature. A saturated solution of NH_4Cl was carefully added to quench the unreacted $LiAlH_4$ and the white suspension obtained was filtered through a celite pad and the filtrate was concentrated. The residue was suspended in AcOEt and the organic phase was subsequently washed with a saturated solution of Na_2CO_3, a saturated solution of NH_4Cl, and brine. The organic phase was dried over anhydrous Na_2SO_4 and concentrated to give 82 mg (32% yield) of a pale-yellow liquid, which was directly used in the next step without further purification. ^{1}H NMR (400 MHz, DMSO-d_6) δ 0.57 – 0.82 (m, 4H), 2.25 (p, J = 9.2 Hz, 1H), 2.71 (t, 2H), 2.84 (t, 2H), 7.02 (d, J = 7.5 Hz, 1H), 7.32 (d, J = 1.4 Hz, 1H), 7.62 (d, J = 7.4 Hz, 1H). HRMS m/z $[M+H]^+$ Calcd for $C_{11}H_{14}Cl_2N$: 230.0498, found: 230.0431.

Synthesis of N-(3,4-dichlorophenethyl)aniline (3c). To a solution of 2-(3,4-dichlorophenyl)ethan-1-amine (500 mg, 2.63 mmol, 1 equiv.) in DCM, phenyl boronic acid (320 mg, 2.63 mmol, 1 equiv.) and copper acetate (1% mol) were added. The mixture was stirred at room temperature for 1 hour and concentrated. The residue was directly purified over silica gel (crude:silica gel ratio 1:100, eluent DCM:MeOH 9:1) to give 370 mg (53% yield) of a pale yellow waxy solid. ^{1}H NMR (400 MHz, $CDCl_3$) δ 2.93 (d, 2H), 3.40 (d, 2H), 6.58 (d, 2H), 6.67 (d, 1H), 7.02-7.07 (m, 3H), 7.32 (s, 1 H), 7.62 (d, 2H). HRMS m/z $[M+H]^+$ Calcd for $C_{14}H_{14}Cl_2N$: 266.0498, found: 266.0503.

Synthesis of N-(3,4-dichlorobenzyl)cyclopropanamine (3d). To a solution of cyclopropylamine (500 mg, 8.78 mmol, 2 equiv.), in DMF at room temperature, K_2CO_3 (1500 mg, 11 mmol, 2.5 equiv.) and 3,4-dichlorobenzyl bromide (638 μL, 4.4 mmol, 1 equiv.) were added. The suspension was stirred in the same conditions overnight. The solvent was removed under reduced pressure, the residue was suspended in AcOEt and the organic phase was washed with a saturated solution of Na_2CO_3, and brine. The organic phase was dried over anhydrous Na_2SO_4 and concentrated. The crude was purified over silica gel (crude:silica gel ratio 1:120, eluent DCM:MeOH 9:1) to give 750 mg (83% yield) of a pale yellow liquid. ^{1}H NMR (400 MHz, $CDCl_3$) δ 0.57-0.82 (m, 4H), 2.25 (m, 1H), 3.76 (s, 2H), 6.58 (d, 2H), 7.20 (d, 1H), 7.40 (s, 1H), 7.65 (d, 2H). HRMS m/z $[M+H]^+$ Calcd for $C_{10}H_{12}Cl_2N$: 216.0341, found: 213.0354.

Liability assays

The hERG cardiotoxicity assay. The Invitrogen Predictor hERG fluorescence polarization (FP) assay was utilized with a membrane fraction containing hERG (Predictor hERG membrane). Displacement of a red fluorescent high-affinity hERG ligand (Predictor hERG Tracer Red) from the hERG channel due to binding of the test compound was determined in an FP-based format[23].

Cytochrome P450 (CYP450) assays against isoforms 1A2, 2C9, 2C19, 2D6, and 3A4. The Promega P450-Glo assay platform was used with microsomal preparations of cytochrome P450s from baculovirus-infected insect cells. In this assay, light is emitted when a CYP450 enzyme metabolizes its substrate and a reduction in light emission indicates that the tested compound inhibits the specific isoform[23].

Mitochondrial toxicity caused by the synthesized compounds. Uptake of Mito-Tracker Red (chloromethyl-X-rosamine) to the 786-O cell line was detected with high content imaging. Cells were cultured in Roswell Park Memorial Institute (RPMI)-1640 medium supplemented with 2 mM glutamine, FCS (10% v/v), streptomycin (100 μg/mL), and penicillin G (100 U/mL)[23].

Cytotoxicity against A549 cells. The CellTiter-Glo assay from Promega was used. It detects the cellular ATP content, which is directly proportional to the number of viable cells. The A549 cells were obtained from DSMZ (German Collection of Microorganisms and Cell Cultures, Braunschweig, Germany) and cultured in Dulbecco's modified Eagle medium (DMEM) with FCS (10% v/v), streptomycin (100 μg/mL), and penicillin G (100 U/mL)[23].

Supplementary results

Preliminary ADMET predictions and PAINS filter

The screened compounds showed no major problems in the *in silico* evaluated ADMET properties (see Tab. S1). In most cases, one QikProp 'star' (indicating potential problems) was assigned, and it was mostly due to the high weakly polar solvent-accessible surface area (SASA), which was close to the upper limit (range 0.0 - 175.0 $Å^2$). However, this issue was also observed for the previously synthesized compounds, e.g., **II** and **V**[2] (Tab. 1, main text). There were two exceptions, for which the weakly polar SASA value was significantly higher

than the upper bound: **lib_a12** – 259 Å^2 and **lib_a13** – 275 Å^2. These compounds were not selected for chemical synthesis. For three compounds (**lib_a13**, **lib_a17** and **lib_a20/1e**), aqueous solubility was indicated as a potential problem, but the QPlogS values were within 0.5 of the lower limit of -6.5. Finally, none of the considered compounds violated Lipinski's Rule of Five, while single violations were observed for Jorgensen's Rule of Three due to the insufficient aqueous solubility (requirement logS >-5.7 was not met). In subsequent experiments, no solubility issues were observed for the synthesized compounds. Finally, PAINS filtering was performed[24] on all compounds selected for chemical synthesis, and did not flag any compound.

General characteristics of the crystal structures of the *Tb*PTR1 complexes

The four enzyme subunits were completely resolved apart from two solvent-exposed loops (including residues 104–113 and 143–151) that are usually poorly structured in *Tb*PTR1 [23,25–29]. The cofactor NADP(H), naturally co-purified with the enzyme, binds in an extended conformation (see Fig. 10, main text), stabilized by a tight network of highly conserved interactions, as was observed previously[25,29]. The population of the cofactor site typically ranges from 80% to 90% in all complexes, and is distinctly lower (50–70%) in one chain of the tetramer (chain C in our models), where the cofactor is less well structured (Tab. S10). The formation of the holoenzyme is essential for properly shaping the substrate binding pocket, in which the substrate (or an inhibitor) is accommodated: the substrate pterin moiety binds in a π-sandwich lined by the NADP(H) nicotinamide on one side and the phenyl ring of Phe97 on the other[25,29] (Fig. 10, main text).

A further characteristic feature of the *Tb*PTR1 active site is the flexible substrate-binding loop (residues 207–218), which forms stabilizing interactions with both cofactor and substrate (or inhibitors). Former structural investigations on *Tb*PTR1 evidenced a direct relationship between the population of the cofactor site and the flexibility of the substrate loop[23,26,27].

Consistently, in chain C of all complexes, the NADP(H) site is occupied to a lower extent (50–70%) than in the other subunits, which is associated with a relatively low occupancy for substrate loop (60–70%) and, typically, the lack of inhibitor binding (Tab. S10). The only exception is the ternary complex with compound **2b**, in which the inhibitor populates the active site of all four subunits of the *Tb*PTR1 tetramer – in chain C, the inhibitor occupancy was estimated to be 70%, similar to the cofactor and the substrate loop (Tab. S10). It should also be noted that conformations of the PTR1 substrate loops are likely affected by crystal packing interactions[30], which might influence conformations and occupancies of inhibitors.

The 2-aminobenzothiazole core occupies the *Tb*PTR1 biopterin binding pocket, stacking with Phe97 and the cofactor nicotinamide (Fig. 10a-e, main text), and forms polar interactions with the surrounding residues and the cofactor. Its benzothiazole nitrogen receives a hydrogen bond from the NADP(H) ribose hydroxyl and the amine moiety donates to the Ser95 hydroxyl and the cofactor β-phosphate (Fig. 10a-e, main text). In the complexes with **1a** and **2e**, a water-mediated interaction additionally connects the benzothiazole sulfur with the NADP(H) β-phosphate and the guanidinium group of Arg14 (Fig. 10a,e, main text).

References

(1) Panecka-Hofman, J.; Poehner, I.; Spyrakis, F.; Zeppelin, T.; Di Pisa, F.; Dello Iacono, L.; Bonucci, A.; Quotadamo, A.; Venturelli, A.; Mangani, S.; Costi, M. P.; Wade, R. C. Comparative mapping of on-targets and off-targets for the discovery of anti-trypanosomatid folate pathway inhibitors. *Biochim. Biophys. Acta Gen. Subj.* **2017**, *1861*, 3215–3230.

(2) Linciano, P. et al. Enhancement of Benzothiazoles as Pteridine Reductase-1 Inhibitors for the Treatment of trypanosomatidic Infections. *J. Med. Chem.* **2019**, *62*, 3989–4012.

(3) Spinks, D.; Ong, H. B.; Mpamhanga, C. P.; Shanks, E. J.; Robinson, D. A.; Collie, I. T.; Read, K. D.; Frearson, J. A.; Wyatt, P. G.; Brenk, R.; Fairlamb, A. H.; Gilbert, I. H. Design, synthesis and biological evaluation of novel inhibitors of *Trypanosoma brucei* pteridine reductase 1. *ChemMedChem* **2011**, *6*, 302–308.

(4) Pernal, K.; Podeszwa, R.; Patkowski, K.; Szalewicz, K. Dispersionless density functional theory. *Phys. Rev. Lett.* **2009**, *103*, 263201.

(5) Giedroyc-Piasecka, W.; Dyguda-Kazimierowicz, E.; Beker, W.; Mor, M.; Lodola, A.; Sokalski, W. A. Physical Nature of Fatty Acid Amide Hydrolase Interactions with Its Inhibitors: Testing a Simple Nonempirical Scoring Model. *J. Phys. Chem. B* **2014**, *118*, 14727–14736.

(6) Schrodinger, Schrödinger Suite 2015-2, Protein Preparation Wizard, Epik version 3.2, Impact version 6.7, Prime version 4.0. 2015.

(7) Li, H.; Robertson, A.; Jensen, J. Very Fast Empirical Prediction and Interpretation of Protein pKa Values. *Proteins* **2005**, *61*, 704–721.

(8) Olsson, M.; Søndergard, C.; Rostkowski, M.; Jensen, J. PROPKA3: Consistent Treat-

ment of Internal and Surface Residues in Empirical pKa predictions. *J. Chem. Theory Comput.* **2011**, *7*, 525–537.

(9) Søndergard, C.; Olsson, M.; Rostkowski, M.; Jensen, J. Improved Treatment of Ligands and Coupling Effects in Empirical Calculation and Rationalization of pKa Values. *J. Chem. Theory Comput.* **2011**, *7*, 2284–2295.

(10) Poehner, I. et al. Multitarget, Selective Compound Design Yields Potent Inhibitors of a Kinetoplastid Pteridine Reductase 1. *J. Med. Chem.* **2022**, *65*, 9011–9033.

(11) Sokalski, W. A.; Poirier, R. A. Cumulative atomic multipole representation of the molecular charge distribution and its basis set dependence. *Chem. Phys. Lett.* **1983**, *98*, 86–92.

(12) Langner, K. M. Nonempirical methods in the analysis and electrostatic modeling of biomolecular interactions. Ph.D. thesis, Wroclaw University of Science and Technology, 2010.

(13) Schmidt, M. W.; Baldridge, K. K.; Boatz, J. A.; Elbert, S. T.; Gordon, M. S.; Jensen, J. H.; Koseki, S.; Matsunaga, N.; Nguyen, K. A.; Su, S. J.; Windus, T. L.; Dupuis, M.; Montgomery, J. A. General atomic and molecular electronic structure system. *J. Comput. Chem.* **1993**, *14*, 1347–1363.

(14) Weigend, F. Accurate Coulomb-fitting basis sets for H to Rn. *Phys. Chem. Chem. Phys.* **2006**, *8*, 1057–1065.

(15) Weigend, F.; Ahlrichs, R. Balanced basis sets of split valence, triple zeta valence and quadruple zeta valence quality for H to Rn: Design and assessment of accuracy. *Phys. Chem. Chem. Phys.* **2005**, *7*, 3297–3305.

(16) Podeszwa, R.; Pernal, K.; Patkowski, K.; Szalewicz, K. Extension of the Hartree-Fock

plus dispersion method by first-order correlation effects. *J. Phys. Chem. Lett.* **2010**, *1*, 550–555.

(17) Jedwabny, W.; Dyguda-Kazimierowicz, E.; Pernal, K.; Szalewicz, K.; Patkowski, K. Extension of an Atom–Atom Dispersion Function to Halogen Bonds and Its Use for Rational Design of Drugs and Biocatalysts. *J. Phys. Chem. A* **2021**, *125*, 1787–1799.

(18) Tang, K. T.; Toennies, J. P. An improved simple model for the van der Waals potential based on universal damping functions for the dispersion coefficients. *J. Chem. Phys.* **1984**, *80*, 3726–3741.

(19) Misquitta, A. J.; Szalewicz, K. Intermolecular forces from asymptotically corrected density functional description of monomers. *Chem. Phys. Lett.* **2002**, *357*, 301–306.

(20) Misquitta, A. J.; Jeziorski, B.; Szalewicz, K. Dispersion energy from density-functional theory description of monomers. *Phys. Rev. Lett.* **2003**, *91*, 033201.

(21) Misquitta, A. J.; Szalewicz, K. Symmetry-adapted perturbation-theory calculations of intermolecular forces employing density-functional description of monomers. *J. Chem. Phys.* **2005**, *122*, 214109.

(22) Misquitta, A. J.; Podeszwa, R.; Jeziorski, B.; Szalewicz, K. Intermolecular potentials based on symmetry-adapted perturbation theory with dispersion energies from time-dependent density-functional calculations. *J. Chem. Phys.* **2005**, *123*, 214103.

(23) Borsari, C. et al. Profiling of Flavonol Derivatives for the Development of Antitrypanosomatidic Drugs. *J. Med. Chem.* **2016**, *59*, 7598–7616.

(24) Baell, J. B.; Holloway, G. A. New substructure filters for removal of pan assay interference compounds (PAINS) from screening libraries and for their exclusion in bioassays. *J. Med. Chem.* **2010**, *53*, 2719–2740.

(25) Dawson, A.; Gibellini, F.; Sienkiewicz, N.; Tulloch, L. B.; Fyfe, P. K.; McLuskey, K.; Fairlamb, A. H.; Hunter, W. N. Structure and reactivity of *Trypanosoma brucei* pteridine reductase: inhibition by the archetypal antifolate methotrexate. *Mol. Microbiol.* **2006**, *61*, 1457–1468.

(26) Linciano, P. et al. Exploiting the 2-Amino-1,3,4-thiadiazole Scaffold To Inhibit Pteridine Reductase in Support of Early-Stage Drug Discovery. *ACS Omega* **2017**, *2*, 5666–5683.

(27) Di Pisa, F. et al. Chroman-4-One Derivatives Targeting Pteridine Reductase 1 and Showing Anti-Parasitic Activity. *Molecules* **2017**, *22*.

(28) Landi, G.; Linciano, P.; Borsari, C.; Bertolacini, C. P.; Moraes, C. B.; Cordeiro-da Silva, A.; Gul, S.; Witt, G.; Kuzikov, M.; Costi, M. P.; Pozzi, C.; Mangani, S. Structural Insights into the Development of Cycloguanil Derivatives as *Trypanosoma brucei* Pteridine-Reductase-1 Inhibitors. *ACS Infect. Dis.* **2019**, *5*, 1105–1114.

(29) Pozzi, C.; Tassone, G.; Mangani, S. In *Neglected Diseases: Extensive Space for Modern Drug Discovery*; Botta, M., Ed.; Annual Reports in Medicinal Chemistry; Academic Press, 2018; Chapter X-Ray Crystallography Contributions to Drug Discovery Against Parasite., pp 175–230.

(30) Panecka-Hofman, J.; Poehner, I. Structure and dynamics of pteridine reductase 1: the key phenomena relevant to enzyme function and drug design. *Eur. Biophys. J.* **2023**, *52*, 521–532.
